# The Allen–Cahn equation with weakly critical random initial datum

**DOI:** 10.1007/s00440-024-01312-1

**Published:** 2024-08-09

**Authors:** Simon Gabriel, Tommaso Rosati, Nikos Zygouras

**Affiliations:** 1 University of Münster, Münster, DE Germany; 2 University of Warwick, Coventry, UK

**Keywords:** Allen–Cahn equation, White noise, Critical SPDE, B-series, 60H15, 35R60

## Abstract

This work considers the two-dimensional Allen–Cahn equation

$$\begin{aligned} \partial _t u = \frac{1}{2}\Delta u + \mathfrak {m}\, u -u^3, \quad u(0,x)= \eta (x), \qquad \forall (t,x) \in [0, \infty ) \times {\textbf {R}}^{2}, \end{aligned}$$

where the initial condition $\eta$ is a two-dimensional white noise, which lies in the scaling critical space of initial data to the equation. In a weak coupling scaling, we establish a Gaussian limit with nontrivial size of fluctuations, thus casting the nonlinearity as marginally relevant. The result builds on a precise analysis of the Wild expansion of the solution and an understanding of the underlying stochastic and combinatorial structure. This gives rise to a representation for the limiting variance in terms of Butcher series associated to the solution of an ordinary differential equation.

## Introduction

We consider the two-dimensional Allen–Cahn equation

1.1 $$\begin{aligned} \partial _t u = \frac{1}{2}\Delta u + \mathfrak {m}\, u -u^3, \quad u(0,\cdot )= \eta ( \cdot ), \quad t \geqslant 0, \end{aligned}$$

with $\mathfrak {m} \in {\textbf {R}}$ and initial condition a spatial white noise $\eta$ on ${\textbf {R}}^{2}$, namely a homogeneous Gaussian random field with correlation function

$$\begin{aligned} \begin{aligned} \mathbb {E}[\eta (x) \eta (y)] = \delta (x-y), \qquad \forall x, y \in {\textbf {R}}^{2}, \end{aligned} \end{aligned}$$

where $\delta$ is the Dirac delta at zero. Under these assumptions, there exists no solution theory for (1.1), since the initial condition is scaling critical for the equation. By this we mean that formally (assuming the solution *u* exists and setting for simplicity $\mathfrak {m} =0$), under the re-scaling $u^{\delta }(t, x) := \delta u ( \delta ^{2} t, \delta x)$, which leaves the initial condition invariant in law, $u^{\delta }$ solves again

1.2 $$\begin{aligned} \begin{aligned} \partial _{t} u^{\delta } = \frac{1}{2} \Delta u^{\delta } - (u^{\delta })^{3}. \end{aligned} \end{aligned}$$

The invariance under this re-scaling indicates that the nonlinearity cannot be treated perturbatively and so for instance modern theories on singular SPDEs do not apply, since they work under the assumption that on small scales the equation is governed by the effect of the Laplacian. From a deterministic perspective, invariance under re-scaling indicates that the initial condition lies, up to an infinitesimal loss of regularity, in the critical space of initial data, which in the case of this equation corresponds to the Hölder–Besov space $\mathcal {C}^{- 1}({\textbf {R}}^{d})$. While some equations can be solved deterministically for all subcritical initial data, (1.1) is locally well-posed only for initial data with regularity $\mathcal {C}^{ - \alpha }( {\textbf {R}}^{d})$, for $\alpha < \tfrac{2}{3}$, and ill-posed otherwise [12].

Obtaining a solution theory to (1.1) is therefore challenging but also of great interest. Indeed, if $\mathfrak {m} > 0$, then (1.1) is a model for the formation of phase fields, since $\mathfrak {m} u - u^{3}$ corresponds to the gradient of a double-well potential and the solution tends (for large times) to take the value of one of the two minima of the potential, leading to the evolution of two competing phases. Here, starting the equation at a generic initial condition should lead to many conjectured long-time properties, for instance regarding the speed of coarsening of sets in the evolution of mean curvature flow (see [3, 31] from the physics literature and [25, 28] for some mathematical results and a discussion of the problem). In this context, space white noise plays the role of a canonical “totally mixed” initial condition, which gets instantaneously smoothened by the heat flow, leading to random level sets which then evolve under mean curvature flow.

On the other hand, when $\mathfrak {m} \leqslant 0$, and if instead of a random initial condition one chooses an additive space-time white noise, then equation (1.1) is a fundamental model in stochastic quantisation. In this case the invariant measure of the equation is a celebrated model in quantum field theory, and a recent proof of triviality in the critical dimension $d=4$ has been a breakthrough in the mathematical understanding of such measure [1]. At the same time, there is no result for the dynamics of the equation in $d =4$.

We will study (1.1) in a *weak coupling* regime. Namely, for $\varepsilon \in (0, \tfrac{1}{2})$^1^ and $p_{t}( x) = \tfrac{1}{2 \pi t} \exp \left( -\tfrac{|x|^2}{ 2 t} \right)$, we study the limiting behaviour of the solution $\mathcal {U}_{\varepsilon }$ to

1.3 $$\begin{aligned} \partial _t \mathcal {U}_\varepsilon = \frac{1}{2}\Delta \mathcal {U}_\varepsilon + \mathfrak {m}\, \mathcal {U}_{\varepsilon } - \frac{ 1}{\log { \tfrac{1}{\varepsilon }}} \mathcal {U}_\varepsilon ^{3}, \qquad \mathcal {U}_\varepsilon (0,\cdot )= \hat{\lambda }\, p_{\varepsilon ^{2}} \star \eta (\cdot ), \end{aligned}$$

where $\star$ denotes spatial convolution. The parameter $\hat{\lambda } > 0$ will be referred to as the *coupling constant*. By scaling, (1.3) is equivalent to the problem

1.4 $$\begin{aligned} \partial _t u_\varepsilon = \frac{1}{2}\Delta u_\varepsilon + \mathfrak {m}\, u_{\varepsilon } - u_\varepsilon ^{3}, \qquad u_\varepsilon (0,\cdot )= \eta _\varepsilon (\cdot ):= \frac{ \hat{\lambda }}{\sqrt{\log { \tfrac{1}{\varepsilon }}}} p_{\varepsilon ^{2}} \star \eta (\cdot ), \end{aligned}$$

and the solutions to (1.3) and (1.4) are related via $\mathcal {U}_\varepsilon (t,x)=\sqrt{\log \tfrac{1}{\varepsilon }} \,\, u_\varepsilon (t,x)$. We will also use the notation $\lambda ^2_\varepsilon := \hat{\lambda }^{2} \big (\log { \tfrac{1}{\varepsilon }}\big )^{-1}$. We remark that even though the nonlinearity is attenuated to zero, it still has a non-trivial effect in the limit. To make this point more precise let us, first, state our main result:

### Theorem 1.1

There exists a $\hat{\lambda }_{\text {fin}} \in (0, \infty )$ such that if $\mathfrak {m} \in {\textbf {R}}$ and $T, \hat{\lambda } \in (0, \infty )$ satisfy

1.5 $$\begin{aligned} \begin{aligned} \overline{\mathfrak {m}} \, T \leqslant \log { (\hat{\lambda }_{\text {fin}} / \hat{\lambda })}, \qquad \text {with} \qquad \overline{\mathfrak {m}} = \max \{ \mathfrak {m}\;, 0 \}, \end{aligned} \end{aligned}$$

and if $\sigma _{\hat{\lambda }} := \sigma ( \hat{\lambda }^{2}) := ( 1+\frac{3}{\pi }\hat{\lambda }^{2})^{- \frac{1}{2}}$, where $\zeta \mapsto \sigma (\zeta )$ is the solution to

1.6 $$\begin{aligned} \frac{ {\,\text {d}}}{{\,\text {d}}\zeta } \sigma = - \frac{3}{2 \pi } \sigma ^{3} \qquad \text {with} \qquad \sigma (0) = 1, \end{aligned}$$

then we have:

$$\begin{aligned} \begin{aligned} \lim _{\varepsilon \rightarrow 0} \mathbb {E}\left[ \big | \mathcal {U}_{\varepsilon }(t, x) - \hat{\lambda }\, \sigma _{ \hat{\lambda }} \,e^{\mathfrak {m} t} \, p_{t} \star \eta (x) \big |^{2}\right] = 0, \qquad \forall (t, x) \in (0, T] \times {\textbf {R}}^{2}. \end{aligned} \end{aligned}$$

Our result shows that the non-linearity is *marginally*^2^*relevant* as it affects the size of the limiting fluctuations $\hat{\lambda }\, \sigma _{ \hat{\lambda }}$, which are strictly weaker than the fluctuations of the limit when simply dropping the non-linearity in (1.3) (the solution to this linear problem reads as $\hat{\lambda } e^{\mathfrak {m} t} \, p_{t} \star \eta (x)$). The latter describes the limiting fluctuations of a *sub-critical* scaling of the initial condition, considered in the previous work by Hairer–Lê–Rosati [25], which studied the long-time behaviour of (1.4). Let us note that our requirement $\lambda< \hat{\lambda }_{\text {fin}} < \infty$ emerges as a technical constraint and we do not expect any actual phase transition. The restriction comes from the necessity to control certain series expansions (see in particular Propositions 3.1 and 3.4 as well as Remark 3.2). We conjecture that the result should extend to all $\hat{\lambda }, t > 0$. In particular, the extension to arbitrary large times, for $\hat{\lambda }$ small enough, should be a consequence of Theorem 1.1: For an arbitrarily small time $t_{0}>0$ the solution $\mathcal {U}_{ \varepsilon }(t_{0},\cdot )$ converges to a smooth function. From time $t_{0}$ onwards, the non–linearity in (1.3) should have no more effect, due to the vanishing coupling constant, and the dynamics will be governed by the linearised equation of (1.3). This would be especially interesting in relation to the study of the metastable behaviour of the Allen–Cahn equation (with $\mathfrak {m} > 0$) at large scales. However, this is beyond the scope of this paper and will be studied separately.

The understanding of stochastic PDEs (and related statistical mechanics models) at the critical dimension is only now starting to take shape. The only studied examples, so far, are two-dimensional stochastic heat equations (SHE) with multiplicative space-time white noise, the two-dimensional isotropic and anisotropic KPZ equation and the two-dimensional Burgers equation. For the linear SHE, a weak coupling regime was noted by Bertini–Cancrini [2] and explored by Caravenna–Sun–Zygouras [13]. Here the coupling constant which appears in the weak scaling plays a crucial role as a phase transition takes place at a precisely defined, critical value. Below this value, Gaussian fluctuations, similar in spirit to Theorem 1.1 emerge [13], while a limiting field, which is not Gaussian or an exponential of Gaussian, emerges at the critical value [15, 16]. A similar phase transition takes place for the two-dimensional isotropic KPZ equation [7, 14, 20] but, so far, only Gaussian fluctuations below the critical coupling value have been established [14, 20]. This result has been obtained via the use of the Cole-Hopf transformation, which relates the solution of the linear SHE to that of the KPZ equation. For the two-dimensional anisotropic KPZ and Burgers equation, the weak coupling limit has been studied in [8–10] and Gaussian fluctuations have been established, building crucially on the explicit Gaussian invariant measure available for that equation. We note that no phase transition takes place in this setting. Moreover, Dunlap–Gu [17] and Dunlap–Graham [18] have proposed another approach to weak coupling limits of the (nonlinear) stochastic heat equations through the study of forwards-backwards SDEs, while the linear SHE on a critical hierarchical lattice has been explored by Clark [11]. Finally, non-linear PDEs with random initial data have also been studied in the context of dispersive equations, see for example [4, 19, 32] and references therein.

Our approach in this work attempts to make a first step towards analysing singular SPDEs at the *critical* dimension via a systematic study of its Wild expansion, i.e. an expansion of the solution (in the spirit of a Picard iteration) in terms of iterated space-time stochastic integrals that are indexed by trees and live in certain Wiener chaoses. An approach of this sort has been successful in the study of *subcritical* SPDEs via the theory of regularity structures [23], thanks to the fact that one can restrict attention to a finite number of terms in the expansion, before exploiting sophisticated analytic solution theories. On the contrary, at the critical case *all* terms in the expansion contribute and their contribution needs to be accounted for.

In the case of (1.3) and (1.4), analysing the terms in the Wild expansion, we discover that the main contribution comes from certain projections of the Wild terms on the first Wiener chaos, see Proposition 3.3. Hence, the Gaussian limiting behaviour. An interesting structure emerges that clarifies the role of the ODE (1.6) as determining the limiting order of the fluctuations of the Gaussian field. Roughly speaking, the terms in the Wild expansion, which have the dominant contribution, appear in the limit as a Gaussian variable, multiplied by an iterated integral in time variables indexed by another tree structure. The new iterated integrals are recognised as terms in the celebrated Butcher series (or B-series) expansion of the ODE (1.6). Our approach has both an analytical and a combinatorial component. On the analytical side, we take advantage of the contractive properties of (1.4) to control the error of the truncated Wild series (see Proposition 3.1 and Remark 3.2). This analysis is inspired by the study of *finite* Wild expansions performed in [25]. However, in order to treat a diverging number of terms, it requires a new and deeper understanding of the interplay between the graphical properties of the trees, that index terms in the expansion, and their analytic contribution. Thus, we introduce a combinatorial component to perform a detailed analysis of Wiener chaos decompositions in terms of graph theoretical trees. Here we find that specific cycles appearing in contracted trees (dubbed $\text {v}$-cycles) play a fundamental rôle, and interesting links to permutation cycles and their statistics appear: see Sects. 4 and 5.

Let us next highlight a curious link of our main result to a mean-field equation of McKean-Vlasov type. More precisely, the limiting fluctuations of (1.3) appear to agree with the limiting fluctuations of the McKean-Vlasov equation

1.7 $$\begin{aligned} \begin{aligned} \partial _t \mathcal {V}_{ \varepsilon } = \frac{1}{2} \Delta \mathcal {V}_{ \varepsilon } +\mathfrak {m}\, \mathcal {V}_{ \varepsilon } - \frac{3 }{\log {\tfrac{1}{\varepsilon }}} \mathbb {E}\left[ \mathcal {V}^{2}_{\varepsilon }\right] \cdot \mathcal {V}_{ \varepsilon }, \qquad \mathcal {V}_{ \varepsilon }(0,\cdot ) = \hat{\lambda }\, p_{\varepsilon ^{2}} \star \eta ( \cdot ). \end{aligned} \end{aligned}$$

In particular, we have that

### Proposition 1.2

For any $\hat{\lambda } > 0$ there exists a unique solution $\mathcal {V}_{ \varepsilon }$ to (1.7), in the sense of Definition 6.1, which satisfies:

$$\begin{aligned} \begin{aligned} \lim _{\varepsilon \rightarrow 0} \mathbb {E}\left[ | \mathcal {V}_{\varepsilon }(t, x) - \hat{\lambda }\, \sigma _{ \hat{\lambda }} e^{\mathfrak {m} t} p_{t} \star \eta (x) |^{2}\right] = 0, \qquad \forall (t, x) \in (0, \infty ) \times {\textbf {R}}^{2}, \end{aligned} \end{aligned}$$

with $\sigma _{{\hat{\lambda }}}$ as in (1.6).

An emergence of an equation like (1.7) might appear plausible if one considers an ansatz where the leading order terms of the solution to (1.3) is Gaussian. In such a setting and if $\mathcal {U}_{\varepsilon }$ is Gaussian, then the projection on the first homogeneous Wiener chaos of $\mathcal {U}_{\varepsilon }^{3}$ is given by $3 \mathbb {E}[\mathcal {U}_{\varepsilon }^{2} ] \mathcal {U}_{\varepsilon }$, which agrees with the nonlinearity in (1.7). However, $\mathcal {U}_\varepsilon$, itself is far from Gaussian and it is also far from obvious, a priori, that its limit (1.3) is Gaussian. A deeper understanding of the relations between (1.3) and (1.7) is desirable.

### Remark 1.3

We close the introduction by observing that our results should hold also in any dimension $d \geqslant 3$ (and $\mathfrak {m}=0$ for simplicity). In this case, the white noise $\eta$ switches from being critical to super-critical, and we could have considered initial data of the form $u_{ \varepsilon } (0,x) =\varepsilon ^{ \frac{d}{2} -1} p_{\varepsilon ^{2}} \star \eta (x)$. Then, the super-critical equivalent of Theorem 1.1 should read

$$\begin{aligned} \begin{aligned} \lim _{\varepsilon \rightarrow 0} \mathbb {E}\Big [ \big | \varepsilon ^{ -\frac{d}{2} +1} u_{ \varepsilon }(t, x) - \hat{\lambda }\, \sigma _{ \hat{\lambda }}(d)\, p_{t} \star \eta (x) \big |^{2} \Big ] = 0, \qquad \forall (t,x) \in (0, \infty ) \times {\textbf {R}}^{d}\,, \end{aligned} \end{aligned}$$

with

$$\begin{aligned} \begin{aligned} \sigma _{\hat{\lambda }}(d) := \left( 1+ \left( \tfrac{d}{2} -1\right) ^{-1}\frac{6\hat{\lambda }^2}{(4 \pi )^{d/2}}\right) ^{ -\frac{1}{ 2}}\,, \end{aligned} \end{aligned}$$

for all $\hat{\lambda }>0$ small enough. The statement should follow almost verbatim from our arguments.

### Outline of the paper

The remainder of the paper is structured as follows. In Sect. 2 we give an introduction to rooted trees and their use in the analysis of ODEs and SPDEs. In Sect. 3, we present the main steps of the proof of Theorem 1.1 while assuming the paper’s key ingredient, Proposition 3.3. Section 4 introduces notation and estimates required, before we provide a proof of Proposition 3.3 in Sect. 5. In Sect. 6 we prove Proposition 1.2. Finally, we establish some technical results in the Appendix.

### Notation

Let ${\textbf {N}}= \{0,1,2,\ldots \}$. We denote with $P_t= \exp \left( \frac{t}{2}\Delta \right)$ the heat semigroup on ${\textbf {R}}^2$:

$$\begin{aligned} \begin{aligned} P_t \varphi (x) = p_t \star \varphi (x), \quad p_t (x) = \frac{1}{2 \pi t} \exp \left( -\frac{|x|^2}{2 t} \right) \mathbb {1}_{[0, \infty )}(t), \quad \forall (t, x) \in {\textbf {R}}\times {\textbf {R}}^{2}, \end{aligned} \end{aligned}$$

where $\star$ denotes spatial convolution. Similarly, we define the heat semigroup with mass $\mathfrak {m} \in {\textbf {R}}$ by $P^{(\mathfrak {m})}_{t} = e^{\mathfrak {m}\, t} P_{t}$ and associated kernel $p^{(\mathfrak {m})}_{t}(x) = e^{\mathfrak {m}\, t}p_{t}(x)$. Here we allow the semigroup to be defined for any Schwartz distribution $\varphi$ on ${\textbf {R}}^{2}$: if such $\varphi$ is not locally integrable the integral should be interpreted in the usual generalised sense. We will abuse notation and sometimes write singletons of the form $\{a\}$ simply as *a*. Thus, $A \setminus a := A \setminus \{a\}$, for some set *A*.

## Trees, wild expansion and Butcher series

The linchpin of our argument is a precise control on the Wild expansion of the solution $u_{\varepsilon }$ to the equation (1.4). Wild expansions were popularised in the context of stochastic PDEs by Hairer’s seminal work [22], and are originally attributed to the work of Wild [35]. A Wild expansion is an expansion of a solution to a parabolic PDE or an ODE in terms of iterated integrals. The terms in such an expansion are naturally indexed by rooted trees, which in our setting are associated—similarly to Feynman diagrams—to integrals involving the heat kernel and the correlation function of the noise $\eta _{\varepsilon }$. Wild expansions are also naturally linked to Butcher series’—see for example [21] and the many references therein—which allow for a tidy bookkeeping of the coefficients appearing in the Wild expansion of a solution to an ODE. This section is devoted to establish all such connections rigorously, and in a manner that will be useful to our analysis.

### Finite rooted trees

We start by introducing basic concepts concerning trees. We will work with finite, rooted trees. A tree $\tau$ is a connected, undirected planar graph that contains no cycle. We denote by $\mathcal {V}(\tau )$ the set of vertices of a tree $\tau$ and by $\mathcal {E}(\tau )$ the set of its edges. A finite rooted tree is a tree with a finite number of vertices, and one particular vertex $\mathfrak {o} \in \mathcal {V}(\tau )$ singled out as the **root**. Rooted trees induce a partial order on the set of vertices $\mathcal {V}(\tau )$, by writing $v \prec w$ if the unique path from *w* to the root $\mathfrak {o}$ passes through *v*, for any pair $v, w \in \mathcal {V}(\tau )$ with $v \ne w$. In particular, if $v \prec w$ we say that *w* is a **descendant** of *v* or that *v* is an **ancestor** of *w*, and we say that *v* is a **leaf** if it has no descendants. The closest ancestor $v \in \mathcal {V}( \tau )$ of $w \in \mathcal {V}( \tau ) \setminus \mathfrak {o}$ is called **parent** of *w*, we write $\mathfrak {p}(w)=v$. The set of leaves of a tree $\tau$ will be denoted by $\mathcal {L}(\tau )$ and we define $\ell (\tau ):=|\mathcal {L}(\tau )|$ the total number of leaves in a tree. The collection of vertices of a tree which are not leaves will be called **inner vertices**, it will be denoted by $\mathcal {I}(\tau )$ and its cardinality is defined to be $i(\tau )$. An exception to this convention will be made when the root is the only vertex of the tree, in which case it will be considered as a leaf and thus *not* an inner vertex. The cardinality of all the vertices of a tree $\tau$ will be denoted by $|\tau |$ and we have that $|\tau |=\ell (\tau )+i(\tau )$. Finally, we call the **degree** of a vertex the number of incident edges.

As a convention, we draw trees (from now on always finite and rooted) growing upwards, out of the root of the tree, which is placed at its bottom. For example, the following are trees with inner vertices coloured white (and circled red) and leaves coloured black:2.1Here ${\textbf {1}}$ is the empty tree, which is different from the single root  . Next, we will be working with *unordered* trees, which means, for example, that the three trees below will be considered to be identical:We will denote by $\mathcal {T}$ the set of all finite, unordered, rooted trees. By convention $\mathcal {T}$ contains also the empty tree ${\textbf {1}}$.

In our setting, large trees arise naturally from smaller ones by combining them, as we will now explain. Suppose that $\tau _1,...,\tau _n\in \mathcal {T}$ are given. Then we define the grafted tree $\tau =[\tau _1 \cdots \tau _n]$ which is built by connecting the roots of the trees $\tau _1,...,\tau _n\in \mathcal {T}$, by means of new edges, to a new common vertex, which acts as the root of the new tree $\tau$. The following graphical representation is perhaps the best explanation of this construction:2.2Here are some examples of graftings of trees:We observe that the empty tree ${\textbf {1}}$ can be ignored in a grafting (unless it is the only tree): $[{\textbf {1}}\ \tau _{1} \cdots \tau _{n} ]= [\tau _{1} \cdots \tau _{n}]$. Next, one obtains the space of finite, unordered rooted trees from the space of finite rooted trees by quotienting through the equivalence relation that identifies

$$\begin{aligned} {[}\tau _1 \cdots \tau _n] \equiv [\tau _{\upsigma (1)} \cdots \tau _{\upsigma (n)}] \end{aligned}$$

for every $\upsigma \in S_n$ (the group of permutations on *n* indices) and any choice of $n \in {\textbf {N}}$ and trees $\tau _{1}, \ldots , \tau _{n}$ and the same for any subtree of a given tree.

In particular, due to the cubic nonlinearity that characterises the Allen–Cahn equation, we will be dealing with **sub-ternary** trees:

$$\begin{aligned} \mathcal {T}_{\leqslant 3} := \{ \tau \in \mathcal {T}\,:\, \text {any inner vertex in } \tau \text { has at most }3\text { descendants} \}\,, \end{aligned}$$

and its subset $\mathcal {T}_3$ of **ternary** trees:

$$\begin{aligned} \mathcal {T}_{3} := \{ \tau \in \mathcal {T}\setminus \{ {\textbf {1}} \}\,:\, \text {any inner vertex in }\tau \text { has exactly }3\text { descendants} \}\,. \end{aligned}$$

For example, $\mathcal {T}_{\leqslant 3}$ contains all the trees appearing in (2.1), and the second, third and fourth trees in (2.1) additionally lie in $\mathcal {T}_3$. We work with the conventions that the empty tree belongs to $\mathcal {T}$ and $\mathcal {T}_{\leqslant 3}$, but *not* $\mathcal {T}_{3}$ and that the “single vertex” tree  belongs to $\mathcal {T}_{3}$ (here by convention the root counts as a leaf and not as an inner vertex). Note that the last tree in (2.1) is not ternary, as its root has degree one.

It will be convenient to introduce some terminology for subtrees of sub-ternary trees. We will call the tree  a **trident**,  a **cherry** and  a **lollipop**. Further, when these trees are embedded into larger trees, we will call the root vertices of these components (marked in red white here) respectively the **basis of the trident**, **basis of the cherry** or **basis of the lollipop**. Finally, we note that for ternary trees $\tau \in \mathcal {T}_3$, the number of leaves $\ell (\tau )$ and the number of inner vertices $i(\tau )$ satisfy the relation $\ell (\tau )=2i (\tau ) +1$.

We close this subsection by introducing several important quantities:

**1.**: **Symmetry factors.** For any $\tau \in \mathcal {T}$ we write $s(\tau ) \in {\textbf {N}}$ for the *symmetry factor* associated to the tree. This amounts to the cardinality of the symmetry group associated with ${\tau }$. More precisely, if we assign a label to each vertex of the tree $\tau$, then $s(\tau )$ is the number of permutations of the labels that leave the structure of rooted unordered tree invariant. It is given by the following recursive formula. First, set . Then, if $\tau$ is of the form ${\tau } = [(\tau _1)^{k_1} \cdots (\tau _n)^{k_n}]$ for pairwise distinct ${\tau _i}$’s, each one appearing $k_i$ times and with $\tau _{i} \ne {\textbf {1}}$, for $i=1,...,n$, then
  2.3 $$\begin{aligned} s({\tau }) = \prod _{i=1}^n k_i!\; s({\tau _i})^{k_i}\,. \end{aligned}$$
  Since any rooted tree can be constructed by grafting together strictly smaller trees, the above defines the symmetry factor for all rooted trees.
**2.**: **Tree factorials.** Similarly we define the tree factorial $\tau ! \in {\textbf {N}}$ for any $\tau \in \mathcal {T}$. For the empty tree ${\textbf {1}}$ we define ${\textbf {1}}!=1$ and, iteratively, for a tree ${\tau }= [\tau _1 \cdots \tau _n]$ we define $\tau !$ by
  $$\begin{aligned} \tau ! = | \tau | \, \tau _1 ! \cdots \tau _n ! \, , \end{aligned}$$
  where $|{\tau }|=1+|\tau _1| + \cdots |\tau _n|$ is the total number of vertices of the tree $\tau$. We observe that the tree factorial of a linear tree over *n* vertices (that is, the tree over *n* vertices in which every inner vertex has exactly one descendant), is equal to *n*!. Thus, the notion of tree factorial generalises the usual notion of factorial.
**3.**: **Tree differentials.** For an analytic function $h :{\textbf {R}}\rightarrow {\textbf {R}}$, we define recursively its *tree differential* (sometimes called *elementary differential*) $h^{(\tau )}: {\textbf {R}}\rightarrow {\textbf {R}}$ as follows: For all $y\in {\textbf {R}}$, we define $h^{( {\textbf {1}})}(y) = y$ for the empty tree ${\textbf {1}}$ and we set  for the single vertex tree . Then, for an arbitrary tree  such that ${\tau } = [\tau _1 \cdots \tau _n]$, $\tau _{i} \ne {\textbf {1}}$, we define inductively
  2.4 $$\begin{aligned} \begin{aligned} h^{ ( \tau )} (y) = h^{(n)} (y)\, \prod _{j=1}^n h^{( \tau _{j})} (y), \quad \text {for all} \quad y \in {\textbf {R}}, \end{aligned} \end{aligned}$$
  where $h^{(n)}$ is the usual $n^{th}$ derivative of *h*.

### Butcher series

In this section we will review how trees are used to index series expansions of solutions to ordinary differential equations. In particular, the kind of expansion that we are interested in goes under the name of *Butcher series* (or *B-series* for short) [5, 26]. As we have already mentioned, the structure of such series expansions in combination with the structure of our Wild expansion plays an important role in our analysis. In particular, this structure lies behind the identification of the limiting fluctuation strength $\sigma _{ \hat{\lambda }}$ in Theorem 1.1. To start our brief discussion on Butcher series, consider an analytic function $h:{\textbf {R}}\rightarrow {\textbf {R}}$ and the differential equation

$$\begin{aligned} \frac{ {\,\text {d}}y}{{\,\text {d}}\zeta }=h(y)\,, \quad \forall \zeta >0 \quad \text {and} \quad y(0)=y_0 \in {\textbf {R}}\,. \end{aligned}$$

Then the solution $y(\zeta )$ can be expressed, locally around $\zeta =0$, through the following series:

2.5 $$\begin{aligned} y(\zeta ) = \sum _{{\tau } \in \mathcal {T}} \frac{h^{( \tau )} (y_0)}{{\tau }!\, s({\tau })} \zeta ^{|{\tau }|} =: B_h(\zeta , y_{0}) \,, \qquad \forall \zeta \in [0, \zeta _{\star } ) \;, \end{aligned}$$

where $\zeta _{\star } > 0$ depends on *h* and $y_{0}$. The sum runs over all rooted, unordered trees (including the empty tree ${\textbf {1}}$) and $s (\tau ),\tau !$ and $h^{( \tau )}$ have been defined in Sect. 2.1 above. For details on this derivation we refer to [5, 6, 26], see also [21, Theorem 5.1]. In any case, at the heart of (2.5) lies the identity (see for example [6, Theorem 311C])

2.6 $$\begin{aligned} \begin{aligned} \frac{y^{(n)}(0)}{n!} = \sum _{ \begin{array}{c} \tau \in \mathcal {T}\\ | \tau | =n \end{array}} \frac{h^{( \tau )}(y_{0})}{ \tau !\, s ( \tau )}\,, \end{aligned} \end{aligned}$$

which allows to express the solution of the ODE in terms of the Butcher series, whenever its Taylor series centered at zero converges absolutely. In this work, the following ODEs are of particular relevance:

2.7 $$\begin{aligned} \begin{aligned} {\left\{ \begin{array}{ll} \dot{y}&{}=-y^3\\ y (0)&{}=1 \end{array}\right. } \qquad \text { and} \qquad {\left\{ \begin{array}{ll} \dot{\overline{y}}&{}=\overline{y}^3\\ \overline{y}(0)&{}=1 \end{array}\right. } \,, \end{aligned} \end{aligned}$$

both of which admit explicit solutions $y(\zeta )=(1+2\zeta )^{-1/2}$ and $\overline{y}(\zeta )=(1-2\zeta )^{-1/2}$, respectively. Observe that both solutions are real analytic at 0 with radius of convergence $\tfrac{1}{2}$, so that (2.5) holds with $\zeta _{\star }= \tfrac{1}{2}$. Strikingly $\overline{y}$ explodes at $\zeta = 1/2$, whereas *y* is defined for all times. Our approach to treating the coefficients of the Butcher series does not allow to distinguish the behaviour of the solution *y* from that of $\overline{y}$ and leads ultimately to one of the requirements in Theorem 1.1 for the coupling constant $\hat{\lambda }$ to be sufficiently small. Overcoming this issue would require to take into account the sign of the terms in the Butcher series (or avoid the series entirely), and this lies beyond the reach of our current proofs.

### Wild expansion

In this subsection we establish some basic properties concerning the Wild expansion of the solution $u_{\varepsilon }$ to (1.4). By convolving with the heat kernel, we can explain the heuristics that lead formally to the derivation of the Wild expansion to (1.4). We write the solution of equation (1.4) in its mild formulation

2.8 $$\begin{aligned} u_\varepsilon (t,x)&= P_t^{(\mathfrak {m})} \eta _\varepsilon (x) - \int _0^t \Big (P^{(\mathfrak {m})}_{t-s} u_\varepsilon ^3(s,\cdot ) \Big ) (x)\,{\,\text {d}}s \nonumber \\&= \int _{{\textbf {R}}^2} p^{(\mathfrak {m})}_{t}(x-y) \eta _\varepsilon (y) {\,\text {d}}y - \int _0^t\int _{{\textbf {R}}^2} p^{(\mathfrak {m})}_{t-s}(x-y) u_\varepsilon ^3(s,y) {\,\text {d}}s \, {\,\text {d}}y \nonumber \\&= \int _0^t\int _{{\textbf {R}}^2} p^{(\mathfrak {m})}_{t-s}(x-y) \eta _\varepsilon (y) \delta _0(s) {\,\text {d}}s {\,\text {d}}y - \int _0^t\int _{{\textbf {R}}^2} p^{(\mathfrak {m})}_{t-s}(x-y) u_\varepsilon ^3(s,y) {\,\text {d}}s {\,\text {d}}y, \end{aligned}$$

where in the last line we rewrote the first integral using a delta function at time $s=0$. We now introduce our first diagrammatic notation:2.9with the right-hand side interpreted in the Itô sense. Here we associate to the lollipop a random function in the following way. We assign the time-space variable (*t*, *x*) to the root, the time-space variable (*s*, *y*) as well as the weight $\delta _0(s) \, \eta _\varepsilon (y)$ to the leaf and the kernel $p_{t-s}^{(\mathfrak {m})}(x-y)$ to the connecting edge. Finally, we integrate over the variables associated to all vertices except the root. In other words, the edge represents a time-space convolution between the heat kernel and the weight of the leaf, evaluated at the time-space variables assigned to the root. Therefore, we can rewrite (2.8) as follows:2.10Writing explicitly the arguments of the functions appearing in (2.10) can be cumbersome, so to shorten the notation we will equivalently writewhere $P^{(\mathfrak {m})} *\varphi = \int _{{\textbf {R}}\times {\textbf {R}}^{2}} p^{(\mathfrak {m})}_{t-s}(x-y) \varphi _{s} (y) {\,\text {d}}y {\,\text {d}}s$ denotes space-time convolution with the kernel $p^{(\mathfrak {m})}$. Now, we can iterate this description of $u_{\varepsilon }$ by inserting the identity for $u_\varepsilon$ in (2.10) into the right-hand side of the expression itself:2.11Expanding the cube on the right-hand side above, we find the expression2.12In order to motivate the representations we are after, let us just focus on the second term of the above expression. We use the following representation of the cubic powerwhere we have glued together three lollipops at a common root, thus forming a trident whose basis is associated to time-space coordinates (*t*, *x*). We then introduce the planted trident:which allows us to rewrite (2.12) in terms of2.13We observe that, crucially, the first two terms are rather explicit: they live in a finite inhomogeneous Wiener chaos and we are able to control them with tools from stochastic analysis. Of course, we can continue this expansion at will and iterating the procedure above we obtain formally an expression for $u_{\varepsilon }$ that we call Wild expansion. The elements of this expansion are given in the next definition.

#### Definition 2.1

To any tree $\tau \in \mathcal {T}_{3}$ and $\varepsilon \in (0, \tfrac{1}{2})$ we associate a random function $(t, x) \mapsto X_\varepsilon ^{ \tau } (t, x)$ for $(t, x) \in (0, \infty ) \times {\textbf {R}}^{2}$ as follows. For  we setThen, iteratively, assuming we have defined $X_{\varepsilon }^{\tau _1}, X_{\varepsilon }^{\tau _2}, X_{\varepsilon }^{\tau _3}$ for trees $\tau _1,\tau _2,\tau _3 \in \mathcal {T}_3$, we define $X_\varepsilon ^\tau$ for the tree $\tau =[\tau _1 \ \tau _2 \ \tau _3] \in \mathcal {T}_3$ as

$$\begin{aligned} X_\varepsilon ^{ \tau } (t, x)&:= -a( \tau ) P^{(\mathfrak {m})} *\big (\, X^{\tau _1}_{\varepsilon } X^{\tau _2}_{\varepsilon }X^{ \tau _3}_{\varepsilon } \, \big ) \,, \end{aligned}$$

where $a( \tau )$ is the combinatorial factor

2.14 $$\begin{aligned} a(\tau ) = \ {\left\{ \begin{array}{ll} 1\;,\qquad \text {if} \quad \tau _1=\tau _2=\tau _3\;,\\ 3\;,\qquad \text {if exactly two of the trees }\tau _1,\tau _2,\tau _3 \text {coincide}\;, \\ 6\;,\qquad \text {if all trees }\tau _1,\tau _2,\tau _3\text { are distinct}\;, \end{array}\right. } \end{aligned}$$

which appears because we are considering unordered trees. The Wild expansion associated to the Allen–Cahn equation (1.4) is then defined as the (formal) series $\sum _{\tau \in \mathcal {T}_3} X^\tau _\varepsilon .$

Now we will connect the terms $X^{\tau }_{\varepsilon }$ from Definition 2.1 to explicit stochastic integrals. This will follow in two steps: first we generalize the diagrammatic representation that we have started to introduce in (2.13). Then we show (see Lemma 2.4 below) how an iterated stochastic integral relates, up to an appropriate combinatorial factor, to the associated element of the Wild expansion.

To this end, for any tree $\tau \in \mathcal {T}_3$ with vertices $\mathcal {V}( \tau )$, partitioned into leaves $\mathcal {L}(\tau )$, inner vertices $\mathcal {I}(\tau )$, and edges $\mathcal {E}(\tau )$, we associate the (inhomogeneous) Wiener integral

2.15 $$\begin{aligned}{}[\tau ]_\varepsilon (t,x)= \int _{D_{t}^{\mathcal {V}(\tau )}} \prod _{u \in \mathcal {V}( \tau )} p_{s_{\mathfrak {p}(u)} - s_{u}}^{(\mathfrak {m})}(y_{\mathfrak {p}(u)} - y_{u}){\,\text {d}}s_{\mathcal {I}(\tau )} {\,\text {d}}y_{\mathcal {I}(\tau )} \prod _{v \in \mathcal {L}(\tau )} \delta _{0}({\,\text {d}}s_v)\eta _\varepsilon ( {\,\text {d}}y_v),\nonumber \\ \end{aligned}$$

where $[ \tau ]$ denotes the planted version of $\tau$, cf. (2.2). Here

2.16 $$\begin{aligned} \begin{aligned} D_{t} := [0, t] \times {\textbf {R}}^{2} \end{aligned} \end{aligned}$$

and $D_{t}^{\mathcal {V}(\tau )}$ is the Cartesian product of $D_{t}$ over the index set $\mathcal {V}( \tau )$. Moreover, we recall that $\mathfrak {p}(u)$ denotes the unique parent of *u* with $\mathfrak {p}(\mathfrak {o}_{ \tau }) = \mathfrak {o}_{[ \tau ]}$ the vertex associated to the time-space point (*t*, *x*), and $\mathfrak {o}_{[ \tau ]}$ being the root of the planted tree $[\tau ]$. The integral (2.15) does *not* neglect integration over any diagonals, hence, it lies in an inhomogeneous Wiener chaos, see also (4.6) below. Note that in the integral (2.15) we are *not* integrating over the root variable of the tree $[\tau ]$ (which is assigned to the point (*t*, *x*) ), but integrate instead over all other vertices of $[\tau ]$, i.e. all vertices of $\tau$. Further, observe that with this definition, by construction, if $[\tau ] = [ [\tau _{1}] \, [\tau _{2}] \, [\tau _{3}]]$, then

2.17 $$\begin{aligned} \begin{aligned} {[} \tau ]_{ \varepsilon } =P^{(\mathfrak {m})} *( [ \tau _{1}]_{ \varepsilon } [ \tau _{2}]_{ \varepsilon } [ \tau _{3}]_{ \varepsilon }). \end{aligned} \end{aligned}$$

Next, we remark that in general, a term $X^{\tau }_{\varepsilon }$ of the Wild expansion is related to the Wiener integral represented by the associated planted tree $[\tau ]_{\varepsilon }$ via a combinatorial factor $c_\tau \in {\textbf {N}}$ as $X_\varepsilon ^{ \tau } =c_{\tau }\cdot [\tau ]_{\varepsilon }$. With the formulation above we have for example:The factors $c_\tau$ appear because we are considering unordered trees (and ultimately because of the commutative property of the product). It will be important to obtain a precise expression for $c_{\tau }$, and this is the objective of the remainder of this section. Our eventual expression for $c_{\tau }$ contains tree derivatives with respect to a “trimmed” version of $\tau$. Therefore, we start by introducing a “trimming” operator on trees.

#### Definition 2.2

We call the map2.18the *trimming* operator, where $\mathscr {T}(\tau )$ is the tree that is spanned by the inner vertices of $\tau$, i.e. $\mathscr {T}$ “cuts off” all the leaves and the edges attached to them.

To lighten later notation, we have also used the chromatic notation by which , for exampleThe reader should have the following pictorial description of $\mathscr {T}$ in mind2.19where we again coloured leaves black in the last expression according to the chosen convention. A first result regarding the trimming operation guarantees that it is a bijection between finite families of ternary and sub-ternary trees of the following form, for arbitrary $N \in {\textbf {N}}$

2.20 $$\begin{aligned} \begin{aligned} \mathcal {T}_3^N&= \{ \tau \in \mathcal {T}_{3} \; :\; i(\tau ) \leqslant N \} \subseteq \mathcal {T}_{3}\,, \\ \mathcal {T}_{\leqslant 3}^{N}&= \{ \tau \in \mathcal {T}_{\leqslant 3} \; :\; | \tau | \leqslant N \} \subseteq \mathcal {T}_{\leqslant 3} \,. \end{aligned} \end{aligned}$$

The next lemma summarises this and other properties of $\mathscr {T}$.

#### Lemma 2.3

The following hold.

(i) The map $\mathscr {T}$ is a bijection from $\mathcal {T}_3^N$ to $\mathcal {T}^{N}_{\leqslant 3}$, for every $N\in {\textbf {N}}$.
(ii) Let  and $\tau _{1}, \tau _{2} , \tau _{3} \in \mathcal {T}_{3}$ such that $\tau = [ \tau _{1}\ \tau _{2}\ \tau _{3}]$, then  In other words, trimming via $\mathscr {T}$ and grafting via $[ \ldots ]$ commute.

#### Proof

By definition of $\mathcal {T}_{3}^N$, its image under $\mathscr {T}$ is a subset of $\mathcal {T}^{N}_{\leqslant 3}$. On the other hand, for any $\tau \in \mathcal {T}_{\leqslant 3}^{N}$ we can construct a $\sigma \in \mathcal {T}_{3}^N$ such that $\mathscr {T}(\sigma ) = \tau$ as follows. To each vertex that has $3 - k$ descendants, for $k \in \{ 1,2,3 \}$, we append exactly *k* lollipops , so that in the new tree that vertex has exactly three outgoing edges. The constructed tree $\sigma$ lies in $\mathcal {T}_3^N$, since $i ( \sigma ) = | \tau | \leqslant N$ and every inner vertex of $\sigma$ has exactly three descendants. Moreover, it satisfies $\mathscr {T}(\sigma )=\tau$. This concludes the proof of the first part of the statement.

In order to prove (ii), let  and $\tau _{1}, \tau _{2}, \tau _{3} \in \mathcal {T}_{3}$ such that $\tau = [ \tau _{1}\ \tau _{2}\ \tau _{3}]$. Now, because $\mathscr {T}$ does not act on the root of $\tau$, cf. Definition 2.2, we necessarily haveIn order to avoid confusion, let us discuss explicitly the case where  and thus , for some $i \in \{1,2,3\}$. Without loss of generality let us assume that , as in the example (2.19) displayed above. Then, by convention of $[\ldots ]$, we haveThis identity propagates to  if additionally . Moreover, in the most extreme case , this reduces further to . $\square$

The following result establishes the link between the Butcher series and the Wild expansion, according to our definitions.

#### Lemma 2.4

The following identity holds for $h(y) = - y^{3}$ and any $\tau \in \mathcal {T}_{3}$:with the symmetry factor  and elementary differential  defined in (2.3) and (2.4), respectively.

#### Proof

The statement is true for  since . Now we proceed by induction. Assume that the statement is true for all trees $\tau \in \mathcal {T}_3$ with $|\tau |\leqslant n$, for some given $n \in {\textbf {N}}$, and let $\tau \in \mathcal {T}_3$ be a tree with $n+1$ vertices such that $\tau = [\tau _1\ \tau _2\ \tau _3]$, $\tau _{i} \in \mathcal {T}_{3}$. Furthermore, observe that the combinatorial factor $a(\tau )$ appearing in Definition 2.1 can be expressed as follows:

$$\begin{aligned} \begin{aligned} a(\tau ) = 3! \frac{s(\tau _1) s(\tau _2) s(\tau _3)}{s(\tau )} \;, \end{aligned} \end{aligned}$$

with the symmetry factor $s(\tau )$ defined in (2.3). Or, in other words

2.21 $$\begin{aligned} \begin{aligned} \frac{s(\tau _1) s(\tau _2) s(\tau _3)}{s(\tau )} =\ {\left\{ \begin{array}{ll} \tfrac{1}{6},\ \ \text {if} \ \ \tau _1=\tau _2=\tau _3 ,\\ \tfrac{1}{2},\ \ \text {if exactly two of the trees } \tau _1,\tau _2,\tau _3 \text { coincide}, \\ 1,\ \ \text {if all trees }\tau _1,\tau _2,\tau _3\text { are distinct}. \end{array}\right. } \end{aligned} \end{aligned}$$

Therefore, from the definition of the terms of the Wild expansion, see again Definition 2.1, we have2.22by our induction hypothesis, and by (2.17). Now, let $k \in \{0,1,2,3\}$ be the number of trees $\tau _{i}$ satisfying . As we consider unordered trees, we can writewithout loss of generality. Thussince  and by using the fact thatwhich is a direct consequence of the symmetry factor’s definition (2.3). Next, due to commutativity of the trimming and grafting cf. Lemma 2.3(ii), we have . In particular this implies, using Lemma 2.3(i), that if *j* of the $\tau _i$’s agree, then also *j* of the ’s agree. Therefore, since as in (2.21), the ratio

$$\begin{aligned} \begin{aligned} \frac{ s(\tau _1) \cdots s(\tau _{k})}{s([\tau _{1}\ \cdots \ \tau _{k}])} \end{aligned} \end{aligned}$$

only depends on the number of identical subtrees, we obtainOverall, (2.22) can therefore be rewritten aswhere we used the definition of the elementary differential (2.4) together with the fact that . This concludes the proof. $\square$

With this, we have introduced all the elements which allow us to discuss the proof of Theorem 1.1, without entering into technical details. These are deferred to later sections and require the introduction of additional tools.

## Outline and proof of main result

The first step towards the proof of Theorem 1.1 is the analysis of single terms in our Wild expansion. The key result in this direction (Proposition 3.3) is presented in the upcoming Sect. 3.1. The proof of Theorem 1.1 is then carried out in Sect. 3.2.

### From Wild expansion to single-tree estimates

Formally, the solution of (1.4) can be represented in terms of the Wild series expansion as

$$\begin{aligned} \begin{aligned} u_\varepsilon&= \sum _{\tau \in \mathcal {T}_3} X^\tau _\varepsilon = \sum _{\tau \in \mathcal {T}_3^N } X^\tau _\varepsilon + \sum _{\tau \in \mathcal {T}_{3} \setminus \mathcal {T}_3^N } X^\tau _\varepsilon =: u_\varepsilon ^N + \big ( u_\varepsilon -u_\varepsilon ^N\big )\,, \end{aligned} \end{aligned}$$

where $u_\varepsilon ^N$ is defined to be the series $\sum _{\tau \in \mathcal {T}_3^N } X^\tau _\varepsilon$ truncated at level $N \in {\textbf {N}}$. Unlike the full Wild expansion, $u_{\varepsilon }^{N}$ is well defined as it is a finite sum. The structure of the Allen–Cahn equation and in particular the fact that the non-linearity $-u^3$ is monotone in *u*, allows to circumvent a direct treatment of the infinite part of the series. More precisely, we have that $u_\varepsilon ^N$ solves

3.1 $$\begin{aligned} \partial _t u_\varepsilon ^{N} = \frac{1}{2} \Delta u_\varepsilon ^{N}+ \mathfrak {m} \, u_{ \varepsilon }^{N}- (u_\varepsilon ^{N})^3 + R_\varepsilon ^{N}\,, \quad u_\varepsilon ^{N}(0,\cdot ) = \eta _{\varepsilon } ( \cdot )\,, \end{aligned}$$

where the error term $R_\varepsilon ^{ N}$ depends only on trees at the “boundary” of $\mathcal {T}^{N}_{3}$:3.2Here we used the fact that any tree in  can be written recursively as $\tau =[\tau _1 \, \tau _2\, \tau _3]$, for some smaller $\tau _1, \tau _2,\tau _3 \in \mathcal {T}_3$, hence,

$$\begin{aligned} \Big (\sum _{\tau \in \mathcal {T}_3^N} X_\varepsilon ^{\tau } \Big )^3 = \sum _{\begin{array}{c} \tau \in \mathcal {T}_3^N\\ \tau = [\tau _1\, \tau _2\, \tau _3] \end{array}} a(\tau )\, X_\varepsilon ^{\tau _1}X_\varepsilon ^{\tau _2}X_\varepsilon ^{\tau _3} +R_\varepsilon ^{N}\,. \end{aligned}$$

Utilising a maximum principle in combination with more structural estimates, which we will describe in detail below, we are able to control the error of the approximation as follows.

#### Proposition 3.1

Let $\hat{\lambda }>0$ and $T>0$ satisfy

3.3 $$\begin{aligned} \begin{aligned} \hat{\lambda } e^{ \overline{\mathfrak {m}} \, T} < \frac{1}{10\sqrt{C}} \;, \end{aligned} \end{aligned}$$

with $$ \overline{\mathfrak {m}}= \max \{ \mathfrak {m}\;, 0 \}$$ and

3.4 $$\begin{aligned} \begin{aligned} C:= \frac{6e^{ 2 + 2 \pi }}{\pi } \,. \end{aligned} \end{aligned}$$

Then uniformly over all $(t,x) \in (0,T]\times {\textbf {R}}^{2}$, $\varepsilon \in (0, \tfrac{1}{T} \wedge \tfrac{1}{2})$ and $N \leqslant \lfloor \log { \tfrac{1}{\varepsilon }} \rfloor$

3.5 $$\begin{aligned} \sqrt{\log \tfrac{1}{\varepsilon }} \left\| \, u_\varepsilon ^{ N}(t,x) - u_{\varepsilon }(t,x) \, \right\| _{L^{2}(\mathbb {P})} \leqslant \frac{ C_{0}}{\log { \frac{1}{\varepsilon }}} \frac{ \big ( \sqrt{C}\hat{\lambda } e^{ \overline{\mathfrak {m}}\, t}\big )^{N}}{\varepsilon } \,, \end{aligned}$$

with $C_{0}= C_{0}(T, \mathfrak {m}, \hat{\lambda }) \in (0, \infty )$ the constant defined in (3.21).

The proof of this proposition is deferred to Sect. 3.2. In order for this estimate to help us prove Theorem 1.1, we would like the right-hand side of (3.5) to vanish as $\varepsilon \rightarrow 0$. This forces us to choose a cut-off level $N= N_{ \varepsilon }$ that grows to infinity as $\varepsilon \rightarrow 0$, and a suitably small coupling constant $\hat{\lambda }$ satisfying (3.3). In particular, we fix the cut-off level $N_{\varepsilon }$ given by

3.6 $$\begin{aligned} N_\varepsilon = \left\lfloor \log \tfrac{1}{\varepsilon } \right\rfloor \,. \end{aligned}$$

We note that this estimate runs along the same lines as [25, Proposition 3.15], with the fundamental difference that we need to push the estimate here to be uniform over a growing *N*, more precisely $N \leqslant \lfloor \log { \tfrac{1}{\varepsilon } } \rfloor$. Indeed in [25], for $\eta _\varepsilon$ scaled (in the two-dimensional setting) as $\varepsilon ^{1-\alpha } p_\varepsilon \star \eta$ with $\alpha \in (0,1)$, the bound that one obtains is of the form:

$$\begin{aligned} \begin{aligned} \left\| \, u_\varepsilon ^{ N}(t,x) - u_{\varepsilon }(t,x) \, \right\| _{L^{2}(\mathbb {P})} \leqslant C(N, \mathfrak {m}, t) \varepsilon ^{2 - 3\alpha } \left( e^{\mathfrak {m} t} \varepsilon ^{1- \alpha } (1+\log {(t \varepsilon ^{-2})}) \right) ^{N}\,, \end{aligned} \end{aligned}$$

for some constant *C*. The parameter $\alpha \in (0, 1)$ modulates the sub-critical level of the noise. We see here that if $\alpha < 1$, then in order to make the left-hand side small, it suffices to choose *N* finite, but sufficiently large. Instead, in our setting which corresponds to $\alpha =1$, this bound degenerates and is replaced by (3.5). Having a control on the error, then, leads to the need of a growing choice of $N_\varepsilon$. We collect the essential elements of this discussion in the following remark.

#### Remark 3.2

The estimate in Proposition 3.1 is crucial to understand our approach. First, this bound forces us to control the Wild expansion up to the (diverging) level $N_{\varepsilon } = \lfloor \log { \tfrac{1}{\varepsilon } } \rfloor$, uniformly over $\varepsilon$. It is for this reason that we require the precise estimates on the stochastic integrals associated to trees up to size $| \tau | \leqslant N_{\varepsilon }$, which are at the heart of our work. Second, this bound imposes us to work with a *small* coupling constant $\hat{\lambda }$ and small times (if $\mathfrak {m} > 0$), although we expect our main result to hold for all times and coupling constants.

Given the error estimate in Proposition 3.1 above, the next task is to identify the convergence of the truncated sequence $u_\varepsilon ^{N_\varepsilon }= \sum _{\tau \in \mathcal {T}^{N_\varepsilon }_3} X^\tau _\varepsilon$. This convergence is very delicate, in particular because the number of terms in the sum now grows with $\varepsilon$. The next proposition contains the key estimate that allows us to overcome this issue.

#### Proposition 3.3

Let $T>0$ and $\hat{\lambda }>0$. Then, uniformly over any $\varepsilon \in (0, \tfrac{1}{T} \wedge \tfrac{1}{2})$, $\tau \in \mathcal {T}_{3}^{N_{\varepsilon }}$, with $N_{\varepsilon } = \lfloor \log {\tfrac{1}{\varepsilon }}\rfloor$, and uniformly over all $( t,x) \in [0, T] \times {\textbf {R}}^{2}$, we havewhere  is the trimmed tree $\mathscr {T} ( \tau )$ defined in (2.18) and *C* is the constant defined in (3.4).

The above Proposition both identifies the limit of $\sqrt{\log \tfrac{1}{\varepsilon } } \, X^{\tau }_\varepsilon$ and gives a quantitative estimate of its rate of convergence. The proof of Proposition 3.3 is at the heart of this article and can be found at the end of Sect. 5. It builds on all the results that are derived on the way. We highlight that the bound we obtain is uniform over all trees $\tau \in \mathcal {T}_{3}$ with $| \tau | =O( \log { \tfrac{1}{\varepsilon }} )$. This is rather remarkable: As $| \tau |$ grows, every tree consists of a growing number of components living in distinct homogeneous chaoses and it requires precise estimates to bound all of them at once. It is thus crucial that the right hand-side is summable over $\tau$ and decays for $\varepsilon \rightarrow 0$, in order to justify the asymptoticsas $\varepsilon \rightarrow 0$. The series appearing in the expression above equals3.7where $y( \cdot )$ is the solution of the differential equation $\dot{y}=- y^{3}$ with $y(0)=1$, cf. (2.7), which leads to the expression for the limiting variance in Theorem 1.1. Making the previous argument rigorous is the content of the following proposition, which is the final step towards the proof of Theorem 1.1.

#### Proposition 3.4

Let $\hat{\lambda }>0$ and $T>0$ satisfy

3.8 $$\begin{aligned} \begin{aligned} \hat{\lambda } e^{ \overline{\mathfrak {m}}\, T} < \frac{1}{\sqrt{2C}} \;, \end{aligned} \end{aligned}$$

with $\overline{\mathfrak {m}}= \max \{ \mathfrak {m}\;, 0 \}$ and *C* be the constant defined in (3.4). Then for all $(t, x) \in (0, T] \times {\textbf {R}}^{2}$

$$\begin{aligned} \lim _{\varepsilon \rightarrow 0} \Bigg \Vert \sqrt{\log {\tfrac{1}{\varepsilon }}} \, u_\varepsilon ^{ N_{ \varepsilon }}(t, x) - \hat{\lambda }\, \sigma _{\hat{\lambda }} P_t^{(\mathfrak {m})} \eta (x) \Bigg \Vert _{L^2(\mathbb {P})}= 0\,, \end{aligned}$$

where $\sigma _{{\hat{\lambda }}}$ is as in (3.7).

We remark that condition (3.3) implies (3.8). Neither of them are optimal, however, we distinguish between them to keep track of explicit constants. We will provide the proof of Proposition 3.4 at the end of the next subsection. Before we pass to the proof of Theorem 1.1, let us explain the structure that underlies our main estimate, which is contained in Proposition 3.3.

#### Outline of the structure governing the terms $X^\tau _\varepsilon$

Each Wiener integral $X^\tau _\varepsilon$ is an element of an *inhomogeneous Wiener chaos* (cf. [30]). An element of an inhomogeneous Wiener chaos admits a decomposition into its *homogeneous Wiener chaos* components. These projections are obtained via all possible *pairwise contractions* of noises. In (2.15), this means that $X^\tau _\varepsilon$ can be written as a sum over all possible subsets of pairs of leaves $\kappa \subset \mathcal {L}(\tau )\times \mathcal {L}(\tau )$, where for each $(u,v)\in \kappa$ we replace the product of noise terms $\eta _\varepsilon ( y_u) \eta _\varepsilon ( y_v)$ by $\mathbb {E}[ \eta _{\varepsilon }(y_{u}) \eta _{\varepsilon }(y_{v}) ]= \lambda _{ \varepsilon }^{2} p_{2\varepsilon ^2}(y_u-y_v)$. In the $\varepsilon \rightarrow 0$ limit this corresponds to “contracting” the noises to the “diagonal” $y_v=y_u$, as $p_{2 \varepsilon ^{2}}$ approximates a Dirac $\delta$. A diagrammatic example of a possible contraction (or homogeneous Wiener chaos) configuration is the following:which lies in the first homogeneous Wiener chaos (as only one leaf is uncontracted) and is therefore Gaussian. At the heart of our approach lies the observation that in a contracted tree as depicted above, the eventual contribution to the limit is determined by the total number of so-called 1-*cycles* that one can iteratively extract from the tree. These are cycles that involve two leaves and one inner vertex, such as the one incident on the inner vertex 4 (or equivalently 3) above. The reason for their importance is a time-space decoupling. Indeed the contribution of the cycle based at 4 can be computed explicitly as follows:

3.9 $$\begin{aligned} \lambda _{ \varepsilon }^{2} \int _{({\textbf {R}}^2)^2} p_{s_4}^{(\mathfrak {m})}(x_4-y) p_{2\varepsilon ^2}(y-y') p_{s_4}^{(\mathfrak {m})}(x_4-y') {\,\text {d}}y {\,\text {d}}y' = \lambda _{ \varepsilon }^{2} e^{2\mathfrak {m}\, s_{4}} p_{2(s_4+\varepsilon ^2)}(0),\nonumber \\ \end{aligned}$$

by using the Chapman–Kolmogorov equations, where we denoted the time-space variable associated to the vertex *i* by $(s_{i},x_{i})$. Notably, the result is independent of the space variable $x_{4}$. We can therefore replace the original kernel through a time-dependent kernel based at $x_{4}$ (all the rest unchanged), which graphically we visualize as a red loop around $x_{4}$:Similar approach has been followed for removing the cycle rooted at 3. Now integrating over $x_{4}$, again by the Chapman–Kolmogorov equations as

3.10 $$\begin{aligned}{} & {} \lambda _{ \varepsilon }^{2} \int _{({\textbf {R}}^2)^2} p_{s_2-s_4}^{(\mathfrak {m})}(x_4-x_2) p_{s_4}^{(\mathfrak {m})}(y'-x_4) p_{s_2} ^{(\mathfrak {m})}(y-x_2) p_{2\varepsilon ^2}(y-y') {\,\text {d}}y {\,\text {d}}y' {\,\text {d}}x_4 \nonumber \\{} & {} \quad = \lambda _{ \varepsilon }^{2} e^{2\mathfrak {m}\, s_{2}} p_{2(s_2+\varepsilon ^2)}(0), \end{aligned}$$

we are left with the product between the time-only-dependent kernels in (3.9) and (3.10) and the kernel associated to the tree in which we remove vertex 2 and 4 (and the cycles that are incident to them). This tree now has 1-cycles based at vertices 2 and 4. We can follow the same procedure iteratively for the rest of the cycles as indicated in:removing all 1-cyclcs until there are none left. Hence, in the last diagram we remain with a tree which depicts an iterated (time-only) integral, with (time-only dependent) weights $\lambda _{ \varepsilon }^{ 2}e^{2\mathfrak {m}\, s_{i}} p_{2(s_i+\varepsilon ^2)}(0)$ at every vertex *i*. Note that the ordering of the time variables within this integral is inherited from the tree decorated by loops. A crucial observation will be that only homogeneous chaos configurations which share precisely this property, contribute in the limit $\varepsilon \rightarrow 0$. This will be the content of Sect. 5. We also note that because of the particular structure we have found, determining the eventual limiting contribution is now a simpler task, as we are left with only a time-dependent integral.

How to rigorously determine or estimate the contribution of contracted trees through the removal of certain cycles is the content of the next sections. We will formally introduce contractions (and pairings) and the notion of $\text {v}$-cycles in Sect. 4. In Sect. 5 we will then finally prove Proposition 3.3.

### Proof of Theorem 1.1

We are now ready to prove our main result, given the estimates in Proposition 3.1 and Proposition 3.4, the proofs of which are postponed to further below in the section.

#### Proof of Theorem 1.1

We define $\hat{\lambda }_{\text {fin}}:= \tfrac{1}{10 \sqrt{C}}$, where *C* is the positive constant defined in (3.4). Let $\hat{\lambda } \in (0, \hat{\lambda }_{\text {fin}})$ and $T>0$ such that (1.5) is satisfied, which equivalently reads

3.11 $$\begin{aligned} \begin{aligned} \hat{\lambda } e^{ \overline{\mathfrak {m}} \, T} < \frac{1}{10\sqrt{C}} \;. \end{aligned} \end{aligned}$$

By the triangle inequality, for $\varepsilon \in (0,\tfrac{1}{T} \wedge \tfrac{1}{2})$ and $(t,x) \in (0,T] \times {\textbf {R}}^{2}$

$$\begin{aligned} \begin{aligned} \big \Vert \mathcal {U}_{\varepsilon }(t, x) - \hat{\lambda }\, \sigma _{ \hat{\lambda }} \, P_{t}^{(\mathfrak {m})} \eta (x) \big \Vert _{L^{2}(\mathbb {P})}&\leqslant \sqrt{\log \tfrac{1}{ \varepsilon }} \left\| u_{\varepsilon }(t,x) - u_\varepsilon ^{ N_{ \varepsilon }}(t,x) \right\| _{L^{2}(\mathbb {P})}\\&\qquad + \Big \Vert \sqrt{\log \tfrac{1}{\varepsilon }} \,\, u^{ N_{ \varepsilon }}_{ \varepsilon }(t, x) - \hat{\lambda }\, \sigma _{\hat{\lambda }} \, P_t^{(\mathfrak {m})} \eta (x) \Big \Vert _{L^{2}(\mathbb {P})}\,. \end{aligned} \end{aligned}$$

The second term on the right-hand side vanishes as $\varepsilon \rightarrow 0$ by Proposition 3.4, since (3.11) implies (3.8). On the other hand, by Proposition 3.1, the first term is upper bounded by

$$\begin{aligned} \begin{aligned} \sqrt{\log \tfrac{1}{\varepsilon }} \, \left\| u_{\varepsilon }(t,x) - u_\varepsilon ^{ N_{ \varepsilon }}(t,x) \right\| _{L^{2}(\mathbb {P})} \leqslant \frac{ C_{0}}{\log { \frac{1}{\varepsilon }}} \frac{ \big ( \sqrt{C} \hat{\lambda } e^{ \overline{\mathfrak {m}}\, T}\big )^{N_{ \varepsilon }}}{\varepsilon } \,. \end{aligned} \end{aligned}$$

The blow-up on the right-hand side must be compensated, and here we will crucially use that $N_{ \varepsilon } \sim \log \tfrac{1}{\varepsilon }$ as $\varepsilon \rightarrow 0$, so that we have a compensating effect from the term $\big ( \sqrt{C} \hat{\lambda } e^{ \overline{\mathfrak {m}}\, T}\big )^{N_{ \varepsilon }}$. More precisely, by the choice of *T* in (3.11), we have

$$\begin{aligned} \begin{aligned} - \log {\big ( \sqrt{C} \hat{\lambda } e^{ \overline{\mathfrak {m}}\, T} \big )} \geqslant \log {10} >1\,, \end{aligned} \end{aligned}$$

thus,

$$\begin{aligned} \begin{aligned} \big (\sqrt{C} \hat{\lambda } e^{ \overline{\mathfrak {m}}\, T}\big )^{N_{\varepsilon }} \leqslant \exp \left( \log {\big ( \sqrt{C} \hat{\lambda } e^{ \overline{\mathfrak {m}}\, T}\big )} \left( \log { \tfrac{1}{\varepsilon }} -1 \right) \right) \leqslant 10\, \varepsilon ^{ \log {10}} , \quad \forall \varepsilon \in (0, \tfrac{1}{T} \wedge \tfrac{1}{2}) . \end{aligned} \end{aligned}$$

Hence, we obtain that

$$\begin{aligned} \begin{aligned} \sqrt{\log \tfrac{1}{\varepsilon }} \left\| u_{\varepsilon }(t,x) - u_\varepsilon ^{ N_{ \varepsilon }}(t,x) \right\| _{L^{2}(\mathbb {P})} \leqslant \frac{10\, C_{0}}{\log { \frac{1}{\varepsilon }}} \varepsilon ^{(\log {10}) -1} \,, \end{aligned} \end{aligned}$$

which vanishes in the limit $\varepsilon \rightarrow 0$. This concludes the proof. $\square$

In the remainder of this section we prove that the truncated Wild expansion $u_{ \varepsilon }^{ N_{ \varepsilon }}$ indeed approximates the solution $u_{ \varepsilon }$ (Proposition 3.1) and that $\sqrt{ \log { \tfrac{1}{\varepsilon }}} u_{ \varepsilon }^{N_{ \varepsilon }}(t,x)$ is close to $\hat{\lambda }\, \sigma _{ \hat{\lambda } }P_{t} \eta (x)$ in $L^{2}(\mathbb {P})$ (Proposition 3.4). The proof of Proposition 3.3 will be given at the end of Sect. 5.

#### Proof of Proposition 3.1

Let $T>0$, $(t,x) \in (0 , T] \times {\textbf {R}}^2$ and $\varepsilon \in (0,\tfrac{1}{T} \wedge \tfrac{1}{2})$. From (1.4) and (3.1), we obtain that the difference $w^{N}_{\varepsilon } = u^{N}_{\varepsilon } - u_{\varepsilon }$ solves the equation

3.12 $$\begin{aligned} \begin{aligned} \partial _{t} w^{N}_{\varepsilon } = \frac{1}{2} \Delta w^{N}_{\varepsilon } + \mathfrak {m} w^{N}_{\varepsilon } - (u_\varepsilon ^N)^3 + u_\varepsilon ^{3} + R_\varepsilon ^N\,, \quad w^{N}_{\varepsilon } (0, \cdot ) = 0\,, \end{aligned} \end{aligned}$$

with $R_\varepsilon ^N$ defined in (3.2). Defining $V^{N}_{\varepsilon }(t,x) :=\tfrac{(u_\varepsilon ^N)^3 - u_\varepsilon ^{3}}{u_\varepsilon ^N - u_\varepsilon }$, we can write (3.12) as

$$\begin{aligned} \partial _{t} w^{N}_{\varepsilon } = \frac{1}{2} \Delta w^{N}_{\varepsilon } + \mathfrak {m} w^{N}_{\varepsilon } - V^{N}_{ \varepsilon } \cdot w^{N}_{\varepsilon } + R_\varepsilon ^N\,, \quad w^{N}_{\varepsilon } (0, \cdot ) = 0\,. \end{aligned}$$

The Feynman–Kac formula [29, Theorem 5.7.6] then allows to represent $w^{N}_{\varepsilon }$ as

$$\begin{aligned} w^{N}_{\varepsilon } (t, x)= {\textbf {E}}_x\Bigg [\int _0^t R_\varepsilon ^N(t-s,\beta (s)) \exp \Big ( \mathfrak {m} s -\int _0^s V^{N}_{ \varepsilon }(s-r,\beta (r)) {\,\text {d}}r\Big ) {\,\text {d}}s \Bigg ] \;, \end{aligned}$$

where $\beta (\cdot )$ is a two dimensional Brownian path and ${\textbf {E}}_x$ is the expectation with respect to it when the path starts from $x\in {\textbf {R}}^2$. Using the fact that $V^{N}_{ \varepsilon }\geqslant 0$, which is due to the monotonicity of the mapping $u\mapsto u^3$, we obtain that^3^

$$\begin{aligned}&\big |\, u_\varepsilon ^N (t,x)- u_\varepsilon (t,x) \,\big | \\&\quad \leqslant {\textbf {E}}_x\Bigg [ \int _0^t \big |R_\varepsilon ^N(t-s,\beta (s)) \big | \exp \Big (\mathfrak {m} \, s -\int _0^s V^{N}_{ \varepsilon }(s-r,\beta (r)) {\,\text {d}}r\Big ) {\,\text {d}}s \Bigg ] \\&\quad \leqslant {\textbf {E}}_x\Bigg [ \int _0^t e^{\mathfrak {m}\, s} \big | R_\varepsilon ^N(t-s,\beta (s)) \big | {\,\text {d}}s \Bigg ]\;. \end{aligned}$$

Writing the latter in terms of the heat kernel we conclude that

$$\begin{aligned} \big |\, u_\varepsilon ^N (t,x)- u_\varepsilon (t,x) \,\big |&\leqslant e^{ \overline{\mathfrak {m}}\, t} \int _0^t \int _{{\textbf {R}}^d} p_{t-s}(y-x)\, |R_\varepsilon ^{N}(s,y)| {\,\text {d}}y {\,\text {d}}s \;. \end{aligned}$$

Taking the $L^2(\mathbb {P})$-norm, we arrive at the bound that we will be working with:

3.13 $$\begin{aligned} \Vert u^{N}_{\varepsilon } (t,x)- u_\varepsilon (t,x) \Vert _{L^{2} (\mathbb {P})}&\leqslant e^{\overline{\mathfrak {m}}\, t}\int _0^t \Vert R_\varepsilon ^{N}(s,0) \Vert _{L^{2} (\mathbb {P})} {\,\text {d}}s\,, \end{aligned}$$

where we have used that $R^{N}_{\varepsilon }$ is spatially homogeneous. To continue, we use the definition of $R_\varepsilon ^N$ from (3.2), the triangle inequality and Hölder’s inequality, to obtain3.14At this point we use hypercontractivity, namely estimates of the $L^q(\mathbb {P})$-norm by the $L^p(\mathbb {P})$-norm, for $q>p>1$, for random variables in a fixed inhomogeneous Wiener chaos [27, Theorem 5.10]. In our case it is important to quantify the constant appearing in the hypercontractivity estimates in terms of the chaos level in which the random variable lies. In particular, we will use the following estimate, which is an immediate consequence of [27, Remark 5.11]:

$$\begin{aligned} \Vert X_{\varepsilon }^{\tau _i}(s,y)\Vert _{L^6(\mathbb {P})} \leqslant 5^{\frac{\ell (\tau _i)}{2}}\, \Vert X_\varepsilon ^{\tau _i}(s,y)\Vert _{L^2(\mathbb {P})}\,. \end{aligned}$$

To bound the $L^{2} (\mathbb {P})$-norm, we will make use of Proposition 3.3 to obtain3.15with $\tilde{c} (T,\mathfrak {m}):= e^{ 2|\mathfrak {m}|\, T}+4$. The verification of this bound is deferred to the bottom of this proof. Assuming (3.15) is true, and using the identity  (which holds since $\tau \in \mathcal {T}_{3}$), we obtain3.16Combining (3.13) with (3.14) and (3.16), we therefore conclude that3.17At this point we notice that the time integral appearing in the last estimate blows up polynomially in $\varepsilon$, since

3.18 $$\begin{aligned} \int _0^\infty \frac{1}{(s+\varepsilon ^2)^{\frac{3}{2}} } {\,\text {d}}s = \left[ -2(s+\varepsilon ^2)^{-\frac{1}{2}} \right] _{s=0}^{s= \infty } = \frac{2 }{\varepsilon }. \end{aligned}$$

On the other hand, for any $\{ \tau _{i} \}_{i=1}^{3}$ such that $[\tau _{1} \ \tau _{2} \ \tau _{3}] \not \in \mathcal {T}^{N}_{3}$ we have thatMoreover, by assumption, $\hat{\lambda }$ is sufficiently small to satisfy $5\sqrt{ C}\, \hat{\lambda } e^{ \overline{\mathfrak {m}} \, t} < \frac{1}{2}$. Therefore, we can estimate the sum in the last line of (3.17) as follows. First, observe that3.19Then we complete the remaining sums on the right-hand side to Butcher series:where $\overline{y}$ is the solution to the ODE $\dot{ \overline{y}} =\overline{y}^{3}$ with *positive* initial condition $\overline{y}(0) =1$. The solution of this ODE is $\overline{y}(\zeta )=(1-2\zeta )^{-1/2}$ and so the associated Butcher series converges for $\zeta < 1/2$, i.e. if (3.3) holds in the present case. Thus, with (3.19), we have3.20Hence, combining (3.17), (3.18) and (3.20), we obtain

$$\begin{aligned} \begin{aligned}&\sqrt{\log \tfrac{1}{\varepsilon }} \cdot \Vert u^{N}_{ \varepsilon } (t,\cdot )- u_\varepsilon (t,\cdot ) \Vert _{L^{2} (\mathbb {P})} \leqslant \frac{C_{0}(T, \mathfrak {m}, \hat{\lambda })}{ { \log { \frac{1}{\varepsilon } }}} \frac{\big (\sqrt{C}\hat{\lambda } e^{ \overline{\mathfrak {m}} \, t}\big )^{N} }{\varepsilon } \end{aligned} \end{aligned}$$

with

3.21 $$\begin{aligned} \begin{aligned} C_{0}(T, \mathfrak {m}, \hat{\lambda }) := \frac{ e^{4 \overline{\mathfrak {m}} \,T}}{4} \left( \frac{\sqrt{5} \hat{\lambda }\, \tilde{c} (T,\mathfrak {m}) }{\sqrt{ 1- 10\sqrt{ C}\, \hat{\lambda } e^{ \overline{\mathfrak {m}} \, T} }} \right) ^{3} \,. \end{aligned} \end{aligned}$$

This concludes the proof of the proposition, modulo the proof of (3.15). The latter bound follows simply from the triangle inequality and Proposition 3.3 (which we can apply in view of the constraint $| \tau | \leqslant \lfloor \log {\tfrac{1}{\varepsilon }} \rfloor$):3.22where we made use of the crude estimate

$$\begin{aligned} \begin{aligned} \frac{ e^{ 2|\mathfrak {m}|\, t}+|\log {(t+ \varepsilon ^{2})}| + \sqrt{\log \tfrac{1}{\varepsilon }} }{2 \log { \tfrac{1}{\varepsilon }}} \leqslant \frac{ e^{ 2|\mathfrak {m}|\, t}}{2 \log {2}} +2+ \frac{1}{2 \sqrt{\log {2}} } \leqslant e^{ 2|\mathfrak {m}|\, T}+3, \end{aligned} \end{aligned}$$

which is a consequence of $\varepsilon \in (0, \tfrac{1}{T} \wedge \tfrac{1}{2})$ and the uniform estimate

3.23 $$\begin{aligned} \begin{aligned} \sup _{0 \leqslant t \leqslant T} \frac{|\log (t+\varepsilon ^2)|}{2\,\log \frac{1}{\varepsilon }}&= \sup _{ 0 \leqslant t \leqslant 1- \varepsilon ^{2}} \frac{|\log (t+\varepsilon ^2)|}{2\,\log \frac{1}{\varepsilon }} \vee \sup _{ 1- \varepsilon ^{2}<t< T} \frac{\log (t+\varepsilon ^2)}{2\,\log \frac{1}{\varepsilon }}\\&\leqslant 1+ \frac{ \log {( \tfrac{1}{\varepsilon }+1)}}{2\,\log \frac{1}{\varepsilon } } \leqslant 2 \,. \end{aligned} \end{aligned}$$

Now the statement follows, sincewhere *C* is the constant from (3.4). Thus together with (3.22), we obtainwith $\tilde{c} (T,\mathfrak {m})=e^{ 2|\mathfrak {m}|\, T}+4$. This completes the proof. $\square$

#### Proof of Proposition 3.4

For $h(y)=-y^3$, we introduce the truncated Butcher series3.24where the second equality is a consequence of Lemma 2.3. Therefore, Proposition 3.4 will follow if we can show that the following two limits hold true with $\zeta _{ \hat{\lambda }} = \tfrac{3 \hat{\lambda }^{2}}{2 \pi }$:

3.25 $$\begin{aligned}&\lim _{ \varepsilon \rightarrow 0} \,\, \Big \Vert \sqrt{\log {\tfrac{1}{\varepsilon }}} \, u_\varepsilon ^{N_{ \varepsilon }} (t, x) - \hat{\lambda } B^{\varepsilon }_{h} (\zeta _{ \hat{\lambda }}, 1) \, e^{\mathfrak {m}\, t} P_{t+ \varepsilon ^{2}} \eta (x) \,\Big \Vert _{L^{2} (\mathbb {P})} = 0, \end{aligned}$$

3.26 $$\begin{aligned}&\lim _{\varepsilon \rightarrow 0} \,\, \Big \Vert \hat{\lambda } B^{\varepsilon }_{h} (\zeta _{ \hat{\lambda }}, 1)\, e^{\mathfrak {m}\, t} P_{t+ \varepsilon ^{2}} \eta (x) - \hat{\lambda }\, \sigma _{ \hat{\lambda }} P^{(\mathfrak {m})}_{t} \eta (x) \,\Big \Vert _{L^{2} (\mathbb {P})} = 0 . \end{aligned}$$

The limit (3.25) follows from Proposition 3.3, provided (3.8) holds. Indeed, via the named proposition, we can bound for $\varepsilon \in (0,\tfrac{1}{T} \wedge \tfrac{1}{2})$3.27where we used (3.24). Now, (3.25) will follow, if we can show that the series on the right-hand side is summable, that is if

3.28 $$\begin{aligned} \begin{aligned} \sum _{ \tau \in \mathcal {T}_{\leqslant 3} }\frac{|h^{( \tau )} (1)|}{ s(\tau )\ \tau !} \left( C \hat{\lambda }^2 e^{2 \overline{\mathfrak {m}}\, t}\right) ^{|\tau |} < \infty . \end{aligned} \end{aligned}$$

This is the Butcher series associated to the ODE $\dot{ \overline{y}} =\overline{y}^{3}$ with initial condition $\overline{y}(0) =1$, which converges as long as (3.8) holds. See also the discussion in the proof of Proposition 3.1. Hence, (3.27), and thus (3.25), vanish for arbitrary fixed $t \in (0,T]$, because the second-to-last ratio on the right-hand side in (3.27) vanishes in the limit $\varepsilon \rightarrow 0$.

To complete the proof of the proposition we must now check (3.26). Here we observe that

$$\begin{aligned} \begin{aligned}&\Big \Vert \, \hat{\lambda } B^{\varepsilon }_{h} (\zeta _{ \hat{\lambda }}, 1) \, e^{ \mathfrak {m} \, t} P_{t+ \varepsilon ^{2}} \eta (x) - \hat{\lambda }\, \sigma _{\hat{\lambda }}\, P_{t}^{( \mathfrak {m})}\eta (x) \, \Big \Vert _{L^{2} (\mathbb {P})}\\&\quad \leqslant \hat{\lambda }\, | B^{\varepsilon }_{h} (\zeta _{ \hat{\lambda }}, 1) - \sigma _{ \hat{\lambda }}|\cdot \big \Vert e^{ \mathfrak {m} \, t} P_{t+ \varepsilon ^{2}} \eta (x) \,\big \Vert _{L^{2} (\mathbb {P})}\\&\qquad + \hat{\lambda }\, \sigma _{ \hat{\lambda }} \, \big \Vert e^{ \mathfrak {m} \, t} P_{t+ \varepsilon ^{2}} \eta (x) - P_{t}^{(\mathfrak {m})} \eta (x) \big \Vert _{L^{2} (\mathbb {P})}. \end{aligned} \end{aligned}$$

The second term is converging to 0 by the continuity properties of the heat semigroup. Instead, for the first term we observe that $B^{\varepsilon }_{h} (\zeta , 1)$ is an approximation to the Butcher series associated to the solution $y(\zeta )$ of the ODE $\dot{ y} = - y^{3}, y(0)=1$, which is given by $y(\zeta ) = (1 + 2 \zeta )^{-1/2}$. This solution is analytic for $| \zeta | < 1/2$ and the associated Butcher series converges (see the discussion in Sect. 2.2). Thus, recalling the form of $\sigma _{{\hat{\lambda }}}$ (3.7), we have that $\lim _{ \varepsilon \rightarrow 0}| B^{\varepsilon }_{h} (\zeta _{ \hat{\lambda }}, 1) - \sigma _{ \hat{\lambda }}| =0$, as long as $\frac{3 \hat{\lambda }_{\text {fin}}^{2}}{2 \pi } < \frac{1}{2} \;,$ which is implied by (3.8). This concludes the proof. $\square$

## Contracted trees, Wiener chaoses and their structure

In Sect. 3.1, and in particular in Sect. 3.1.1, we outlined the structure underlying the main estimate contained in Proposition 3.3. In this section we will introduce the notion of contraction and present estimates on the integration kernels associated to the Wild expansion terms $X_{ \varepsilon }^{ \tau }$, which will allow us to analyze them rigorously in Sect. 5.

### Wiener chaos decomposition, contractions and cycles

The multiple stochastic integrals $X^{\tau }$ appearing in the Wild expansion (1.4) lie in the $\ell (\tau )$-th *inhomogeneous* Wiener chaos. Elements in a finite inhomogeneous Wiener chaos can be decomposed into terms belonging to distinct *homogeneous* Wiener chaoses. We refer to [27, Chapter 2] and [30, Chapter 1] for a more detailed discussion about Wiener spaces and their decomposition to homogeneous components.

Our asymptotic analysis builds on a precise understanding of the decomposition of the components of the Wild expansion into its homogeneous chaos terms. The goal of this detailed study will be to show that only terms in the first chaos (and not all of them) contribute to the Gaussian limit in Theorem 1.1.

In our setting, homogeneous components of the Wild expansion will be represented by stochastic integrals indexed by trees with additional *contraction in pairs* between elements of a subset of their leaves. A *contraction* of a given tree is a pairing among the elements of an arbitrary subset of the leaves of the tree. Unlike the stochastic integrals indexed by the initial tree, the stochastic integral indexed by a contracted tree lies in a homogeneous chaos, whose order is given by the number of uncontracted leaves. One can then recover the integral associated to the original tree by summing over integrals indexed by the same tree with all possible contractions. Let us now be more precise and start with the definition of a contraction.

#### Definition 4.1

For any $\tau \in \mathcal {T}$ we define a **contraction** to be a subset $$\kappa \subset \left( {\begin{array}{c}\mathcal {L}(\tau )\\ 2\end{array}}\right) $$, where $$\left( {\begin{array}{c}\mathcal {L}(\tau )\\ 2\end{array}}\right) $$ denotes the set of all unordered pairs of leaves of the tree $\tau$, such that every $v\in \mathcal {L}(\tau )$ lies in at most one element of $\kappa$. We define the corresponding *set of contractions* by

4.1 $$\begin{aligned} \mathcal {K}(\tau ):=\left\{ \kappa \subset \left( {\begin{array}{c}\mathcal {L}(\tau )\\ 2\end{array}}\right) \,:\, \kappa \text { is a contraction of }\tau \right\} \,. \end{aligned}$$

Furthermore, we denote a tree $\tau = (\mathcal {V}, \mathcal {E})$ that is being contracted according to a contraction $\kappa$ by $\tau _\kappa$:

$$\begin{aligned} \begin{aligned} \tau _{\kappa } := (\mathcal {V}, \mathcal {E}\cup \kappa ) \;, \end{aligned} \end{aligned}$$

and call this a $\kappa$-*contracted tree* or simply a *contracted tree*. We will also denote by $\mathcal {L}(\tau _\kappa )$ the set of leaves of $\tau _\kappa$, namely, the set of leaves of $\tau$ which are not included in the contraction $\kappa$.

If all leaves of $\tau$ are contracted via $\kappa$ we will call $\kappa$ a *complete contraction* and $\tau _\kappa$ a *completely contracted* tree. If this is not the case, we will often talk of *partial contraction* and a *partially contracted* tree.

Graphically, a contracted tree $\tau _\kappa$ is represented by the original graph of $\tau$ augmented with edges connecting the pairs of vertices in $\kappa$. We will colour the additional edges arising from $\kappa$ in red. For example, the possible contractions of the tree4.2are (up to symmetries)4.3where we denoted the tree without any contraction with a subscript $\emptyset$ to emphasize the empty contraction.^4^ Intuitively, when seen as a stochastic integral, the uncontracted vertices in $\tau _\kappa$ will have all assigned space variables being distinct, while the edge with space variables $(y_{u_1},y_{u_2})$ connecting a pair of $(u_1,u_2) \in \kappa$ will be assigned a weight $p_{2\varepsilon ^2}(y_{u_2}-y_{u_1})$. More precisely, to any contracted tree $\tau _\kappa$ we associate a (homogeneous) Wiener integral lying in a *homogeneous* Wiener chaos through the following definition:

4.4 $$\begin{aligned}&{ \tau }_{\kappa , \varepsilon } (t, x) : = \int _{ D_{t}^{\mathcal {V}(\tau )\setminus \mathfrak {o}} } K_{\tau _{\kappa }, \varepsilon }^{t, x} (s_{\mathcal {V}(\tau )}, y_{\mathcal {V}(\tau )} ) \, {\,\text {d}}y_{\mathcal {V}(\tau )\setminus (\mathfrak {o} \cup \mathcal {L}(\tau _{\kappa })) }{\,\text {d}}s_{\mathcal {V}( \tau ) \setminus \mathfrak {o}} \ \eta _\varepsilon ( {\,\text {d}}y_{\mathcal {L}(\tau _\kappa )}) \end{aligned}$$

with $$D_{t}= [0,t]\times {\textbf {R}}^{2}$$ and

4.5 $$\begin{aligned} K_{\tau _{\kappa }, \varepsilon }^{t, x} (s_{\mathcal {V}(\tau )}, y_{\mathcal {V}(\tau )})&:= \prod _{u \in \mathcal {V}( \tau )\setminus \mathfrak {o}} p_{s_{\mathfrak {p}(u)} - s_{u}}^{( \mathfrak {m })}(y_{\mathfrak {p}(u)} - y_{u}) \Bigg \{ \prod _{v \in \mathcal {L}(\tau _\kappa )} \delta _{0}(s_v)\Bigg \} \nonumber \\&\quad \times \prod _{(u_1,u_2) \in \kappa } \lambda _{\varepsilon }^{2} \, \delta _{0}(s_{u_1}) \, \delta _{0}(s_{u_2}) \, p_{2\varepsilon ^2}(y_{u_2} - y_{u_1}) \, , \end{aligned}$$

with $\mathfrak {p}(u)$ denoting the parent of *u* and $( s_{\mathfrak {o}}, y_{\mathfrak {o}})= (t,x)$. To lighten notation, we will often drop the index *t*, *x* that indicates the time-space coordinates of the root, if the explicit indication is not necessary. We stress the difference in notation of the two types of stochastic integrals once and for all here: For the homomgeneous Wiener integral (4.4) we write $\eta _\varepsilon ( {\,\text {d}}y_{\mathcal {L}(\tau )})$, whereas for the inhomogeneous Wiener integral (2.15) we use $\prod _{v \in \mathcal {L}( \tau )} \eta ({\,\text {d}}y_{v})$.

With this definition, $\tau _{\kappa ,\varepsilon }$ lies in the homogeneous Wiener chaos of order $\ell (\tau _k)=|\mathcal {L}(\tau _\kappa )|$. Given the decomposition of an element in a Wiener chaos into its homogeneous components, see [27, Remark 7.38], we have that for any $\tau = [ \tau _{1} \ \ldots \ \tau _{n}]$, the associated stochastic integral $\tau _\varepsilon$ can be decomposed as follows

4.6 $$\begin{aligned} \tau _\varepsilon \, := [\tau _{1}]_{\varepsilon }\cdots [ \tau _{n}]_{ \varepsilon } = \sum _{\kappa \in \mathcal {K}(\tau )} \tau _{\kappa , \varepsilon }\,, \end{aligned}$$

with $[ \tau _{i}]_{ \varepsilon }$ defined in (2.15). In the example of the trees in (4.2) and (4.3), we have that the decomposition of the inhomogeneous element represented by the tree (4.2) to its homogeneous components is given bywhere the right-hand side corresponds to the homogeneous stochastic integrals indexed by the contracted trees in (4.3). Here we have taken into account multiplicities of homogeneous components due to equivalent contractions. For example, the contractionsare all different. However, they correspond to the same stochastic integrals. Lastly, let us mention that for a planted tree $[ \tau ]$ we use both notations $[ \tau ]_{\kappa }$ and $[ \tau _{ \kappa }]$ for a contracted version of that tree.

**Contractions between trees and** $L^{2}(\mathbb {P})$ **estimates.** We now want to extend the notion of contraction from within a single tree to a pair of trees. This will be necessary in order to encode second moments of stochastic integrals of the form (4.4). The Gaussianity and correlation structure of the white noise, imply via Wick’s theorem, that the second moment can be expressed as the sum over all possible *pairwise contractions* over the (uncontracted) leaves (or precisely over the noise variables that lie on the leaves) of two copies of the tree, connected to the same root with time-space variables (*t*, *x*). In other words, we look at the stochastic integral $[\tau ,\tau ]_\varepsilon (t,x)$ corresponding to the tree $[\tau ,\tau ]$ with root variable (*t*, *x*). Let us look at the example of computing the second moment of the stochastic integral , evaluated at a time-space point (*t*, *x*). Its second moment will be represented by4.7In other words, the computation of the second moment of a stochastic integral $[\tau ]_\varepsilon$ gives rise to a *completely* contracted tree $[\tau ,\tau ]_\kappa$ in accordance with the definition of (4.1). We note that, if we first decompose $[\tau ]_\varepsilon (t,x)$ into its homogeneous Wiener chaos components, then an alternative computation would yieldwhere we used the orthogonality between different homogeneous chaos components.

Let us introduce a notation that will allow us to encode contractions between trees that are glued together, in a way that distinguishes them from the contractions of Definition 4.1. This will be useful to encode covariances between $[\tau ]_\varepsilon {(t, x)}$ and $[\tau ']_\varepsilon {(t, x)}$.

#### Definition 4.2

For two rooted trees $\tau , \tau ' \in \mathcal {T}$ define the set of **pairings** among the union of leaves as

$$\begin{aligned} \mathcal {Y}(\tau , \tau ') := \big \{\gamma \in \mathcal {K}([\tau ,\tau ']): \gamma \text { is a complete contraction }\big \}. \end{aligned}$$

We also define the subsets of pairings which complete a given pair of contractions $(\kappa , \kappa ^{\prime } ) \in \mathcal {K}(\tau ) \times \mathcal {K}(\tau ^{\prime })$ by

$$\begin{aligned} \mathcal {Y}(\tau _\kappa , \tau '_{\kappa '}) := \big \{\gamma \in \mathcal {Y}(\tau ,\tau ')\,:\, \kappa \cup \kappa ' \subset \gamma \text { and all pairs in }\gamma \setminus ( \kappa \cup \kappa ')\text { connect } \tau \text { to }\tau ' \big \}. \end{aligned}$$

We will write $[\tau ,\tau ']_\gamma$ to denote the tree $[\tau ,\tau ']$ where all leaves are contracted according to $\gamma \in \mathcal {Y}(\tau ,\tau ')$.

The pictorial representations in (4.7) show all possible elements (up to symmetries) of $\mathcal {Y}(\tau ,\tau )$ for that example. Furthermore, note that for any two contracted trees $\tau _{\kappa } , \tau _{\kappa ^{\prime }}^{\prime }$, and $\gamma \in \mathcal {Y}(\tau _\kappa , \tau '_{\kappa '})$, the pairing $[\tau _{\kappa } , \tau ^{\prime }_{\kappa ^{\prime }}]_{\gamma }$ gives rise to a *completely contracted* tree and, therefore, $\mathcal {Y}(\tau _\kappa , \tau '_{\kappa '})=\emptyset$, if the number of uncontracted leaves in $\tau _\kappa$ and $\tau '_{\kappa '}$ differ. This agrees with the fact that homogeneous chaoses are orthogonal with respect to one another. We can now express covariances between contracted trees as follows:

4.8 $$\begin{aligned} \begin{aligned} \mathbb {E}\Big [\,[\tau ]_{\kappa , \varepsilon } \, [\tau ']_{\kappa ',\varepsilon }\,\Big ] = \sum _{\gamma \in \mathcal {Y}(\tau _{\kappa },\tau '_{\kappa '})} [\, \tau ,\tau ']_{\gamma , \varepsilon }\,. \end{aligned} \end{aligned}$$

Moreover, it is clear that $\mathcal {Y}(\tau _{\kappa }, \tau '_{ \kappa '} )$ allows to partition $\mathcal {Y}( \tau , \tau ')$ as

4.9 $$\begin{aligned} \begin{aligned} \mathcal {Y}( \tau , \tau ') = \bigsqcup _{ (\kappa , \kappa ') \in \mathcal {K}( \tau ) \times \mathcal {K}( \tau ')} \mathcal {Y}(\tau _{\kappa }, \tau '_{ \kappa '} )\,, \end{aligned} \end{aligned}$$

where $\bigsqcup$ denotes a disjoint union. This is clear since, if we want to find all pairwise contractions of $[\tau ,\tau ']$, we can first identify the contractions that are internal to each $\tau ,\tau '$ and then identify the contractions that connect the leaves of one tree to those of the other. This partitioning then allows us to express covariances in terms of

$$\begin{aligned} \begin{aligned} \mathbb {E}\Big [\,[\tau ]_\varepsilon \, [\tau ']_\varepsilon \,\Big ]&= \sum _{\kappa \in \mathcal {K}(\tau )} \sum _{\kappa ' \in \mathcal {K}(\tau ')} \mathbb {E}\Big [\,[\tau ]_{\kappa , \varepsilon } \, [\tau ']_{\kappa ',\varepsilon }\,\Big ] \\&= \sum _{\kappa \in \mathcal {K}(\tau )} \, \sum _{\kappa ' \in \mathcal {K}(\tau ')} \, \sum _{\gamma \in \mathcal {Y}(\tau _{\kappa },\tau '_{\kappa '})} [\, \tau ,\tau ']_{\gamma , \varepsilon }\\&= \sum _{\gamma \in \mathcal {Y}(\tau ,\tau ')} [ \,\tau ,\tau ']_{\gamma , \varepsilon }\,, \end{aligned} \end{aligned}$$

where we used (4.8) in the second step, and (4.9) in the last. Finally, the partitioning (4.9) allows us to recover the internal contractions associated to a given pairing. This motivates the following definition.

#### Definition 4.3

For any $\tau , \tau ' \in \mathcal {T}_{3}$, let $\mathfrak {s}_{ [ \tau , \tau ']} : \mathcal {Y}( \tau , \tau ') \rightarrow \mathcal {K}( \tau ) \times \mathcal {K}( \tau ')$ be the map that for any $\gamma \in \mathcal {Y}( \tau , \tau ')$ identifies the unique pair $\mathfrak {s}_{ [\tau , \tau ']}(\gamma ) := (\kappa _1(\gamma ), \kappa _{2}( \gamma ))$ such that

$$\begin{aligned} \begin{aligned} \gamma \in \mathcal {Y}( \tau _{ \kappa _{1}( \gamma )}, \tau '_{ \kappa _{2} (\gamma )})\,. \end{aligned} \end{aligned}$$

In other words, the map $\mathfrak {s}$ identifies the subset of edges in $\gamma$ that only connect within $\tau$ and $\tau '$, respectively.

### 1-cycles and their removal

Let us now introduce the notion of a *1-cycle*. Suppose that a contracted tree $\tau _\kappa$ contains a component of the form4.10namely where we observe a cycle consisting of an inner vertex $(s_1,y_1)$ connected to two leaves that are themselves connected to one another by a red edge (part of $\kappa$). We call such a cycle a 1-*cycle*. Let us remark that in the above picture, the point $(s_2,y_2)$ denotes the coordinates of the basis of the sub-tree $\tau _1$ and $(s_0,y_0)$ denotes the coordinates of the parent of the inner vertex with coordinates $(s_1,y_1)$. A more formal definition is the following.

#### Definition 4.4

Given a tree $\tau$ and a contraction $\kappa \in \mathcal {K}(\tau )$, we call a 1-*cycle* a connected component of $\tau _\kappa$ which consists of two leaves, which are connected by an element of $\kappa$, and the inner vertex, which is the parent of these leaves, as well as the three edges that connect these three vertices. We call the inner vertex of the cycle the basis of the 1-cycle.

Given a contracted tree $\tau _\kappa$ with a 1-cycle $\mathcal {C}$, we write $(\tau \setminus \mathcal {C})_{ \widetilde{\kappa }}$ for the contracted tree that is obtained by “removing” the cycle $\mathcal {C}$ from $\tau _\kappa$. That is, the graph that remains after removing all edges and vertices that belong to $\mathcal {C}$ and replacing the remaining two edges which used to connect to the basis of the 1-cycle by a new single edge, which connects the only remaining descendant of the basis we removed to the parent of this basis. The contraction $\widetilde{\kappa }$ is the one induced naturally on $\tau \setminus \mathcal {C}$ by $\kappa$ after the removal of the element that connects the leaves of $\mathcal {C}$. The removal is simply described by the following picture:4.11Observe that if $\tau \in \mathcal {T}_{3}$, then also $\tau \setminus \mathcal {C}\in \mathcal {T}_{3}$. An important lemma is the following, which records the effect of the 1-cycle on the associated stochastic integrals.

#### Lemma 4.5

Let $$ \tau \in \mathcal {T}$$ be of the form $$ \tau = [ \tau _{1} \cdots \tau _{n}]$$, $$ \tau _{i} \in \mathcal {T}_{3}$$, and $$ \kappa \in \mathcal {K}( \tau ) $$. Further, let $$ \mathcal {C}$$ be a 1-cycle in the contracted tree $$ \tau _{\kappa } $$ with the coordinates of its root being $$(t,x) \in (0, \infty ) \times {\textbf {R}}^{2}$$. Denote by $$(s_{1}, y_{1}) $$ the coordinates of the basis of $$\mathcal {C}$$, by $$(s_{0}, y_{0}) $$ the coordinates of the parent of $$(s_{1}, y_{1}) $$ and by $$(s_{2}, y_{2}) $$ the coordinates of the only descendant of $$(s_{1}, y_{1})$$ that does not belong to $$\mathcal {C}$$. Denote also by $$ z_{3} = (s_{3}, y_{3}) $$ and $$ z_{4} = (s_{4} , y_{4}) $$ the coordinates of the leaves of the 1-cycle $$ \mathcal {C}$$ (where we recall that the time coordinates $$s_3$$ and $$s_4$$ of the leaves will coincide with 0). Then4.12where the kernel *K* is defined in (4.5), $$ D_{t} $$ is defined in (2.16) and4.13$$ \widetilde{\tau } := \tau \setminus \mathcal {C}$$ and $$ \widetilde{\kappa } $$ the contraction induced on $${\tilde{\tau }}$$ by the removal of $$\mathcal {C}$$ from $$\tau _\kappa $$.

#### Proof

We start by performing the integration over the spatial coordinates $y_{3}, y_{4}$ of the part of the kernel $K_{\tau _{\kappa , \varepsilon }}^{t,x} (s_{\mathcal {V}}, y_{\mathcal {V}})$ that depends on the variables $z_3$ and $z_4$. This corresponds to the following integral (recall form (4.5) that the kernel $K_{\tau _{\kappa , \varepsilon }}^{t,x} (s_{\mathcal {V}}, y_{\mathcal {V}} )$ contains factors $\delta _0(s_3) \,\delta _0(s_4)$):4.14Next, we integrate the remaining part of the kernel over $y_1$. This reduces to the Chapman–Kolmogorov identity (refer also to the pictures in (4.10) and (4.11) for guidance):

$$\begin{aligned} \int _{{\textbf {R}}^2} p_{s_1-s_2}^{( \mathfrak {m })}(y_2 - y_1) p_{s_0-s_1}^{( \mathfrak {m })} (y_1 - y_0) \,{\,\text {d}}y_1 = p_{s_0-s_2}^{( \mathfrak {m })}(y_2 - y_0). \end{aligned}$$

Combining the results of the two integrations above with the remaining components of the kernel $K_{\tau _{\kappa , \varepsilon }}^{t,x} (s_{\mathcal {V}}, y_{\mathcal {V}})$, yields the expression on the right-hand side of (4.12). $\square$

As it turns out, 1-cycles play an important role in our analysis. We will see that the contracted trees in the Wild expansion that contribute to the limiting fluctuations, are exactly those whose contraction consists of only 1-cycles (which may also emerge in an iterative way, see the second example below). To get an idea of this phenomenon, let us look at the following examples.

#### Example 1

Consider the contracted tree . Using Lemma 4.5 (or in this case even a by-hand computation) we find thatwhere inside the integral (*s*, *y*) are the time-space coordinates associated to the basis of the trident. We can next compute the spatial integral via Chapman–Kolmogorov asTherefore, we obtainand hence, by Lemma A.1 and definition of $\lambda _{ \varepsilon }$, for $t \in (0, \infty )$ we find thatwhere the *o*(1) is with respect to $\varepsilon \rightarrow 0$.

#### Example 2

This example demonstrates the iterative appearance of 1-cycles, after successive extractions, and their overall contribution. Consider the contracted treewhere we have tagged some vertices for reference in the following integrals. In particular, the coordinates of vertex *i* will be $(s_i,y_i)$. First, extracting the 1-cycle with basis 2, we have that, using Lemma 4.5,Now, applying once more Lemma 4.5 on the kernel, or just the previous example, the above integral equalsThus, the contribution of this diagram is of the same order as in the previous example (albeit with a different constant) and will also contribute to the limiting Gaussian fluctuations.

Following the same steps as above, we can determine similarly the contribution of the contracted tree

### $\text {v}$-cycles and their removal

Contrary to the above two examples, where only 1-cycles appeared, the next example will demonstrate a different cycle structure, which will lead to lower order contributions. This will motivate the study of $\text {v}$-cycles of arbitrary length, which will play an important role in our analysis.

#### Example 3

Let us look at the order of magnitude of . Its second moment has the diagrammatic representation in terms of the completely contracted treewhere the factor 6 counts the number of symmetries of the pairing at hand. Denoting by $(s_1,y_1)$ and $(s_2,y_2)$ the time-space coordinates of the bases of the left and right tridents, respectively, we can explicitly write the integral corresponding to the above diagram as

$$\begin{aligned} 6 \lambda _\varepsilon ^6 \int _{D_{t}^2} p_{t-s_1}^{(\mathfrak {m})}(y_1-x) \, \big ( e^{\mathfrak {m}\, ( s_{1}+ s_{2})} p_{s_1+s_2+2\varepsilon ^2}(y_1-y_2) \big )^3 \, p_{t-s_2}^{( \mathfrak {m})}(y_2-x) {\,\text {d}}y_{1} {\,\text {d}}y_2 {\,\text {d}}s_1 {\,\text {d}}s_{2}. \end{aligned}$$

Then, using the estimate $( e^{\mathfrak {m}\, ( s_1 + s_2)} p_{s_1+s_2+2\varepsilon ^2}(y_1-y_2))^2\leqslant e^{4 \overline{\mathfrak {m}}\, t}(2\pi (s_1+s_2+2\varepsilon ^2))^{-2}$ together with Chapman–Kolmogorov, we can bound this by

$$\begin{aligned} 6 \lambda _\varepsilon ^6 e^{4 \overline{\mathfrak {m}}\, t} \int _{D_{t}^2}&\frac{ p_{t-s_1}^{(\mathfrak {m})}(y_1-x) \, e^{ \mathfrak {m}\, ( s_{1}+ s_{2})} p_{s_1+s_2+2\varepsilon ^2}(y_1-y_2) \, p_{t-s_2}^{(\mathfrak {m})}(y_2-x)}{\big ( 2\pi (s_1+s_2+2\varepsilon ^2)\big )^2} {\,\text {d}}y_{1} {\,\text {d}}y_2 {\,\text {d}}s_1 {\,\text {d}}s_2 \\&\leqslant \frac{6 \lambda _\varepsilon ^6}{(2\pi )^2} e^{6 \overline{\mathfrak {m}} \, t} p_{2(t+\varepsilon ^2)}(0) \int _{[0,t]^2} \frac{1}{(s_1+s_2+2 \varepsilon ^2)^2} {\,\text {d}}s_1 {\,\text {d}}s_2 \\&\leqslant \frac{6 \lambda _\varepsilon ^6}{(2\pi )^2} \log (1+\tfrac{1}{2}t\varepsilon ^{-2})\, e^{6 \overline{\mathfrak {m}}\, t} p_{2(t+\varepsilon ^2)}(0) \,. \end{aligned}$$

Since $\lambda _\varepsilon ^6 = O((\log \tfrac{1}{\varepsilon })^{-3})$, we can conclude that  for some constant *C*(*t*) only depending on $t>0$.

In the last example there was no 1-cycle appearing. Instead, the contracted tree that emerged from the diagrammatic representation of the second moment, presented cycles containing *more than one* inner vertex, with every edge of the cycle incident to at least one leaf. We will call such cycles $\text {v}$-*cycles*. The emergence of $\text {v}$-cycles and the quantitative estimate of their contribution will play a crucial role. The key observation is that contracted trees which do not consist of 1-cycles *only*, will have their second moment represented by a paired tree which necessarily contains a $\text {v}$-cycle of length strictly greater than one. Such trees will turn out to have a lower order contribution. The main estimates in this section, which provide a quantitative control on $\text {v}$-cycles, are given in Lemmas 4.9 and 4.10 below. Let us start with the rigorous definition of a $\text {v}$-cycle.

#### Definition 4.6

For a given contracted tree $\tau _{\kappa },$ a subgraph $\mathcal {C}= (\mathcal {V}_{\mathcal {C}}, \mathcal {E}_{\mathcal {C}}) \subseteq \tau _{\kappa }$ is a $\text {v}$-**cycle** if it is a cycle in $\tau _{\kappa }$ (viewed as a graph) in which every edge is incident to at least one leaf of the tree $\tau$. We define the *length* of a $\text {v}$-cycle to be the number of inner vertices of $\tau$ contained in $\mathcal {C}$ and we also denote by $\mathcal {I}_{\mathcal {C}}$ and $\mathcal {L}_{\mathcal {C}}$ the collection of the inner vertices and leaves of $\tau$ that belong to $\mathcal {C}$, respectively. We will call a $\text {v}$-cycle of length $m \in {\textbf {N}}$ a *m*-cycle for short.

A pictorial example of a $\text {v}$-cycle is the one that appears in the following component of a contracted tree:Note that a 1-cycle (Definition 4.4) is simply a $\text {v}$-cycle of length 1. Consider a $\text {v}$-cycle of length *m* and denote its inner vertices by $v_1,...,v_m$, where we will always keep the convention that in such an encoding we start from the left-most inner vertex of the cycle, in the graph picture of the tree, and register the following inner vertices as we trace the cycle clock-wise. Let us also denote the time coordinates of $v_1,...,v_m$ by $s_1,...,s_m$, respectively. We introduce the following kernel, which will play an important role in our estimates:4.15with the convention that $s_{m+1}=s_1$. We note that the above kernel is invariant under cyclic permutation of $s_1, s_2,...,s_m$.

The following lemma, which will be proved in Appendix A.3, establishes the existence of a $\text {v}$-cycle in a completely contracted tree of the form $[\tau _1,\tau _2]$.

#### Lemma 4.7

Let $\tau _1,\tau _2 \in \mathcal {T}_3$ . Then, for every pairing $\gamma \in \mathcal {Y}(\tau _1,\tau _2)$, the paired tree $[\tau _1,\tau _2]_\gamma$ contains a $\text {v}$-cycle.

An important procedure that we will follow in the remainder of the section, is to spatially decouple $\text {v}$-cycles from the rest of the integration kernel encoded by the tree. We will be performing such decoupling estimates sequentially until we exhaust all $\text {v}$-cycles, including the $\text {v}$-cycles that will emerge through this process. We will call this process *cycle removal*. Below we formally define the cycle removal of a single cycle.

#### Definition 4.8

*(Cycle removal)* For any contracted tree $\tau _{\kappa }$, with $\tau \in \mathcal {T}$ of the form $\tau = [ \tau _{1} \cdots \tau _{n}]$, $\tau _{i} \in \mathcal {T}_{3}$, and any $\text {v}$-cycle $\mathcal {C}\subseteq \tau _{\kappa }$ (not passing through the root), we define the contracted tree $( \tau \setminus \mathcal {C})_{ \widetilde{ \kappa }}$ to be the contracted tree obtained from $\tau _{\kappa }$ through the following procedure:

1. Remove from $\tau _\kappa$ all the edges and vertices that belong to $\mathcal {C}$.
2. We note that an inner vertex that belongs to $$\mathcal {C}$$ will always have four neighbours due to the tree being built from ternary trees. Let *v* be an inner vertex that belongs to $$\mathcal {C}$$. We then write $$ v_{0}= \mathfrak {p}(v)$$ for its parent and $$ v_{2}$$ for the unique descendant which is not connected to *v* by an edge in $$\mathcal {C}$$ (recall the representation (4.11)). We then distinguish between the following two cases:
  - Both $$ v_{0}$$ and $$ v_{2}$$ are not part of $$\mathcal {C}$$, then we replace the two edges $$ \{ v_{0}, v \} $$ and $$\{ v_{2}, v \} $$ by a single edge $$ \{ v_{0}, v_{2} \} $$.
  - If $$ v_{0}$$ (or $$ v_{2}$$) are part of $$\mathcal {C}$$, we proceed going down (or up) in the tree until we find a vertex $$ w_{0}$$ (or $$ w_{2}$$) not being part of $$\mathcal {C}$$. Once, we found such vertices we remove all edges crossed in this exploration (which don’t belong to $$\mathcal {C}$$ since they connect inner nodes) and replace them by the edge $$ \{w_{0}, w_{2}\}$$.^5^

The result of this procedure is a tree $\tau \setminus \mathcal {C}$ with the contraction $\widetilde{\kappa }$ consisting of the remaining edges in $\kappa$ after the above two steps.

For example, consider the following component of a treeIn this example there are three $\text {v}$-cycles: one whose contraction-edges consists of only the blue edges, one whose $\kappa$ edges consist of the top blue edge and the red one and, finally, one whose $\kappa$ edges consists of the red one and the two blue ones below the red. The process of removing the blue $\text {v}$-cycle is depicted below. The middle step shows the component after removing the leaves and edges that are part of the $\text {v}$-cycle. The rightmost tree is the final outcome after also replacing the edges incident to the inner vertices of the $\text {v}$-cycle by a single edge.The following lemma provides the central estimate that quantifies the contributions coming from $\text {v}$-cycles.

#### Lemma 4.9

Let $\tau \in \mathcal {T}$ of the form $\tau = [ \tau _{1}\cdots \tau _{n}]$, $\tau _{i} \in \mathcal {T}_{3}$, and $\kappa \in \mathcal {K}( \tau )$. For any $m \in {\textbf {N}}$, let $\mathcal {C}$ be an *m*-cycle in the contracted tree $\tau _{\kappa }$ and denote the inner vertices of $\mathcal {C}$ by $v_1,...,v_m$ with associated time-space coordinates $(s_{v_1},y_{v_1}),...,(s_{v_m}, y_{v_m})$. Recall the kernel $K_{\tau _{\kappa ,\varepsilon }} ( s_{\mathcal {V}}, y_{\mathcal {V}})$ from (4.5) associated to a contracted tree. Then4.16where $\widetilde{\tau } = \tau \setminus \mathcal {C}$, $\widetilde{\kappa }$ is the contraction induced by $\kappa$ on $\widetilde{\tau }$, $\mathfrak {p}(v)$ denotes the parent of a vertex *v* in $\tau$ and $\mathfrak {d}_{ \kappa }(v)$ denotes the unique descendant of *v* which is *not* part of $\mathcal {C}$.

#### Proof

The proof follows the steps of the computation in Example 3 by crucially applying Chapman–Kolmogorov to the integration over the space variables associated to the leaves of the $$\text {v}$$-cycle, combined with a uniform bound over the space variables on the resulting product of heat kernels. In particular, we havewhere we omitted integration over the time variables associated to leaves on the left-hand side, as $$ s_{w}=0$$ for all $$ w \in \mathcal {L}_{\mathcal {C}}$$, because of the Dirac–$$\delta $$ at zero. Inserting the above into the left-hand side of (4.16) we obtain the desired estimate. It is useful to have a pictorial representation of the estimate we have just performed:with the equality representing the application of Chapman–Kolmogorov, with the blue cycle appearing on the right-hand side in the first line representing the weight

$$\begin{aligned} \lambda _{\varepsilon }^{2 m} \prod _{k=1}^{m} e^{\mathfrak {m}( s_{v_{k}}+ s_{v_{k+1}})} p_{s_{v_{k}}+s_{v_{k+1}}+2\varepsilon ^2}(y_{v_{k+1}}-y_{v_k}) \,, \end{aligned}$$

and the inequality depicting the application of the uniform bound

$$\begin{aligned} \prod _{k=1}^{m} e^{\mathfrak {m}( s_{v_{k}}+ s_{v_{k+1}})}p_{s_{v_{k}}+s_{v_{k+1}}+2\varepsilon ^2}(y_{v_{k+1}}-y_{v_k}) \leqslant \prod _{k=1}^{m} e^{\mathfrak {m}( s_{v_{k}}+ s_{v_{k+1}})}p_{s_{v_{k}}+s_{v_{k+1}}+2\varepsilon ^2}(0) \,. \end{aligned}$$

The blue cycle appearing in the right-hand side in the second line represents the space-independent kernel  (hence we call this a spatial decoupling from the remaining integral). The small red nodes indicate the remaining spatial integrals associated to inner vertices of the extracted cycle. By another application of Chapman–Kolmogorov, when integrating over the spatial variables associated to the small red nodes, we obtainThis concludes the proof. $\square$

The following lemma is crucial as it demonstrates that $\text {v}$-cycles of length larger than one have a vanishing contribution, as $\varepsilon \rightarrow 0$, and in fact, the contribution is even smaller the larger the cycle is (because of the factor $\lambda _{\varepsilon }^{2m}$).

#### Lemma 4.10

The following bound holds for any $m\geqslant 1$4.17where we recall that $\overline{\mathfrak {m}} = \max \{ \mathfrak {m}\;, 0 \}$.

#### Proof

In the case $m=1$, the bound follows from (4.14), since4.18Thus, we assume $m\geqslant 2$ for the remainder of the proof. First, note that

$$\begin{aligned} s_1+s_m+2\varepsilon ^2 \geqslant 2 \sqrt{(s_1+\varepsilon ^2)(s_m+\varepsilon ^2) } \geqslant \sqrt{(s_1+2\varepsilon ^2)(s_m+2\varepsilon ^2) }\,. \end{aligned}$$

Therefore:4.19where we additionally used that $e^{\mathfrak {m} \, s_{k}} \leqslant e^{ \overline{\mathfrak {m}} \, t }$. Furthermore, for $2\leqslant k\leqslant m$, using the change of variables $r = \tfrac{s_{k}}{s_{k -1}+ 2\varepsilon ^{2}}$ together with the identity $\int _{0}^{\infty } \tfrac{1}{\sqrt{r} (1 + r)} {\,\text {d}}r = \pi$, we have that

4.20 $$\begin{aligned} \int _0^t \frac{1}{\sqrt{s_k+ 2 \varepsilon ^{2}}} \frac{ 1}{s_{k-1}+s_k+2\varepsilon ^2} {\,\text {d}}s_{k}&\leqslant \frac{ 1}{\sqrt{s_{k-1} + 2 \varepsilon ^{2}}} \int _0^{t/(s_{k-1} + 2 \varepsilon ^{2})} \frac{1}{\sqrt{r}} \frac{1}{1+r} {\,\text {d}}r \nonumber \\&\leqslant \frac{\pi }{\sqrt{s_{k-1}+2\varepsilon ^2}}\,. \end{aligned}$$

Applying (4.20) $(m-1)$-times to (4.19), starting from $k=m$ and going down to $k=2$, yieldswhich concludes the proof. $\square$

Next we want to define an iterative process of extracting cycles from a paired tree and record this process via a mapping to an element of the permutation group. We will call this the *cycle extraction map* and define it below. To define such algorithm, it will be convenient to label vertices of trees. For a given tree $\tau \in \mathcal {T}_{3}$, we fix a representative ordered version of it and label the inner vertices of $[ \tau , \tau ]$ (excluding the root) with the numbers $\{ 1, \ldots , 2i( \tau ) \}$ in arbitrary order. Once we have labeled all inner nodes, we label its leaves with the integers $\{ 2i(\tau )+1, \ldots , 4i(\tau )+2 \}$. In particular, the arguments that follow depend on the ordered structure of the trees under consideration. Yet, the eventual estimates that we obtain are uniform over all representatives of a given unordered tree, so this will not cause any problem. Now, it will be convenient to define an ordering among sequences of labels.

#### Definition 4.11

*(Lexicographic ordering)* For two vectors $\textsf {V}=(v_1,...,v_d) \in {\textbf {N}}^{d}$ and $\textsf {U}=(u_1,...,u_e) \in {\textbf {N}}^{e}$ with *d* and *e* not necessarily equal, we say that $\textsf {V}$ precedes $\textsf {U}$ in lexicographic order and write $\textsf {V} \prec \textsf {U}$ if

- either there exists a $k \leqslant d \wedge e$ such that $v_{z} = u_{z}$ for $z \leqslant k -1$ and $v_{k} < u_{k}$ ,
- or $d < e$ and $v_{z} = u_{z}$ for $z \leqslant d$.

The lexicographic order extends naturally to a total order on the set of $\text {v}$-cycles of a tree. Let $\mathcal {C}$ be a $\text {v}$-cycle, represented by the path-vector

4.21 $$\begin{aligned} \textsf {V}_{\mathcal {C}}:= (v_{i_1}, v_{j_{1}} , v_{j_{2}}, v_{i_{2}},...,v_{i_m}, v_{j_{2m-1}}, v_{j_{2m}})\,, \quad \text {such that} \quad v_{i_{k}} \in \mathcal {I}_{\mathcal {C}}\,,\ v_{j_{k}} \in \mathcal {L}_{\mathcal {C}},\nonumber \\ \end{aligned}$$

with consecutive vertices in the vector being connected by an edge in the $$\text {v}$$-cycle. Here $$i_{1}$$ is the minimal label in $$\mathcal {I}_{\mathcal {C}}$$, and $$v_{j_{1}}$$ the leaf with minimal label neighbouring $$ v_{i_{1}}$$. This imposes a direction the path-vector is represented in. Now, let $$ \mathcal {C}'$$ be a second $$\text {v}$$-cycle represented by the vector $$ \textsf {V}_{\mathcal {C}'}$$, then

4.22 $$\begin{aligned} \mathcal {C}\prec \mathcal {C}'\qquad \text {if} \qquad \textsf {V}_{\mathcal {C}} \prec \textsf {V}_{\mathcal {C}'}\ \text {lexicographically}. \end{aligned}$$

In this setting we can introduce the *cycle extraction map* $\Pi _{\tau }$, for any $\tau \in \mathcal {T}_{3}$.

#### Definition 4.12

*(Cycle extraction map)* For any $\tau \in \mathcal {T}_{3}$ and $\gamma \in \mathcal {Y}(\tau , \tau )$, we will define inductively a sequence of $\text {v}$-cycles extracted from $[\tau , \tau ]_{\gamma }$ as follows:

1. Start by defining $\sigma _{1} := [\tau , \tau ]_{\gamma }$ and $\gamma _1:=\gamma$ and denote by $\mathcal {C}_1$ the minimal $\text {v}$-cycle in $\sigma _1$ (whose existence is guaranteed by Lemma 4.7) with respect to the lexicographic order. Define $\sigma _2 :=\sigma _1 \setminus \mathcal {C}_1$ and on $\sigma _2$ the contraction $\gamma _2$ induced by $\gamma _1$ after the cycle removal of $\mathcal {C}_1$, according to Definition 4.8.
2. Assume that we have defined the contracted trees $(\sigma _i)_{\gamma _i}$, for $i=1,...,k$, as well as the $\text {v}$-cycles $\mathcal {C}_1,...,\mathcal {C}_{k-1}$ belonging respectively to $(\sigma _1)_{\gamma _1},...,(\sigma _{k-1})_{\gamma _{k-1}}$. Then proceed by defining $\mathcal {C}_k$ to be the minimal v-cycle belonging to $\sigma _k$, with respect to the lexicographic order. Further, define the contracted graph $(\sigma _{k+1})_{\gamma _{k+1}}:= (\sigma _k\setminus \mathcal {C}_k)_{{\tilde{\gamma }}_k}$ via the cycle removal as in Definition 4.8.
3. Stop at $K(\tau , \gamma ):=k$ if .

#### Definition 4.13

*(Permutation extraction map)* For $$ n \in {\textbf {N}}$$, let $$ S_{n}$$ denote the symmetric group over *n* elements, and let $$\tau \in \mathcal {T}_3$$. We define as follows the permutation extraction map

$$\begin{aligned} \begin{aligned} \Pi _{\tau } :\mathcal {Y}(\tau , \tau ) \rightarrow S_{2 i (\tau )} \;. \end{aligned} \end{aligned}$$

For any $\gamma \in \mathcal {Y}(\tau , \tau )$ consider the sequence of $\text {v}$-cycles $( \mathcal {C}_{k} )_{k =1}^{K (\tau , \gamma )}$ constructed from $[\tau ,\tau ]_\gamma$ via the cycle extraction map from Definition 4.12. For any $\mathcal {C}_k$ belonging to this sequence let $v_{i^{(k)}_1},...,v_{i^{(k)}_{m_k}}$ be the vertices in $\mathcal {I}_{\mathcal {C}_{k}}$, listed in the same order as in (4.21). We then map every cycle to a permutation cycle

$$\begin{aligned} \mathcal {C}_k \mapsto {\widehat{\mathcal {C}}}_k:= \big (i^{(k)}_1 \ i^{(k)}_2 \ \cdots \ i^{(k)}_{m_k}\big ) \in S_{2 i(\tau )}\,, \end{aligned}$$

where we used the cycle notation $$ (i^{(k)}_1 \ i^{(k)}_2 \ \cdots \ i^{(k)}_{m_k}) $$ for the permutation $$i^{(k)}_j \mapsto i^{(k)}_{j+1}$$, for $$j=1, \ldots , m_{k}$$, with $$ i^{(k)}_{m_{k}+1}=i^{(k)}_{1}$$. The image of $$ \gamma $$ under the permutation extraction map $$ \pi = \Pi _\tau (\gamma )$$ is then defined as

$$\begin{aligned} \uppi = \prod _{k=1}^{K(\tau , \gamma )} \big (i^{(k)}_1 \ i^{(k)}_2 \ \cdots \ i^{(k)}_{m_k}\big )\,. \end{aligned}$$

To clarify the tools we have introduced so far, let us discuss an example.

#### Example 4

Consider the following paired treewhere we marked the minimal $\text {v}$-cycle (with respect to lexicographic ordering) in blue, which will be removed in the first iteration of the cycle extraction. For the sake of clarity we omitted the labels of the leaves in the diagram above, and merely represent the corresponding $\text {v}$-cycles by their inner nodes. Removing the blue $\text {v}$-cycle in the diagram above yieldswhere once more we marked the new minimal $\text {v}$-cycle in blue. Removing the new blue cycle then yieldsOverall, for the above example, the permutation extraction map $\Pi _{ \tau }$ yields

4.23 $$\begin{aligned} \begin{aligned} \gamma \mapsto \big ( 1\ 3 \ 4 \big ) \big (2 \big ) \in S_{4}\,. \end{aligned} \end{aligned}$$

Some remarks on the permutation extraction map are due. First, we note that the mapping is well defined. This is because once a minimal $$\text {v}$$-cycle is to be extracted, the integers indexing its base points are removed from the permutation and the new tree has inner vertices indexed by the remaining integers. A second observation is that it is not bijective. To see that the map is not injective, consider the tree from Example 4, however, now with the pairingwhere again we marked the minimal $$\text {v}$$-cycles in blue. Then $$\Pi _{ \tau }$$ maps the above pairing also to the permutation (4.23). Moreover, the map is not surjective: For example consider a paired tree $$[ \tau , \tau ]$$ containing the componentand a permutation $$ \uppi \in S_{n}$$ containing the permutation cycle $$ \big ( 1\ 2\ 3\big )$$. Then there exists no pairing $$ \gamma \in \mathcal {Y}( \tau , \tau )$$ such that $$ \Pi _{\tau }( \gamma ) = \uppi $$, since it is not possible to construct a $$\text {v}$$-cycle according to this permutation cycle. Indeed, let us try to construct a corresponding $$\text {v}$$-cycle and see that this fails. Necessarily, $$\gamma $$ would contain an edge contracting a leaf connected to $$v_{2}$$ and $$v_{3}$$. Moreover, we can only connect a single leaf neighbouring either $$v_{2}$$ or $$ v_{3}$$ to the isolated leave neighbouring $$v_{1}$$, say we choose a leaf at $$v_2$$. Then the pairing $$ \gamma $$ would contain the following edges (in red):Now, it is not possible to close a $$\text {v}$$-cycle with $$v_3$$, while also crossing $$v_{1}, v_{2}$$. Note that this construction does not depend on the specific leaves we chose. In particular, the roles of $$v_{2}$$ and $$v_{3}$$ can be reversed.

The main result of this section is an upper bound on the integral represented by a paired tree. This bound is obtained via the permutation cycle sequence extracted by the map $\Pi _{\tau }$. The upper bound will turn out to be sharp when $\Pi _{\tau } (\gamma ) = \text {Id}$, meaning that only cycles of length one are extracted. Before we state the result, let us introduce the following notation for the time-simplex induced by a tree. For a tree $\tau \in \mathcal {T}$ we define

4.24 $$\begin{aligned} \begin{aligned} \mathfrak {D}_{ \tau }(t) := \{s \in [0,t]^{\mathcal {I}( \tau )\setminus \mathfrak {o}} \,:\, \text { if }\mathfrak {p}(u)=v ,\text { then }s_v\ge s_u \}\,, \end{aligned} \end{aligned}$$

with the usual convention that $s_{\mathfrak {o}}=t$ and $\mathfrak {p}(u)$ denoting the parent of *u*.

#### Lemma 4.14

Let $\tau \in \mathcal {T}_{3}$ and $i( \tau )$ be the number of internal vertices of $\tau$. For $\uppi \in S_{2i(\tau )}$ with permutation cycle decomposition $\{\widehat{ \mathcal {C}}_{i}\}_{i=1}^{K ( \uppi )}$ and $\gamma \in \Pi ^{-1}_\tau (\uppi )$, we have

$$\begin{aligned}{}[\tau ,\tau ]_{\gamma ,\varepsilon }(t,x)&\leqslant \lambda _\varepsilon ^2\,\Big ( \int _{\mathfrak {D}_{[\tau ,\tau ]}(t)} \Psi _{\uppi ,\varepsilon }(s_{\mathcal {I}}) {\,\text {d}}s_{\mathcal {I}}\Big ) e^{2 \mathfrak {m} \, t} p_{2(t+\varepsilon ^2)}(0)\,,\quad&\forall \pi \in S_{n},\\ [\tau ,\tau ]_{\gamma ,\varepsilon }(t,x)&= \lambda _\varepsilon ^2\,\Big ( \int _{\mathfrak {D}_{[\tau ,\tau ]}(t)} \Psi _{\uppi ,\varepsilon }(s_{\mathcal {I}}) {\,\text {d}}s_{\mathcal {I}}\Big ) e^{2 \mathfrak {m} \, t} p_{2(t+\varepsilon ^2)}(0)\,, \quad&\text { if } \pi = \text {Id} , \end{aligned}$$

where $\mathcal {I}:=\mathcal {I}([\tau ,\tau ])\setminus \mathfrak {o}$ and4.25with  defined in (4.15). Here $\mathcal {I}_{ \widehat{\mathcal {C}}}$ denotes all those inner vertices whose labels lie in the permutation cycle $\widehat{\mathcal {C}}$.

#### Proof

Let $\uppi \in S_{2i{(\tau )}}$ and $\gamma \in \Pi _\tau ^{-1}(\uppi )$. The proof works by extracting cycles successively from $[ \tau , \tau ]$ and using Lemma 4.9 to obtain a bound on $[ \tau , \tau ]_{ \gamma , \varepsilon }$ in terms of these cycles. We write $\mathcal {V}= \mathcal {V}([ \tau , \tau ])\setminus \mathfrak {o}$ and $\mathcal {I}=\mathcal {I}([\tau ,\tau ]) \setminus \mathfrak {o}$. Starting with the extraction of the minimal $\text {v}$-cycle $\mathcal {C}_1$ in $[ \tau , \tau ]_{ \gamma }$, we have4.26where $K_{([\tau , \tau ]\setminus \mathcal {C}_1)_{{\tilde{\gamma }}}}^{t,x}$ denotes the kernel corresponding to the fully contracted tree $([\tau , \tau ]\setminus \mathcal {C}_1)_{{\tilde{\gamma }}}= (\sigma _{1})_{ {\tilde{\gamma }}}$, following the notation in Definition 4.8, and $\mathfrak {d}_{\tau , \gamma }(v)$ indicates the unique descendant of $v \in \mathcal {I}_{\mathcal {C}_{1}}$ not in $\mathcal {C}_{1}$ (see also Lemma 4.9). We can then proceed iteratively by extracting the $\text {v}$-cycles via the cycle extraction map from Definition 4.12, until we reach the tree . In this way we obtain the upper bound, using Lemma 4.9,4.27with the sequence of $\text {v}$-cycles $( \mathcal {C}_{k} )_{k =1}^{K (\tau , \gamma )}$ and the sequence of reduced trees $(\sigma _{i})_{i=1}^{K ( \tau , \gamma )}$ from the cycle extraction map. Note that $K ( \tau , \gamma )$ equals the number of permutation cycles $K (\uppi )$. In order to avoid confusion, we added a subindex $\mathfrak {p}_{ \sigma _{i}}$ to the parent map $\mathfrak {p}$ (and also to the descendant map $\mathfrak {d}_{\gamma }$), making clear with respect to which tree the map is to be interpreted. In Lemma A.5, we will see that the time-integral over the indicator functions in (4.27) preserves the original ordering imposed by the tree. More precisely, it is independent of the chosen pairing $\gamma$ and equals the integral over the tree simplex $\mathfrak {D}_{[ \tau , \tau ]}(t)$. Thus, by application of Lemma A.5, the inequality (4.27) readswhich yields the desired upper bound. If $\uppi = \text {Id}$, then the inequality in the first line becomes an equality as we are successively removing 1-cycles and apply the identity in Lemma 4.5, rather than the upper bound in Lemma 4.9. The proof is complete. $\square$

## Contributing and non-contributing trees and their structure

This section is dedicated to the proof of Proposition 3.3. Obtaining this result requires a precise quantitative control over the limiting behavior of contracted and paired trees. Such control will build on a systematic application of the bounds and ideas that we have introduced in Sect. 4, and in particular it will build on Lemma 4.14 above.

In the previous section we analysed *paired* trees and found that it was possible to identify $\text {v}$-cycles and remove them iteratively to obtain an upper bound (or an exact estimate, in the case when all $\text {v}$-cycles are 1-cycles) on the integral associated to such a tree. In this section we start instead with an arbitrary *contracted* ternary tree $[\tau ]_{\kappa }$. Our objective is to obtain a bound (or an exact estimate) on the second moment of the Wiener integral associated to such contracted tree. To obtain such estimate, we must sum over all possible pairings $\gamma$ of $[\tau , \tau ]$ which complete the contraction $\kappa$, and for each such pairing we can follow the procedure described in the previous section. One of the key points of this section is therefore to keep track of all the combinatorial factors that appear when counting pairings and contractions associated to arbitrarily large trees.

This section will be split into two parts. First, we study those contracted trees that do not vanish in the limit $\varepsilon \rightarrow 0$ (i.e. that contribute) and identify them using properties of the underlying graph $\tau _{ \kappa }$. We also determine their precise limiting contribution and the size of the set of all such contractions. In the second part, we will instead state and prove a uniform upper bound for the rate of convergence of contracted trees that vanish as $\varepsilon \rightarrow 0$ (i.e. that do not contribute).

### Contributing contractions

We start by studying those contracted trees that contribute to the fluctuations in the limit $\varepsilon \rightarrow 0$, and for which an exact estimate of the contribution is necessary. For this reason, let us define *contributing contractions* as follows.

#### Definition 5.1

For any tree $\tau \in \mathcal {T}_3$ and contraction $\kappa \in \mathcal {K}(\tau )$, we say that $\kappa$ *contributes* if there exist $(t,x) \in (0, \infty ) \times {\textbf {R}}^{2}$ such that

5.1 $$\begin{aligned} \begin{aligned} \limsup _{\varepsilon \rightarrow 0} \; (\log \tfrac{1}{\varepsilon }) \cdot \mathbb {E}\Big [\big |[\tau ]_{\varepsilon , \kappa } (t, x)\big |^2\Big ] >0\,. \end{aligned} \end{aligned}$$

We denote the set of all contributing contractions by

$$\begin{aligned} C(\tau ):= \{\kappa \text { such that } \tau _\kappa \text { contributes}\} \subseteq \mathcal {K}(\tau )\,. \end{aligned}$$

#### Identifying contributing contractions

Before determining the precise contribution of contracted trees, we show that the abstract condition in (5.1) can be replaced by a condition on the underlying graph structure of the contracted tree. More precisely, we will see that the estimates of Sect. 4.3 imply that contracted trees contribute if and only if the corresponding integrals lie in the first homogeneous Wiener chaos and we can iteratively remove 1-cycles from them.

##### Lemma 5.2

Let $$ \tau \in \mathcal {T}_{3}$$, then

(i) $$ C ( \tau ) = \{ \kappa \in \mathcal {K}( \tau )\,:\, \exists \gamma \in \mathcal {Y}( \tau _{\kappa }, \tau _{ \kappa }) \text { with } \Pi _{ \tau }( \gamma ) = \text {Id}\}$$.
(ii) If $\kappa \in C( \tau )$, then $\tau _{\kappa }$ has a single uncontracted leaf. That is, $[ \tau ]_{\kappa }$ lies in the first Wiener chaos and is therefore Gaussian.

We postpone the proof of the lemma to the end of this section. The property of being contributing, associated to a contracted tree $[\tau ]_{\kappa }$, is defined using the second moment condition (5.1). We can express (5.1) by summing $[\tau , \tau ]_{\gamma }$ over all pairings in $\gamma \in \mathcal {Y}( \tau _{\kappa }, \tau _{\kappa })$, recall (4.8). The paired tree obtained, once we fix an element of $\mathcal {Y}( \tau _{\kappa }, \tau _{\kappa })$, can be treated via Lemma 4.14. In particular, it turns out that for contributing trees $\tau _{ \kappa }$ and $\gamma \in \mathcal {Y}( \tau _{\kappa }, \tau _{\kappa })$ the cycle extraction map satisfies $\Pi _{\tau } (\gamma ) = \text {Id}$. As a consequence, we can determine precisely the limiting behavior of such paired trees through the last statement of Lemma 4.14, which is the key ingredient in the proof of Lemma 5.2. This is the content of the following lemma.

##### Lemma 5.3

Let $\tau \in \mathcal {T}_{3}$,  be the trimmed tree, as in (2.18), and $\uppi \in S_{2i(\tau )}$. Then for every $\gamma \in \Pi _{ \tau }^{-1}(\uppi )$ and all $(t,x) \in (0, \infty ) \times {\textbf {R}}^{2}$If the above limit vanishes, we say $\gamma$ is a *non-contributing pairing*, and call it a *contributing pairing* otherwise.

The proof of Lemma 5.3 uses the following identity.

##### Lemma 5.4

Let $\tau \in \mathcal {T}_{3}$ and  be the trimmed tree as in (2.18), then5.2with the tree-time-simplex $\mathfrak {D}_{ [\tau ]}(t)$ introduced in (4.24).

This lemma is a consequence of Lemma A.3, since the integrand on the left-hand side in (5.2) is a symmetric function over the variables .

##### Proof of Lemma 5.3

Consider $\tau \in \mathcal {T}_{3}$, $\uppi \in S_{2i(\tau )}$ and $\gamma \in \Pi _{\tau }^{-1}(\uppi )$. Then for all $(t,x) \in (0,\infty ) \times {\textbf {R}}^2$, Lemma 4.14 implies

$$\begin{aligned} (\log \tfrac{1}{\varepsilon }) \cdot [\tau ,\tau ]_{\gamma ,\varepsilon }(t,x) \leqslant \hat{\lambda }^2\,\left\{ \int _{\mathfrak {D}_{ [\tau ,\tau ]}(t)} \Psi _{\uppi ,\varepsilon }(s) {\,\text {d}}s_{\mathcal {I}} \right\} e^{2 \mathfrak {m} \, t} p_{2(t+\varepsilon ^2)}(0) \,, \end{aligned}$$

where we remind that $\mathcal {I}:=\mathcal {I}([\tau ,\tau ])\setminus \mathfrak {o}$. Extending the domain of integration from $\mathfrak {D}_{ [\tau ,\tau ]}(t)$ to $[0,t]^{\mathcal {I}}$, the right-hand side can be factorisedwhere $({\mathcal {C}}_i )_{i=1}^{K ( \tau , \gamma )}$ denotes the sequence of $\text {v}$-cycles constructed from $[\tau ,\tau ]_\gamma$ via the cycle extraction map (Definition 4.12). For each of the integrals we havewhere for the case $| \mathcal {I}_{\mathcal {C}_i}| \geqslant 2$ we used Lemma 4.10. Note that the right-hand side in the second case vanishes in the limit $\varepsilon \rightarrow 0$, since $\lambda _{\varepsilon } \sim (\log {\tfrac{1}{\varepsilon }})^{- 1/2}$. In particular, if $\uppi \ne \text { Id}$ then at least one $\text {v}$-cycle ${\mathcal {C}}_i$ must satisfy $|\mathcal {I}_{\mathcal {C}_i}| \geqslant 2$, which yields that $[ \tau , \tau ]_{ \gamma , \varepsilon }(t,x)$ vanishes as $\varepsilon \rightarrow 0$.

On the other hand, if $\uppi = \text {Id}$ all the $\text {v}$-cycles $\mathcal {C}_i$ are 1-cycles and we can replace all inequalities with identities to obtainHence, we deduce from Lemma 5.4 and Lemma A.1 thatwhich concludes the proof. $\square$

Finally, we are ready to show that contributing contractions can be identified as those that have a single uncontracted leaf and allow for an iterative removal of 1-cycles.

##### Proof of Lemma 5.2

Let us start by recalling from (4.8) that for any tree $\tau \in \mathcal {T}_{3}$ and any contraction $\kappa \in \mathcal {K}(\tau )$

$$\begin{aligned} \begin{aligned} (\log \tfrac{1}{\varepsilon }) \cdot \,\mathbb {E}\left[ [\tau ]_{\kappa ,\varepsilon }^2 (t, x)\right] = \sum _{\gamma \in \mathcal {Y}(\tau _{\kappa }, \tau _{\kappa })} (\log \tfrac{1}{\varepsilon }) \cdot [ \tau ,\tau ]_{\gamma , \varepsilon } (t, x) \,. \end{aligned} \end{aligned}$$

Therefore, to prove the first statement of the lemma, it suffices to prove that

$$\begin{aligned} \lim _{\varepsilon \rightarrow 0}(\log \tfrac{1}{\varepsilon }) \cdot [ \tau ,\tau ]_{\gamma , \varepsilon }(t, x) >0\,, \end{aligned}$$

for all $(t, x) \in (0, \infty ) \times {\textbf {R}}^{2}$ if and only if $\Pi _{\tau } (\gamma ) = \text {Id}$, which is implied by Lemma 5.3.

For the second statement we instead proceed by induction over the number of inner vertices $i( \tau )$. We can check that the statement is true for $i( \tau )=0$, i.e. , since $| \mathcal {Y}( \tau , \tau )| = 1$ andNow, let $m \in {\textbf {N}}$ and assume that the statement holds for all $\tau ' \in \mathcal {T}_{3}$ satisfying $i ( \tau ') \leqslant m$. Choose $\tau \in \mathcal {T}_{ 3}$ with $i ( \tau ) = m+1$ and let $\kappa \in C( \tau )$. By the first point of the present Lemma 5.2, which we have just proven, we know there exists a $\gamma \in \mathcal {Y}( \tau _{\kappa }, \tau _{\kappa }) \cap \Pi _{ \tau }^{-1}( \text {Id})$. In particular, let $\mathcal {C}_{1}$ be the first 1-cycle that is extracted by the permutation-extraction map applied to the pairing $\gamma$, and write $(\sigma _{2})_{ \gamma _{2}}= ([\tau , \tau ]\setminus \mathcal {C}_1)_{ \gamma _{2}} = [ \hat{\tau }, \tau ]_{\gamma _{2}}$, with $\hat{\tau }:= \tau \setminus \mathcal {C}_{1}$, assuming without loss of generality that we have removed the cycle from the left tree.

Then via (4.26), we deduce that5.3where we again used $\mathcal {V}= \mathcal {V}([ \tau , \tau ]) \setminus \mathfrak {o}$. Note that the product in the expression above only consists of a single term, because $\mathcal {C}_{1}$ is a 1-cycle. Dropping the time-constraint encoded by $\mathbb {1}_{ \mathfrak {d}_{ \gamma }( v) \leqslant s_v \leqslant s_{ \mathfrak {p}(v)}}$, we therefore obtain5.4Now, taking the $\limsup$ over $\varepsilon \rightarrow 0$ after multiplying both sides with $(\log { \tfrac{1}{\varepsilon }})$ yields

5.5 $$\begin{aligned} \begin{aligned} 0< \limsup _{ \varepsilon \rightarrow 0} \ (\log \tfrac{1}{\varepsilon }) \,[\tau ,\tau ]_{\gamma , \varepsilon }(t,x) \leqslant \frac{ \hat{\lambda }^{2}}{2 \pi } \limsup _{ \varepsilon \rightarrow 0} \ (\log \tfrac{1}{\varepsilon }) \, [ \hat{\tau }, \tau ]_{\gamma _{2}, \varepsilon }(t, x) \,. \end{aligned} \end{aligned}$$

Here, the first inequality holds since $\kappa \in C(\tau )$, while the second inequality is a consequence of (5.4) and Lemma A.1. In particular, the limit on the right-hand side must be positive. Next, by Definition 4.3 there exists $( \kappa _{1}( \gamma _{2}), \kappa _{2}( \gamma _{2}))$ such that $\gamma _{2} \in \mathcal {Y}( \hat{\tau }_{\kappa _{1}( \gamma _{2})}, \tau _{\kappa _{2}(\gamma _{2})})$, with $\kappa _2 ( \gamma _{2}) = \kappa$, so via an application of the Cauchy–Schwartz inequality we obtain

5.6 $$\begin{aligned} \begin{aligned} {[} \hat{\tau }, \tau ]_{\gamma _{2}, \varepsilon } \leqslant \mathbb {E}\left[ {[}\hat{\tau }]_{\kappa _{1}(\gamma _{2}),\varepsilon }\, [\tau ]_{\kappa , \varepsilon }\right] \leqslant \left( \mathbb {E}\left[ {[}\hat{\tau }]_{\kappa _{1}(\gamma _{2}),\varepsilon }^2\right] \right) ^{ \frac{1}{2}} \left( \mathbb {E}\left[ [\tau ]_{\kappa ,\varepsilon }^2\right] \right) ^{ \frac{1}{2}} \,, \end{aligned} \end{aligned}$$

which together with (5.5) implies $\kappa _{1}( \gamma _{2}) \in C( \hat{\tau })$. By the induction assumption, $\hat{\tau }_{\kappa _1( \gamma _2)}$ (note that $i(\hat{\tau })=m$) has a single uncontracted leaf, which implies that also $\tau _{\kappa }$ has a single uncontracted leaf. This concludes the proof. $\square$

Note that Lemma 5.2, together with the identity from Lemma 5.3, implies that if $\kappa \in C (\tau )$ then $[\tau ]_{\varepsilon , \kappa }$ is (and converges after rescaling to) a mean-zero Gaussian, with limiting fluctuationsIn the next subsection, we will see that a stronger statement holds true, as we will be able to identify $[ \tau ]_{\kappa , \varepsilon }$ with  up to a multiplicative factor.

#### Determining contributions

In the previous section, we identified contributing pairings (and contractions) to be the ones mapped by $\Pi _{ \tau }$ to the identity permutation, i.e. the algorithm defined in Definition 4.12 only extracts cycles of length one. Precisely this fact will turn out to be useful, when determining the following identity for contributing contracted trees.

##### Lemma 5.5

For every $\tau \in \mathcal {T}_{3}, \kappa \in {C}( \tau )$, $\varepsilon \in (0,\tfrac{1}{2})$ and $(t,x) \in (0, \infty ) \times {\textbf {R}}^{2}$ we have that

Note that the right-hand side of the identity in the lemma above does not depend on the particular contraction $\kappa \in C(\tau )$.

##### Proof

Fix $$ \tau \in \mathcal {T}_{3}$$ and let $$ \kappa \in {C}( \tau ) $$. Choose $$ \gamma \in \Pi _{ \tau }^{-1}(\text {Id}) \cap \mathcal {Y}( \tau _{ \kappa }, \tau _{ \kappa } )$$, which exists by Lemma 5.2(i). Since $$ \gamma \in \Pi _{\tau }^{-1} (\text {Id}) $$, the cycle extraction map, see Definition 4.12, associates to $$ \gamma $$ a sequence of 1-cycles $$(\mathcal {C}_i)_{i=1}^{2 i (\tau )}$$. In order to distinguish between the two trees generating $$[ \tau , \tau ]$$, let us write $$[ \tau _{1}, \tau _{2}]:= [\tau , \tau ]$$. Now, let $$( \mathcal {C}_{i} ')_{i=1}^{ i ( \tau )}$$ be the subset of cycles whose bases belong to the tree $$ \tau _{1} $$. In other words, $$( \mathcal {C}_{i} ')_{i=1}^{ i ( \tau )}$$ contains all cycles in $$(\mathcal {C}_i)_{i=1}^{2 i (\tau )}$$ such that $$\mathcal {I}_{\mathcal {C}_{i}}\subset \mathcal {I}( \tau _{1})$$. This yields an iterative rule of removing 1-cycles from $$ [\tau _{1}]_{ \kappa }=[ \tau ]_{\kappa }$$. In particular, each $$ {\mathcal {C}}_{i}$$ corresponds to a unique inner node $$ v_i$$ of $$ \tau $$.

Recall that for $v \in \mathcal {I}(\tau )$ we write $\mathfrak {p}(v) \in \mathcal {I}([\tau ])$ for the parent of *v* and recall also the representation of the stochastic integrals in (4.4), which allows us to write

$$\begin{aligned} \begin{aligned} {[} \tau ]_{ \kappa , \varepsilon }(t,x)&= \int _{D_{t}^{\mathcal {V}(\tau )}} K_{[\tau _{\kappa }]}^{t, x} ( s_{\mathcal {V}},y_{\mathcal {V}})\; {\,\text {d}}s_{\mathcal {V}( \tau )} \, {\,\text {d}}y_{\mathcal {V}(\tau )\setminus \ell }\ \eta _\varepsilon ( {\,\text {d}}y_{\ell }) \;, \end{aligned} \end{aligned}$$

where we denote by $(s_{ \ell } ,y_{ \ell })$ the space-time point associated to the single uncontracted leaf in $\tau _{ \kappa }$, cf. Lemma 5.2(ii). We apply Lemma 4.5 with respect to the 1-cycle $\mathcal {C}^{\prime }_{1}$, which yieldsNow, by applying Lemma 4.5 successively another $i (\tau )-1$ times with respect to each of the 1-cycles $( \mathcal {C}_{i} ')_{i=2}^{ i ( \tau )}$, we obtainThe stochastic integral on the right-hand side equals , whereas the time integral in the brackets can be rewritten aswhere we used that

$$\begin{aligned} \begin{aligned} \prod _{v \in \mathcal {I}(\tau )} \mathbb {1}_{\{ s_{\mathfrak {d}_\kappa (v)} \leqslant s_{v} \leqslant s_{ \mathfrak {p}(v)} \}} = \prod _{v \in \mathcal {I}(\tau )} \mathbb {1}_{\{ s_{v} \leqslant s_{ \mathfrak {p}(v)} \}}\;. \end{aligned} \end{aligned}$$

Together with Lemma 5.4, this concludes the proof. $\square$

#### Counting contributing contractions

In the previous section, we saw that the limit of a contributing contracted tree is independent of the precise structure of the contraction. Thus, in order to conclude the limit of $X^{ \tau }$, it is only left to determine the size of $C( \tau )$.

##### Lemma 5.6

Let $$ \tau \in \mathcal {T}_{3}$$, then $$|{C}( \tau )| = 3^{ i( \tau )}$$.

In order to prove Lemma 5.6, we first need the following result.

##### Lemma 5.7

Let $\tau \in \mathcal {T}_{3}$ and $\kappa \in C( \tau )$, then every trident in $\tau _\kappa$ has an internal contraction. More precisely, for every , withwe have $\{u_{i}, u_{j}\} \in \kappa$ for two distinct $i,j \in \{ 1,2,3\}$.

##### Proof

Let $\tau \in \mathcal {T}_{3}$, $\kappa \in C( \tau )$ and consider $[ \tau , \tau ]_{\gamma }$ for the unique $\gamma \in \mathcal {Y}( \tau _{\kappa }, \tau _{\kappa })$, see Lemma 5.2(ii). For any , we write $u_{1}(v), u_{2}(v), u_{3}(v) \in \mathcal {L}( \tau )$ for the three leaves it is connected to (indexing them with 1 to 3 from left to right).

Now, assume there exists a  without an internal contraction, i.e. $\{u_{i}(v), u_{j}(v)\} \notin \kappa$ for all $1 \leqslant i,j \leqslant 3$. Graphically, this can be represented as follows:5.7for some $\tau _{1}, \tau _{2} \in \mathcal {T}_{3}$ (note that possibly one of the leaves could be uncontracted, which is indicated in the example above by the dotted red line). Then it is immediate to see that $\Pi _{\tau }( \gamma ) \ne \text {Id}$, since otherwise $\{u_{i}(v), u_{j}(v)\} \in \kappa \subset \gamma$ for some $1 \leqslant i,j \leqslant 3$. Thus, contradicting the assumption $\kappa \in C( \tau )$ by Lemma 5.2(i). This concludes the proof. $\square$

##### Proof of Lemma 5.6

We prove the statement by induction over the number of inner vertices $i ( \tau )$, starting with $i( \tau ) =0$, i.e. . In this case, $|C( \tau ) | =|\mathcal {K}( \tau )|= |\mathcal {Y}( \tau , \tau )|=1$ and the claim holds. Now assume the statement holds true for any $\widehat{\tau } \in \mathcal {T}_{3}$ satisfying $i ( \widehat{\tau }) \leqslant m$.

Let $\tau \in \mathcal {T}_{3}$ with $i( \tau ) =m+1$ and fix any . We denote its neighbouring leaves by $u_{1}(v), u_{2}(v), u_{3}(v) \in \mathcal {L}( \tau )$ (indexing them with 1 to 3 from left to right):5.8for some $\tau _{1}, \tau _{2} \in \mathcal {T}_{3}$. Again note that possibly one of the leaves could be uncontracted. Moreover, using Lemma 5.7, we can partition $C( \tau )$ into three sets, $C_{1,2}( \tau ), C_{1,3}( \tau ), C_{2,3}( \tau )$, with

$$\begin{aligned} \begin{aligned} C_{i,j}( \tau ):= \left\{ \kappa \in C ( \tau ) \,:\, \{u_{i}(v), u_{j}(v) \} \in \kappa \right\} \,. \end{aligned} \end{aligned}$$

For any contraction $\kappa \in C( \tau )$ we define the tree resulting from $\tau _{\kappa }$ after removing the 1-cycle $\mathcal {C}$ with $\mathcal {I}_{\mathcal {C}}= \{v\}$:

5.9 $$\begin{aligned} \begin{aligned} \widehat{\tau }_{ \tilde{\kappa }}:= (\tau \setminus \mathcal {C})_{\tilde{ \kappa }}, \end{aligned} \end{aligned}$$

using the cycle removal from Definition 4.8. Expression (5.9) defines a map $\mathfrak {K}_{v} : C( \tau ) \rightarrow \mathcal {K}( \widehat{ \tau })$ with $\mathfrak {K}_{v} ( \kappa ):= \tilde{ \kappa }$. Moreover, we have that $\widetilde{\kappa }$ is contributing for $\hat{\tau }$ (namely $\tilde{ \kappa }=\mathfrak {K}_{v} ( \kappa ) \in C ( \hat{\tau })$).

In fact, for any choice $1 \leqslant i< j \leqslant 3$, the map $\mathfrak {K}_{v}|_{C_{i,j}( \tau )}$ maps onto $C( \widehat{\tau } )$ and defines a bijection. To see this, consider an arbitrary contraction $\widehat{ \kappa } \in C ( \widehat{ \tau })$ (with the labeling of $\widehat{ \tau }$ induced by $\tau$) and define $\kappa := \widehat{ \kappa } \cup \{u_{i}(v), u_{j}(v)\}$. For example we have the following reconstruction of a contraction in $C_{2,3}( \tau )$ using the inverse $(\mathfrak {K}_{v}|_{C_{2,3}( \tau )})^{-1}$:5.10On the other hand, for the same $\widehat{ \kappa }$ we can also reconstruct the following two contractions in $C_{1,2}( \tau )$ and $C_{1,3}( \tau )$ , respectively:In particular, for each set $C_{i,j}( \tau )$ there exists a unique $\kappa \in C_{i,j}( \tau )$ such that $\mathfrak {K}_{v}(\kappa ) = \widehat{\kappa }$. As a consequence all three sets $C_{i,j}( \tau )$ have the same cardinality, which agrees with $|C ( \widehat{ \tau })|$. Lastly, applying the induction hypothesis to $|C ( \widehat{ \tau })|$, yields

$$\begin{aligned} \begin{aligned} |C ( \tau )| = |C_{1,2}( \tau )|+ |C_{1,3}( \tau )|+ |C_{2,3}( \tau )| = 3 |C( \widehat{\tau })| = 3^{m+1}\,. \end{aligned} \end{aligned}$$

This concludes the proof. $\square$

### Non-contributing trees

Up to now, we have identified contributing pairings (and contractions) to be the ones that lie in the pre-image $\Pi _{\tau }^{-1}(\text {Id})$, when considering a fixed tree $\tau \in \mathcal {T}_{3}$ . Moreover, we determined their exact contribution. Now, it is only left to control the overall contribution of the remaining contractions, which we will prove to be negligible, in a strong summable fashion. We summarise the main findings of this section in the following lemma.

#### Lemma 5.8

Let $T>0$, then uniformly over any $\varepsilon \in (0, \tfrac{1}{T} \wedge \tfrac{1}{2})$, $\tau \in \mathcal {T}_{3}^{N_{\varepsilon }}$, for $N_{\varepsilon } = \lfloor \log {\tfrac{1}{\varepsilon }}\rfloor$, $x \in {\textbf {R}}^{2}$ and uniformly for all $t \in [0, T]$, we havewhere  denotes the trimmed tree $\mathscr {T} ( \tau )$ as in (2.18). In particular, for a fixed $\tau \in \mathcal {T}_3$ the right-hand side vanishes in the small-$\varepsilon$ limit.

#### Remark 5.9

Our methods in Sect. 4.3 (such as the cycle extraction and the corresponding estimates in Lemma 4.14) also apply to covariances of the form

$$\begin{aligned} \begin{aligned} \left( \log {\tfrac{1}{\varepsilon }}\right) \cdot \sum _{\kappa , \kappa ' \notin C(\tau )} \mathbb {E}\big [ [\tau ]_{\kappa ,\varepsilon }(t,x) {[}\tau ]_{\kappa ,\varepsilon }(t',x') \big ]\,, \end{aligned} \end{aligned}$$

instead of just second moments as considered in Lemma 5.8. For this we don’t identify the roots of the trees $[ \tau ]_{ \kappa }$ and $[ \tau ]_{ \kappa '}$, when pairing the two trees, but keep them separate with individual time-space points associated. Thus, instead of stopping the cycle extraction (Definition 4.12) once we see , we terminate the algorithm once  appears. For this reason, we would see a $\sqrt{ \pi e^{2\mathfrak {m}\, t} p_{2(t+ \varepsilon ^{2})}(x-x')}$ instead of

$$\begin{aligned} \begin{aligned} \frac{ e^{ \mathfrak {m}\, t}}{\sqrt{4 (t + \varepsilon ^{2})}} = \sqrt{ \pi e^{2\mathfrak {m}\, t} p_{2(t+ \varepsilon ^{2})}(0)}\,, \end{aligned} \end{aligned}$$

on the right-hand side of Lemma 5.8. In particular, we expect this to allow for treatment of the statistics of the corresponding field associated to (1.3) in the Hölder space $\mathcal {C}^{+1 -}({\textbf {R}}^{2})$ for fixed $t>0$, which we leave for future work.

For the proof of Lemma 5.8 we need the following two lemmas. The first is an upper bound of a “symmetrised” integral over v-cycles.

#### Lemma 5.10

Let $T>0$. Then uniformly over any $\varepsilon \in (0, \tfrac{1}{T} \wedge \tfrac{1}{2})$, $t \in [0, T]$ and $\tau \in \mathcal {T}_{3}^{N_{\varepsilon }}$, $N_{\varepsilon } = \lfloor \log {\tfrac{1}{\varepsilon }}\rfloor$, we havewhere $\mathcal {I}:= \mathcal {I}([ \tau , \tau ]) \setminus \mathfrak {o}$, and  denotes the trimmed tree (2.18). Recall (4.25) for the definition of the function $\Psi _{\uppi ,\varepsilon }$.

The next result guarantees that the number of pairings $\gamma$ of a tree $[\tau , \tau ]$, which correspond to a permutation $\uppi \in S_{2 i (\tau )}$, grows at most exponentially in the number of inner vertices of a tree.

#### Lemma 5.11

Let $\tau \in \mathcal {T}_{3}$ and $\uppi \in S_{2 i ( \tau )}$, then $\left| \Pi _{ \tau }^{-1} ( \uppi ) \right| \leqslant 6^{2 i (\tau )}$.

Having both Lemma 5.10 and 5.11 at hand, we can now prove Lemma 5.8. The proofs of Lemma 5.10 and 5.11 are deferred to the end of this section.

#### Proof of Lemma 5.8

Consider $T>0$, $\varepsilon \in (0, \tfrac{1}{T} \wedge \tfrac{1}{2})$ and $(t,x) \in [0, T]\times {\textbf {R}}^{2}$. By representing second moments of contracted trees in terms of paired trees, cf. (4.8), we have

$$\begin{aligned} \begin{aligned} \left\| \sum _{\kappa \notin C(\tau )}\sqrt{\log {\tfrac{1}{\varepsilon }}} \cdot {[}\tau ]_{\kappa ,\varepsilon }(t,x)\right\| ^{2}_{L^2(\mathbb {P})}&= \log {\tfrac{1}{\varepsilon }} \sum _{\begin{array}{c} \kappa , \kappa ' \notin C(\tau ) \end{array}} \sum _{ \gamma \in \mathcal {Y}( \tau _{ \kappa }, \tau _{ \kappa '})} {[}\tau , \tau ]_{ \gamma , \varepsilon }(t,x)\\&\leqslant \log {\tfrac{1}{\varepsilon }} \sum _{\uppi \in S_{2 i( \tau )}\setminus \{\text {Id}\}} \sum _{\gamma \in \Pi _\tau ^{-1}(\uppi )} [\tau ,\tau ]_{\gamma ,\varepsilon }(t,x)\,, \end{aligned} \end{aligned}$$

where we used additionally Lemma 5.3 to identify non-contributing pairings as precisely the ones that do not map onto $\text {Id}$ under $\Pi _{ \tau }$^6^, and the fact that

$$\begin{aligned} \begin{aligned} \exists \gamma \in \mathcal {Y}( \tau _{ \kappa }, \tau _{ \kappa '}) \quad \limsup _{ \varepsilon \rightarrow 0}\, \left( \log { \tfrac{1}{\varepsilon }} \right) [ \tau , \tau ]_{ \gamma , \varepsilon }(t,x) >0 \quad \Leftrightarrow \quad \kappa , \kappa ' \in C ( \tau )\,, \end{aligned} \end{aligned}$$

which is a consequence of the Cauchy–Schwartz inequality, cf. (5.6), and Lemma 5.2. Thus, Lemmas 4.14 and 5.11 imply

$$\begin{aligned} \begin{aligned}&\Bigg \Vert \sum _{\kappa \notin C(\tau )}\sqrt{\log {\tfrac{1}{\varepsilon }}} \cdot [\tau ]_{\kappa ,\varepsilon }(t,x)\Bigg \Vert ^{2}_{L^2(\mathbb {P})} \\&\quad \leqslant \left( \log {\tfrac{1}{\varepsilon }}\right) \, \lambda _\varepsilon ^2\, 6^{2 i ( \tau )} \sum _{\uppi \in S_{2 i ( \tau )}\setminus \{\text {Id}\}} \Big ( \int _{\mathfrak {D}_{ [ \tau ,\tau ]}(t)} \Psi _{\uppi ,\varepsilon }(s_{\mathcal {I}}) {\,\text {d}}s_{\mathcal {I}} \Big ) \, e^{2 \mathfrak {m}\, t} p_{2(t+\varepsilon ^2)}(0) \,. \end{aligned} \end{aligned}$$

Applying Lemma 5.10, this can be further upper bounded bySince , the result follows by taking the square root. $\square$

Now we pass to the proof of Lemma 5.10. Note that this is an improvement of Lemma 5.3. Indeed, instead of extending the integration domain from $\mathfrak {D}_{ [\tau , \tau ]}(t)$ to the box $[0,t]^{\mathcal {I}([ \tau , \tau ])\setminus \mathfrak {o} }$, as it was done in the proof of the latter lemma, we will make use of the fact that summation over all permutations in $\uppi \in S_{n}\setminus \{\text {Id}\}$ has a symmetrising effect that allows for a more precise control of the integral.

#### Lemma 5.12

Fix $\varepsilon \in (0,\tfrac{1}{2})$ and $n \in {\textbf {N}}$. Consider for any $\uppi \in S_{n}$ the function $\Psi _{\uppi ,\varepsilon }$ introduced in (4.25). Then the function $\overline{\Psi }_\varepsilon : [0, \infty )^{n} \rightarrow {\textbf {R}}$ defined by

$$\begin{aligned} \overline{\Psi }_\varepsilon (s_{1}, \ldots s_{n}) := \sum _{\uppi \in S_{n}\setminus \{\text {Id}\}} \Psi _{\uppi ,\varepsilon }(s_{1}, \ldots , s_{n})\,, \end{aligned}$$

is symmetric in the variables $s_{1}, \cdots , s_{n}$.

#### Proof

It suffices to consider the function $\overline{\varphi }_{ \varepsilon }$ given by

$$\begin{aligned} (s_1,...,s_n) \mapsto \sum _{\uppi \in S_{n}} \prod _{i=1}^{n} \frac{1}{s_i+s_{\uppi (i)}+2\varepsilon ^2}\,, \end{aligned}$$

since the term corresponding to the identity partition is symmetric itself and we have

$$\begin{aligned} \overline{\Psi }_\varepsilon = \left( \frac{ \lambda _{ \varepsilon }^{2}}{2 \pi } \right) ^{n} e^{2\mathfrak {m} \sum _{i =1}^{n} s_{i} } \left( \overline{\varphi }_{ \varepsilon } - \prod _{i=1}^{n} \frac{1}{2 s_i+2\varepsilon ^2}\right) \;. \end{aligned}$$

Now, for ${\upsigma }\in S_{n}$, if we indicate $s_{\upsigma } = (s_{\upsigma (i) })_{i=1}^{n}$, we have

$$\begin{aligned} \overline{\varphi }_{ \varepsilon }(s_\upsigma )&= \sum _{\uppi \in S_{n}} \prod _{i=1}^{n} \frac{1}{s_{\upsigma (i)}+s_{\upsigma (\uppi (i))}+2 \varepsilon ^{2}} = \sum _{\uppi \in S_{n}} \prod _{i=1}^{n} \frac{1}{s_{i}+s_{\upsigma \uppi \upsigma ^{-1}(i)}+2 \varepsilon ^{2}}\\&= \sum _{\uppi \in S_{n}} \prod _{i=1}^{n} \frac{1}{s_{i}+s_{\uppi (i)}+2 \varepsilon ^{2}} = \overline{\varphi }_{ \varepsilon }(s)\,, \end{aligned}$$

since for every $\sigma \in S_{n}$ we have $\{ \upsigma \uppi \upsigma ^{-1}\, :\, \uppi \in S_{n}\}= S_{n}$. This concludes the proof. $\square$

Now we are ready to prove Lemma 5.10.

#### Proof of Lemma 5.10

Fix $T>0$, $t \in [0, T]$ and define $n:= 2i ( \tau )$. Using Lemma A.3 and the definition of $\overline{\Psi }_{\varepsilon }$ in Lemma 5.12, we find that5.11since $\overline{\Psi }_\varepsilon$ is symmetric by Lemma 5.12. Recall from (4.25) that $\Psi _{\uppi ,\varepsilon }$ is a product over cycles in the permutation $\uppi$, which allows us to factorise the integralwith $K ( \uppi )$ denoting the number of cycles in the permutation $\uppi$. Applying Lemma 4.10 to each term in the product yields

$$\begin{aligned} \begin{aligned} \int _{[0,t]^{\mathcal {I}}} \Psi _{\uppi ,\varepsilon }(s_{\mathcal {I}}) {\,\text {d}}s_{\mathcal {I}}&\leqslant \prod _{i=1}^{K ( \uppi )} \left( \frac{(\lambda _\varepsilon e^{ \overline{\mathfrak {m}}\, t})^{2|\widehat{\mathcal {C}}_i|}}{2^{|\widehat{\mathcal {C}}_i|}\, \pi } \log \big ( 1+\tfrac{t}{\varepsilon ^2} \big ) \right) \\&= \frac{(\lambda _\varepsilon e^{ \overline{\mathfrak {m}}\, t})^{2n}}{2^{n}} \Big ( \frac{1}{\pi } \log \big ( 1+\tfrac{t}{\varepsilon ^2} \big ) \Big )^{K ( \uppi )} \leqslant \frac{(\lambda _\varepsilon e^{ \overline{\mathfrak {m}}\, t})^{2n}}{2^{n}} M_{ \varepsilon }(t)^{K ( \uppi )} \,, \end{aligned} \end{aligned}$$

where we introduced

$$\begin{aligned} \begin{aligned} M_\varepsilon (t):= \bigg \lceil \frac{1}{\pi } \log \big ( 1+\tfrac{t}{\varepsilon ^2} \big ) \bigg \rceil \,. \end{aligned} \end{aligned}$$

Therefore, we obtain the following upper bound to (5.11):5.12with the expectation taken with respect to the uniform distribution on $S_n$, which has probability mass function $\tfrac{1}{n!}$. Here we used the identity

$$\begin{aligned} \begin{aligned} \text {E}_{S_{n}}\left[ M_\varepsilon (t)^{K(\uppi ) } \right] = \frac{M_{ \varepsilon }(t)^{n}}{n!} + \sum _{\uppi \in S_{n}\setminus \{\text {Id}\}} \frac{ M_{ \varepsilon }(t)^{K (\uppi )}}{n!} \,. \end{aligned} \end{aligned}$$

Hence, we have reduced the problem to studying the generating function of a discrete random variable, namely the total number of cycles in a uniformly at random chosen permutation. Its distribution is a well studied object and we have the explicit identity

$$\begin{aligned} \begin{aligned} \text {E}_{S_{n}}\Big [ M_\varepsilon (t)^{K(\uppi )} \Big ]= \left( {\begin{array}{c}n+M_\varepsilon (t)-1\\ n\end{array}}\right) \end{aligned} \end{aligned}$$

at hand, see e.g. [34, Equation (5.14)]. Thus, we can rewrite (5.12) as5.13Now, we expand the difference on the right-hand side to obtain

5.14 $$\begin{aligned} \frac{(n+M_\varepsilon (t)-1)!}{(M_\varepsilon -1)!}- M_\varepsilon (t)^{n}&= \sum _{j=1}^{n-1} j\, M_\varepsilon (t)^j \prod _{k=j+1}^{n-1} (M_\varepsilon (t)+k)\,. \end{aligned}$$

Note that for every $k =0, \ldots , n-1$

$$\begin{aligned} \begin{aligned} \frac{M_\varepsilon (t)+k}{2\, \log \tfrac{1}{\varepsilon }}&\leqslant \frac{ \tfrac{1}{\pi } \log \big ( 1+\tfrac{t}{\varepsilon ^2} \big )+k+1}{2\, \log \tfrac{1}{\varepsilon }} \leqslant \frac{1}{\pi }\Big (1+\frac{|\log (t+\varepsilon ^2)|+\pi (k+1)}{2\, \log \tfrac{1}{\varepsilon }} \Big )\\&\leqslant \frac{1}{\pi } \exp \left( \frac{|\log (t+\varepsilon ^2)|+\pi (k+1)}{2\,\log \tfrac{1}{\varepsilon }} \right) \,, \end{aligned} \end{aligned}$$

thus, together with (5.13) and (5.14)5.15The terms in the exponent can be estimated uniformly over $\varepsilon$ and $t \in [0, T]$. For the first summand, we make use of (3.23), which yields

$$\begin{aligned} \begin{aligned} (n-1)\frac{|\log (t+\varepsilon ^2)|}{2\, \log \tfrac{1}{\varepsilon }} \leqslant 2n \,. \end{aligned} \end{aligned}$$

Moreover, since $$ n \le 2N_{\varepsilon } = 2 \lfloor \log {\tfrac{1}{\varepsilon }}\rfloor $$

$$\begin{aligned} \begin{aligned} \sum _{k=j}^{n-1} \frac{k+1}{2\, \log { \tfrac{1}{\varepsilon }}} \leqslant \sum _{k=1}^n \frac{k}{2\, \log { \tfrac{1}{\varepsilon }}} = \frac{n(n+1)}{4 \, \log { \tfrac{1}{\varepsilon }} } \leqslant n\,. \end{aligned} \end{aligned}$$

Combining the two estimates with (5.15) yieldswhere we used $n(n-1) \leqslant e^{2n} \leqslant e^{\pi n}$ in the last step. This concludes the proof. $\square$

#### Proof of Lemma 5.11

For convenience let us write $n= 2i ( \tau )$. Our aim is to obtain an upper bound on the total number of parings $\gamma$ that give rise to a given permutation $\uppi \in S_{n}$, via the map $\Pi _{\tau } (\gamma )$ from Definition 4.13. It will be convenient to represent $\uppi$ as a product of permutation cycles $\uppi = \prod _{i =1}^{K(\uppi )} \widehat{\mathcal {C}}_{i}$, for some $K( \uppi ) \in \{ 1, \cdots , n \}$. Here we slightly abuse the notation $\widehat{\mathcal {C}}_{i}$, which is already used in Definition 4.13. Indeed, while the decomposition of a permutation into a product of cycles is unique, the order in which these cycles are chosen is arbitrary. On the other hand, not every order of permutation cycles is admissible in Definition 4.13, as for example cycle $\widehat{\mathcal {C}}_{1}$ is necessarily a cycle among bases of tridents or cherries (because it is the first cycle we extract from a paired tree). In our setting, since we start from an arbitrary permutation $\uppi$, we assume nothing further on $\widehat{\mathcal {C}}_{i}$ other than that they are cycles that decompose $\uppi$. We now provide the desired upper bound via the steps that follow.

1. By Lemma 4.7 we know that any pairing $\gamma$ in $\Pi ^{-1}_\tau (\uppi )$ must contain a $\text {v}$-cycle that alternates between leaves and inner vertices of $[\tau ,\tau ]$. This is because the first $\text {v}$-cycle we extract must be a $\text {v}$-cycle in the paired tree $[\tau , \tau ]_{\gamma }$ (later ones belong instead to trees that are derived from $[\tau , \tau ]_{\gamma }$ by extracting $\text {v}$-cycles). Therefore, there must exist a cycle $\widehat{\mathcal {C}}_{i_{1}}$ that only runs through inner vertices in . Here  and  denote the set of cherries and tridents in $[ \tau , \tau ]$, respectively, see Appendix A.3 for their definition.
  Indeed, if no such cycle exists, then the given permutation cannot arise from any paired tree $[\tau ,\tau ]_\gamma$ using the extraction algorithm $\Pi _\tau$ and the pre-image is the empty set, so that our upper bound holds true. See the discussion below Example 4 for an example of this kind.
2. Since , to construct a $\text {v}$-cycle corresponding to $\widehat{\mathcal {C}}_{i_{1}}$ we must choose for every vertex *v* with label in $\widehat{\mathcal {C}}_{i_{1}}$ an (outgoing) leaf that connects to the next vertex of the $\text {v}$-cycle and an (incoming) leaf that connects to the previous vertex of the $\text {v}$-cycle. Here, which leaf is outgoing and which leaf is incoming matters, leading to at most $2 ! {3 \atopwithdelims ()2} =6$ choices for every vertex (for vertices in  we have six choices, for nodes in  only two). Thus, there are at most $6^{| \widehat{\mathcal {C}}_{i_{1}}|}$ ways to construct a $\text {v}$-cycle through inner vertices labeled by ${\widehat{\mathcal {C}}}_{i_{1}}$.
3. Now proceed iteratively. For $j>1$, we define the set $W^{(j)} \subset \mathcal {I}( [ \tau , \tau ]) \setminus \mathfrak {o}$ as follows: An inner vertex *v* lies in $W^{(j)}$, if and only if
  there exist at least two distinct paths from *v* to leaves in $\mathcal {L}( [ \tau , \tau ])$, which only run through descendants of *v* (away from the root), such that the descendants have been previously extracted, i.e. they lie in $\bigcup _{k=1}^{j-1} \mathcal {I}_{{\widehat{\mathcal {C}}}_{i_{k}}}$,
  and *v* has not been extracted previously, i.e. $v \not \in \bigcup _{k=1}^{j-1} \mathcal {I}_{{\widehat{\mathcal {C}}}_{i_{k}}}$.
  The set $W^{(j)}$ describes the vertices that became “admissible” after extracting the cycles $\{ \widehat{\mathcal {C}}_i \}_{i \in \{ i_{1}, \cdots , i_{j-1} \}}$, meaning that the inner vertices of the next cycle that we extract must belong to $W^{(j)}$.
4. Proceed by choosing a cycle $\widehat{\mathcal {C}}_{i_{j}}$ with vertices in $W^{(j)}$ and counting all possible choices to create a $\text {v}$-cycle with the corresponding nodes as inner nodes.
5. We are done once all cycles have been removed, or we cannot find a cycle that runs through vertices in $W^{(j)}$. In the latter case, we again found a permutation that cannot be obtained as image of the map $\Pi _\tau$ (so the pre-image is empty and the bound trivially true).

In this way, we count all possible pairings that lead to $\uppi$. Overall, either the pre-image is empty and the stated bound is trivially true, or it is bounded by

$$\begin{aligned} 6^{\sum _{i=1}^{n} |{\widehat{\mathcal {C}}}_i|} = 6^{n}\,, \end{aligned}$$

which is the desired bound and concludes the proof. $\square$

### Proof of Proposition 3.3

#### Proof of Proposition 3.3

Fix any $T>0$, $\varepsilon \in (0,\tfrac{1}{T} \wedge \tfrac{1}{2})$ and $\tau \in \mathcal {T}_3^{N_{\varepsilon }}$. Then by Lemma 2.4 and identity (4.6), we can writeBy the triangle inequality and the fact that , see Lemma 5.6, we have that for every $(t,x) \in (0, T] \times {\textbf {R}}^2$5.16The second term on the right-hand side can be directly estimated using Lemma 5.8 as follows:5.17For the first term, by applying Lemma 5.5 and Lemma 5.6 together with the identity , we obtain5.18where in the last step, we used $\Vert P_{t+ \varepsilon ^{2}} \eta (x)\Vert _{L^{2}(\mathbb {P})}= \sqrt{4 \pi (t+ \varepsilon ^{2})}^{- 1}$ and applied Corollary A.2, which yieldswhere we used additionally (3.23) in the first inequality, as well as the bound  in the second inequality.

Now, combining (5.16), (5.17) and (5.18) yieldswith *C* being the constant defined in (3.4), satisfying

$$\begin{aligned} \begin{aligned} C = \frac{6e^{ 2 + 2 \pi }}{\pi } \geqslant \max \left\{ \frac{ 9\, e }{2 \pi } , \frac{6e^{ 2 + 2 \pi }}{\pi } \right\} \,. \end{aligned} \end{aligned}$$

This concludes the proof. $\square$

## Link to a McKean-Vlasov SPDE

This section is dedicated to the proof of Proposition 1.2. Before we do so, we must clarify the meaning of solution to mean-field SPDEs of the form

6.1 $$\begin{aligned} \begin{aligned} \partial _t v = \frac{1}{2} \Delta v +\mathfrak {m}\, v - \alpha \mathbb {E}\left[ v^{2} \right] v \,, \qquad v (0,x) = v_{0} (x)\,, \qquad \forall (t, x) \in (0, \infty ) \times {\textbf {R}}^{2} \;, \end{aligned}\nonumber \\ \end{aligned}$$

for some $\mathfrak {m} \in {\textbf {R}}, \alpha > 0$. To simplify the notation in the next definition, let us write $\mathcal {E}$ for the set of functions $f :{\textbf {R}}^{2} \rightarrow {\textbf {R}}$ such that for some $\lambda (f) > 0$ we have $\sup _{x \in {\textbf {R}}^{2}} | f(x) | e^{- \lambda |x|} < \infty$.

### Definition 6.1

We say that *v* is a solution to (6.1) if – in addition to satisfying the equation – it is smooth on $(0, \infty ) \times {\textbf {R}}^{2}$, and if $x \mapsto \sup _{0 \leqslant s \leqslant T} | v (s, x) | \in \mathcal {E}$ and similarly $x \mapsto \sup _{0 \leqslant s \leqslant T} \mathbb {E}\left[ v^{2} (s, x) \right] \in \mathcal {E}$ for all $T \in (0, \infty )$, $\mathbb {P}$–almost surely.

In this setting, our first result is uniqueness of solutions to (6.1). We observe that well-posedness of Mc-Kean–Vlasov SPDEs of this type *on finite volume* follows for example through the same arguments as in [33]. Yet the extension to infinite volume is not entirely trivial, and as a matter of fact we only prove existence of solutions in a special, Gaussian, case. Instead, our argument for uniqueness works in full generality.

### Proposition 6.2

For any $v_{0} \in \mathcal {E}$ such that

$$\begin{aligned} C_{0} : = \sup _{x \in {\textbf {R}}^{2}} \mathbb {E}[ v_{0}^{2}(x)] < \infty \,, \end{aligned}$$

there exists at most one solution *v* to (6.1) with initial data $v_{0}$.

### Proof

Let $T>0$. We start by proving the following a priori estimate:

6.2 $$\begin{aligned} \sup _{ t \in [0, T] \,,\, x\in {\textbf {R}}^2} \mathbb {E}\big [ v (t,x)^2\,\big ] \leqslant C_{0} e^{2 \overline{\mathfrak {m}} T} =: C_{T} \;. \end{aligned}$$

To establish the above, we start by observing that since *v* is smooth in space and time, $v^2$ solves the initial value problem

$$\begin{aligned} \partial _t v^2&= \frac{1}{2} \Delta v^2 + 2 \mathfrak {m} v^{2} - |\nabla v|^2 -2 \alpha \mathbb {E}\big [ v^2 \big ] \, v^2, \qquad{} & {} (t,x)\in (0,\infty )\times {\textbf {R}}^2 \;, \\ \qquad v^2(0,x)&= v_{0}^2(x)\;,\qquad{} & {} x\in {\textbf {R}}^2 \;. \end{aligned}$$

By the maximum principle one can therefore see that for every $t \geqslant 0$ and $x\in {\textbf {R}}^2$ it holds that

$$\begin{aligned} v^2(t,x) \leqslant \int _{{\textbf {R}}^2} v^2_{0}(x-y) p_t^{(2\mathfrak {m})}(y) \, {\,\text {d}}y \;, \qquad&x\in {\textbf {R}}^2, \end{aligned}$$

and upon taking expectations on both sides we obtain

$$\begin{aligned} \mathbb {E}[ v^{2} (t,x) ] \leqslant C_{0} \int _{{\textbf {R}}^2} p_t^{(2\mathfrak {m})}(y) {\,\text {d}}y = C_{0} e^{ 2\mathfrak {m} t} \;, \end{aligned}$$

which confirms (6.2).

Let us now proceed with the claim of uniqueness. Let *u*, *v* be two solutions to (6.1) with the same initial condition $v_{0}$. We then show that the difference $w (t,x):=u (t,x)-v (t,x)$ is identically equal to zero. The see this, note that *w* satisfies the initial value problem

6.3 $$\begin{aligned} \begin{aligned}&\partial _t w = \frac{1}{2} \Delta w + \mathfrak {m} w - \alpha \Big ( \mathbb {E}\big [ u^2 \big ] - \mathbb {E}\big [ v^2 \big ] \Big ) \, u - \alpha \mathbb {E}\big [ v^2 \big ] \, w,{} & {} \forall (t, x) \in (0, \infty ) \times {\textbf {R}}^{2} \;, \\&w(0,x) = 0 \;,{} & {} \forall x\in {\textbf {R}}^2 \;. \end{aligned} \end{aligned}$$

Denote by $Z(t,x):= \mathbb {E}\big [ u(t,x)^2 \big ] - \mathbb {E}\big [ v(t,x)^2 \big ] = \mathbb {E}\big [ \big (u(t,x) - v(t,x) \big ) \big (u(t,x) + v (t,x) \big ) \big ]$. From Cauchy–Schwarz, it follows that

6.4 $$\begin{aligned} |Z (t,x)|&\leqslant \mathbb {E}\big [ w (t,x)^2 \big ]^{1/2} \, \mathbb {E}\big [ (u +v )^2(t,x) \big ]^{1/2} \leqslant 2\, \sqrt{C_{T}} \,\,\mathbb {E}\big [ w(t,x)^2 \big ]^{1/2 } \;, \end{aligned}$$

where in the last we use the triangle inequality and the a priori estimate (6.2). Then, by the Feynman–Kac formula and in view of the growth assumption on solutions in Definition 6.1, we can express *w* from (6.3) through

$$\begin{aligned} w (t,x) = \alpha \, {\textbf {E}}_x \left[ \int _0^t Z (t-s,\beta (s)) \, u (t-s, \beta (s)) \,\, e^{\mathfrak {m}s- \alpha \int _0^s \mathbb {E}\big [ v (s-r,\beta (r))^2 \big ] {\,\text {d}}r } {\,\text {d}}s\right] \;, \end{aligned}$$

where ${\textbf {E}}_{x}$ indicates the expectation over a Brownian motion $\beta$ (independent from all other random variables) started in $\beta (0) = x$. From here, using (6.4) and bounding the exponential term in the Feynman–Kac representation, it follows that

$$\begin{aligned} | w(t,x) | \leqslant 2 \alpha \, \sqrt{C_{T}} e^{ \overline{\mathfrak {m}} t}\, {\textbf {E}}_x\left[ \int _0^t \mathbb {E} \big [ w(t-s,\beta (s))^2 \big ]^{1/2 } \, | u (t-s, \beta (s)) | \, {\,\text {d}}s\right] , \end{aligned}$$

with $\overline{ \mathfrak {m}} = \max \{ 0, \mathfrak {m} \}$. Next, by Cauchy–Schwarz, we further have the estimate

$$\begin{aligned} | w(t,x) | \leqslant&2 \alpha \, \sqrt{C_{T}}\, e^{ \overline{\mathfrak {m}} t} {\textbf {E}}_x \left[ \int _0^t \mathbb {E} \big [ w(t-s,\beta (s))^2 \big ]\, {\,\text {d}}s\right] ^{1/2} \,\\&{\textbf {E}}_x \left[ \int _0^t u (t-s, \beta (s))^2 \, {\,\text {d}}s \right] ^{1/2}, \end{aligned}$$

and then

$$\begin{aligned} \mathbb {E}\big [ | w(t,x) |^2\big ]&\leqslant 4 \alpha ^{2} C_{T} \, e^{ 2\overline{ \mathfrak {m}} t} {\textbf {E}}_x \left[ \int _0^t \mathbb {E} \big [ w(t-s,\beta (s))^2 \big ]\, {\,\text {d}}s\right] \, \\&\quad \, {\textbf {E}}_x \left[ \int _0^t \mathbb {E} [u (t-s, \beta (s))^2] \, {\,\text {d}}s \right] \\&\leqslant 4 \alpha ^{2} t C_{T}^2 e^{ 2\overline{ \mathfrak {m}} t} \, {\textbf {E}}_x \left[ \int _0^t \mathbb {E} \big [ w(t-s,\beta (s))^2 \big ]\, {\,\text {d}}s \right] \\&= 4 \alpha ^{2}t C_{T}^2 e^{ 2\overline{ \mathfrak {m}} t} \int _0^t \int _{{\textbf {R}}^2} \mathbb {E} \big [ w(t-s,y)^2 \big ]\, p_s(y-x) \, {\,\text {d}}y \, {\,\text {d}}s \;, \end{aligned}$$

where in the second inequality we used, again, the a priori bound (6.2). Taking the supremum over the *x* variable on both sides we conclude

$$\begin{aligned} \begin{aligned} \sup _{x \in {\textbf {R}}^{2}} \mathbb {E}[ | w (t, x) |^{2} ] \leqslant 4 \alpha ^{2} t C_{T}^{2} e^{ 2 \overline{\mathfrak {m}} t} \int _{0}^{t} \sup _{x \in {\textbf {R}}^{2}} \mathbb {E}[| w (t-s, x) |^{2}] {\,\text {d}}s \;. \end{aligned} \end{aligned}$$

Now, setting $F(t):= \sup _{x \in {\textbf {R}}^{2}} \mathbb {E} | w(t,x) |^2$, we conclude that for every $T>0$ and $0<t \leqslant T$

$$\begin{aligned} F(t) \leqslant \overline{C}_T \int _0^t F(s) \, {\,\text {d}}s,\qquad \text {where} \quad \overline{C}_T:=4 \alpha ^{2} T C_{T}^2 e^{2 \overline{\mathfrak {m}} t} \;. \end{aligned}$$

Since $F(0)=0$, it follows that $F(t)=0$ for every $0<t \leqslant T$ and $T>0$. $\square$

We are now ready for the main result of this section.

### Proof of Proposition 1.2

In the particular case of $v_{0} = p_{\varepsilon ^{2}} \star \eta$ and $\alpha = \frac{ 3 }{\log { \frac{1}{\varepsilon }}}$, we can check that the McKean–Vlasov equation (6.1) admits the solution

$$\begin{aligned} \begin{aligned} v_{ \hat{\lambda }, \varepsilon }(t,x) := \hat{\lambda }\, \sigma _{\hat{\lambda }, \varepsilon } (t) P_{t}^{(\mathfrak {m})} (p_{\varepsilon ^{2}} \star \eta )(x) \;, \end{aligned} \end{aligned}$$

where $\sigma _{\hat{\lambda }, \varepsilon }$ is the solution to the ODE

6.5 $$\begin{aligned} \begin{aligned} \partial _{t} \sigma _{ \hat{\lambda }, \varepsilon } = - \frac{3 \hat{\lambda }^{2}}{ \log { \tfrac{1}{\varepsilon }}}\frac{ e^{2 \mathfrak {m} t}}{4 \pi (t + \varepsilon ^{2})} \sigma _{ \hat{\lambda }, \varepsilon }^{3} \;, \quad \sigma _{\hat{\lambda }, \varepsilon } (0) = 1. \end{aligned} \end{aligned}$$

For this, let us substitute $v_{ \hat{\lambda }, \varepsilon }$ into the mild formulation of (6.1), which yields

$$\begin{aligned} \begin{aligned}&\hat{\lambda }\, \sigma _{\hat{\lambda }, \varepsilon } (t) P_{t}^{(\mathfrak {m})} (p_{\varepsilon ^{2}} \star \eta )(x)\\&\quad = \hat{\lambda }\, P_{t}^{(\mathfrak {m})} (p_{\varepsilon ^{2}} \star \eta )(x)\\&\qquad - \frac{ 3 \hat{\lambda }^{3} }{\log { \tfrac{1}{\varepsilon }}} \int _{0}^{t} \sigma _{\hat{\lambda }, \varepsilon } (s)^{3} \int _{{\textbf {R}}^{2}} p_{t-s}^{(\mathfrak {m})}(y-x) \mathbb {E}\big [ | P_{s}^{(\mathfrak {m})} (p_{\varepsilon ^{2}} \star \eta )(y) |^{2} \big ] \\&\quad P_{s}^{(\mathfrak {m})} (p_{\varepsilon ^{2}} \star \eta )(y) {\,\text {d}}y {\,\text {d}}s\\&\quad = \hat{\lambda }\, P_{t}^{(\mathfrak {m})} (p_{\varepsilon ^{2}} \star \eta )(x) - \frac{ 3 \hat{\lambda }^{3} }{\log { \tfrac{1}{\varepsilon }}} \left\{ \int _{0}^{t} \sigma _{\hat{\lambda }, \varepsilon } (s)^{3} \frac{ e^{2\mathfrak {m} \, s}}{4 \pi (s+ \varepsilon ^{2})} {\,\text {d}}s\right\} \; P_{t}^{(\mathfrak {m})} (p_{\varepsilon ^{2}} \star \eta )(x)\,, \end{aligned} \end{aligned}$$

where we used that $\mathbb {E}\big [ | P_{s}^{(\mathfrak {m})} (p_{\varepsilon ^{2}} \star \eta )(y) |^{2} \big ] = e^{2 \mathfrak {m}\, s} (4 \pi (s + \varepsilon ^{2}))^{-1}$ and integrated out the spatial variable using Chapman–Kolmogorov. Now, dividing by $\hat{\lambda }\,P_{t}^{(\mathfrak {m})} (p_{\varepsilon ^{2}} \star \eta )(x)$ on both sides and taking the temporal derivative yields (6.5). To verify that $v_{ \hat{\lambda }, \varepsilon }$ is a solution in the sense of Definition 6.1, it now suffices to check that $\sup _{0 \leqslant s \leqslant T} | p_{s+\varepsilon ^{2}} \star \eta | \in \mathcal {E}$ for any $\varepsilon , T > 0$. This follows for example because $\mathbb {E}\big [ \sup _{| x |_{\infty } \leqslant 1} \sup _{0 \leqslant s \leqslant T}| p_{s+\varepsilon ^{2}} \star \eta (x)| \big ] < \infty$ (where $| x |_{\infty } = \max _{i=1,2} | x_{i} |$), so that

$$\begin{aligned} \mathbb {E}\left[ \sum _{z \in {\textbf {Z}}^{2}} \frac{\sup _{| x - z |_{\infty } \leqslant 1} \sup _{0 \leqslant s \leqslant T} | p_{s+\varepsilon ^{2}} \star \eta (x) |}{1 + |z|^3} \right] < \infty \;,\end{aligned}$$

which yields the desired result (cf. [24, Lemma 1.1]).

Now, the differential equation (6.5) admits the explicit solution

6.6 $$\begin{aligned} \begin{aligned} {\sigma }_{ \hat{\lambda }, \varepsilon }(t) = \frac{1}{\sqrt{1 + \frac{3 \hat{\lambda }^{2}}{ \pi } c_{ \varepsilon }(t) }} \,, \qquad \text {with} \qquad c_{ \varepsilon }(t):= \frac{1}{ 2 \log { \frac{1}{ \varepsilon }}} \int _{0 }^{t } \frac{ e^{2 \mathfrak {m} s}}{s + \varepsilon ^{2}} {\,\text {d}}s \,. \end{aligned} \end{aligned}$$

Moreover, the solution $$v_{ \hat{\lambda }, \varepsilon }$$ to (6.1) is unique, which follows from Proposition 6.2. Finally, by Lemma A.1, we have that $$c_{ \varepsilon }(t) \rightarrow 1 $$ as $$ \varepsilon \rightarrow 0$$, for every $$ t>0$$. Hence,

$$\begin{aligned} \begin{aligned} \lim _{ \varepsilon \rightarrow 0} \sigma _{ \hat{\lambda }, \varepsilon }(t) = \frac{1}{\sqrt{1 + \frac{3 \hat{\lambda }^{2}}{ \pi } }}= \sigma _{ \hat{\lambda }} \qquad \forall t >0 \,. \end{aligned} \end{aligned}$$

This completes the proof. $\square$

## Data Availability

No datasets were generated or analysed during the current study.

## Appendix

### On the exponential integral

Throughout the paper, a crucial ingredient is to understand the small-$\varepsilon$ behaviour of the integral

In this appendix we will prove some useful estimates concerning this integral.

#### Lemma A.1

Let $\mathfrak {m} \in {\textbf {R}}$ and $t \in (0, \infty )$, then

$$\begin{aligned} \begin{aligned} \left| \frac{1}{2 \log { \frac{1}{ \varepsilon }}} \int _{0}^{t} \frac{e^{2 \mathfrak {m} \, s}}{s+ \varepsilon ^{2}} {\,\text {d}}s -1 \right| \leqslant \frac{ e^{ 2|\mathfrak {m}|\, t} + | \log {(t + \varepsilon ^{2})} | }{2 \log { \frac{1}{ \varepsilon }}} \,, \end{aligned} \end{aligned}$$

for every $\varepsilon \in (0,\tfrac{1}{2})$, with $\overline{\mathfrak {m}} = \max \{ \mathfrak {m}\;, 0 \}$. In particular, for every $t \in (0, \infty )$

A.1

#### Proof

First expanding the exponential, we write

$$\begin{aligned} \begin{aligned} \int _{0}^{t} \frac{e^{2\mathfrak {m}\, s}-1}{s+ \varepsilon ^{2}} {\,\text {d}}s&= \int _{0}^{t} \frac{ s}{s + \varepsilon ^{2}} \sum _{k=1}^{ \infty } \frac{(2\mathfrak {m})^{k}\, s^{k-1}}{k!} {\,\text {d}}s\,. \end{aligned} \end{aligned}$$

Thus, we obtain

$$\begin{aligned} \begin{aligned} \left| \int _{0}^{t} \frac{e^{2 \mathfrak {m}\, s}-1}{s+ \varepsilon ^{2}} {\,\text {d}}s \right| \leqslant \sum _{k=1}^{ \infty } \int _{0}^{t} \frac{|2 \mathfrak {m}|^{k}\, s^{k-1}}{k!} {\,\text {d}}s \leqslant e^{2 | \mathfrak {m}|\, t} \,. \end{aligned} \end{aligned}$$

In addition, we have

$$\begin{aligned} \begin{aligned} \left| \frac{1}{2 \log { \frac{1}{ \varepsilon }}} \int _{0}^{t} \frac{1}{s+ \varepsilon ^{2}} {\,\text {d}}s -1 \right| = \frac{| \log {( t+ \varepsilon ^{2} )} |}{ 2 \log { \frac{1}{ \varepsilon }}}\,, \end{aligned} \end{aligned}$$

so that the statement follows from the triangle inequality. The second part of the statement is now an immediate consequence, since

This completes the proof. $\square$

#### Corollary A.2

Let $\mathfrak {m} \in {\textbf {R}}$, $T>0$ and $k \in {\textbf {N}}$, then

$$\begin{aligned} \begin{aligned} \left| \left( \frac{1}{2 \log { \frac{1}{ \varepsilon }}} \int _{0}^{t} \frac{e^{2 \mathfrak {m} \, s}}{s+ \varepsilon ^{2}} {\,\text {d}}s \right) ^{k} - 1 \right| \leqslant k \, \left( 3e^{ 2\overline{\mathfrak {m}}\, t} \right) ^{k-1} \frac{ e^{ 2|\mathfrak {m}|\, t} + | \log {(t + \varepsilon ^{2})} | }{2 \log { \frac{1}{ \varepsilon }}} \,, \end{aligned} \end{aligned}$$

for every $t \in [0,T]$ and $\varepsilon \in (0,\tfrac{1}{T}\wedge \tfrac{1}{2})$.

#### Proof

Using a first order Taylor expansion of the monomial of order *k* around 1, we have

A.2 $$\begin{aligned}{} & {} \left| \left( \frac{1}{2 \log { \frac{1}{ \varepsilon }}} \int _{0}^{t} \frac{e^{2 \mathfrak {m} \, s}}{s+ \varepsilon ^{2}} {\,\text {d}}s \right) ^{k} - 1 \right| \nonumber \\{} & {} \quad \leqslant k \,\left\{ \sup _{x \in [1,b_{\varepsilon }(t) \vee 1]} x^{k-1} \right\} \left| \frac{1}{2 \log { \frac{1}{ \varepsilon }}} \int _{0}^{t} \frac{e^{2 \mathfrak {m} \, s}}{s+ \varepsilon ^{2}} {\,\text {d}}s -1 \right| \,, \end{aligned}$$

with

$$\begin{aligned} \begin{aligned} b_{ \varepsilon }(t) := \frac{1}{2 \log { \frac{1}{ \varepsilon }}} \int _{0}^{t} \frac{e^{2 \mathfrak {m} \, s}}{s+ \varepsilon ^{2}} {\,\text {d}}s \leqslant e^{2 \overline{\mathfrak {m}} \, t} \left( 1+ \frac{| \log {(t+ \varepsilon ^{2})} |}{ 2 \log { \frac{1}{\varepsilon }} } \right) \,. \end{aligned} \end{aligned}$$

In particular, we find that

$$\begin{aligned} \begin{aligned} \sup _{x \in [1,b_{\varepsilon }(t) \vee 1]} x^{k-1} \leqslant e^{2(k-1) \overline{\mathfrak {m}} \, t} \left( 1+ \frac{| \log {(t+ \varepsilon ^{2})} |}{ 2 \log { \frac{1}{\varepsilon }} } \right) ^{k-1} \leqslant \big ( 3 e^{2 \overline{\mathfrak {m}} \, t} \big )^{k-1} \,, \end{aligned} \end{aligned}$$

where we used (3.23) in the last step. The statement follows now by upper bounding the last term in (A.2) via Lemma A.1. $\square$

### Symmetric functions and trees

#### Lemma A.3

For a tree $\tau \in \mathcal {T}$ of the form $\tau = [\tau _1\ \cdots \ \tau _n]$ such that $\mathcal {I}:= \mathcal {I}( \tau ) \setminus \mathfrak {o}_{ \tau }\ne \emptyset$ and any symmetric function $\Psi :{\textbf {R}}^{\mathcal {I}}\rightarrow {\textbf {R}}$, we have that

with the domain $\mathfrak {D}_{\tau }(t)$ defined in (4.24) and  denoting the trimmed tree defined in (2.18).

#### Proof

We prove the statement by induction over $i ( \tau ) \geqslant 2$. Note that the case $i ( \tau )=1$ is excluded as the single inner vertex must necessarily be the root. If $i (\tau ) = 2$, then $\tau$ must be of the form  and $\mathcal {I}=\{v\}$ for some vertex *v*. Then

$$\begin{aligned} \begin{aligned} \int _{\mathfrak {D}_{\tau }(t)} \Psi ( s_{\mathcal {I}}) {\,\text {d}}s_{\mathcal {I}} = \int _{0}^{t} \Psi ( s_{v}) {\,\text {d}}s_{v} \,, \end{aligned} \end{aligned}$$

which is the desired statement since . Now assume that the statement holds true for any tree $\tau '= [ \tau '_{1} \ \cdots \tau '_{n'}]$ such that $i ( \tau ') \leqslant N$, for some $N \geqslant 2$, and write $\mathcal {I}' := \mathcal {I}( \tau ') \setminus \mathfrak {o}'$ with $\mathfrak {o}' := \mathfrak {o}_{ \tau '}$. Let $\tau \in \mathcal {T}$ be of the form , so that . Then

where we used the induction hypothesis and the fact that $\Psi (s_{\mathfrak {o}'}, \cdot ) :{\textbf {R}}^{\mathcal {I}^{\prime }} \rightarrow {\textbf {R}}$ is a symmetric function. Using the symmetry of the function $\Psi$, we further see that the identification of variable $s_{\mathfrak {o}'}$ as the maximum variable is irrelevant with regards to the integration, and the assignment of any of the variables $s_{\mathcal {I}}$ as being the maximum would result in the same value. Thus, the above is equal to

and the statement follows now for $\tau$ because . Furthermore, we notice that

$$\begin{aligned} \begin{aligned} \int _{\mathfrak {D}_{\tau }(t)} \Psi ( s_{\mathcal {I}}) {\,\text {d}}s_{\mathcal {I}} = \int _{\mathfrak {D}_{[\tau ']}(t)} \Psi ( s_{\mathcal {I}( \tau ')}) {\,\text {d}}s_{\mathcal {I}( \tau ')} \;, \end{aligned} \end{aligned}$$

meaning that the additional occurrences of  in the grafting of $\tau$ do not affect the integral. Finally, consider the case $\tau \in \mathcal {T}$ such that $i ( \tau ) = N+1$, with , $k \geqslant 2$ and . Again the extra occurrences of  in the grafting of $\tau$ do not affect the value of the integral. Moreover, the integration domain $\mathfrak {D}_{\tau }(t)$ can be written as an union of sub-tree-simplices:

$$\begin{aligned} \begin{aligned} \int _{\mathfrak {D}_{\tau }(t)} \Psi ( s_{\mathcal {I}}) {\,\text {d}}s_{\mathcal {I}} = \int _{\mathfrak {D}_{[\tau _{1}]}(t)} \cdots \int _{\mathfrak {D}_{[\tau _{k}]}(t)} \Psi ( s_{\mathcal {I}}) {\,\text {d}}s_{\mathcal {I}( \tau _{k})} \cdots {\,\text {d}}s_{\mathcal {I}( \tau _{1})}\,. \end{aligned} \end{aligned}$$

The statement follows from the induction hypothesis, since the restriction of $\Psi$ to a subset of variables remains a symmetric function. $\square$

### Proof of Lemma 4.7

Here we prove Lemma 4.7, which guarantees the existence of a $\text {v}$-cycle in the gluing of two ternary trees. First, let us introduce some notation. For $\tau \in \mathcal {T}_{ \leqslant 3}$, we partition the subset of inner vertices neighbouring leaves in $\mathcal {L}( \tau )$ as follows. Let

- be the subset of inner vertices $v \in \mathcal {I}( \tau )$ that is a *basis of a trident*, i.e. there exist exactly three $u_{1}, u_{2}, u_{3} \in \mathcal {L}( \tau )$ such that $\mathfrak {p}(u_{i})=v$.
- be the subset of inner vertices $v \in \mathcal {I}( \tau )$ that is a *basis of a cherry*, i.e. there exist exactly two $u_{1}, u_{2} \in \mathcal {L}( \tau )$ such that $\mathfrak {p}(u_{i})=v$.
- be the subset of inner vertices $v \in \mathcal {I}( \tau )$ that is a *basis of a lollipop*, i.e. there exist exactly one $u \in \mathcal {L}( \tau )$ such that $\mathfrak {p}(u)=v$. In the following, we will call elements in  **dead-ends**.

#### Proof

Let us start by noting that if $[\tau _1,\tau _2]$ does not contain any dead-ends, then the paired tree $[\tau _1,\tau _2]_\gamma$ contains a $\text {v}$-cycle. To see this, first notice that every leaf of $[\tau _1,\tau _2]$ belongs either to a cherry or to a trident. Now, consider an arbitrary leaf, call it $v_0$, and let $v_1$ be the unique leaf in $[\tau _1,\tau _2]$, which is connected to $v_0$ via $\gamma$. If $v_1$ is inside the same cherry or trident component as $v_0$, then we have already identified a $\text {v}$-cycle, which in this case is of length 1. If not, then let $v_2$ be a different leaf inside the same cherry or trident component as that of $v_1$ and denote by $v_3$ the leaf which is connected to $v_2$ via $\gamma$. Again, if $v_2$ and $v_3$ fall inside the same component (which in this case would necessarily be a trident), then a $\text {v}$-cycle comprising of leaves $v_2,v_3$ and the corresponding base point of the trident is identified. If not, then continue the procedure. Since there is only a finite number of leaves, we will either encounter somewhere in the process a $\text {v}$-cycle of length 1, or the path will return to a component previously visited during this process, thus identifying a $\text {v}$-cycle. Diagrammatically, we have the following representation:

where sub-trees $\sigma _1, \sigma _1,\sigma _3$ may be identical to just a single leaf, i.e. , and even though we did not include them, there are $\gamma$ links emanating from the leaves of these trees.

We will next reduce the case that $[\tau _1,\tau _2]$ contains also dead-ends to a situation of no dead-ends. Dead-ends present a problem: When tracing contractions in $\gamma$, we may hit a dead-end and thus are not able to continue to complete a $\text {v}$-cycle. What we will show is that by eliminating paths that start from a dead-end, the resulting sub-graph is one that consists of only cherries and tridents linked through $\gamma$. Thus, a $\text {v}$-cycle exists within this sub-graph by the previous argument.

Let us start by picking an arbitrary dead-end of $[\tau _1,\tau _2]$. Call $v_0$ its associated leaf and suppose it connects via $\gamma$ to another leaf of $[\tau _1,\tau _2]$, which we call $v_1$. Now, remove this connection as follows:

where again we understand that, even not shown, there are $\gamma$-links emanating from trees $\sigma _1,\sigma _2,\sigma _3,\sigma _4$. Moreover, we agree that neither $\sigma _1$ nor $\sigma _2$ equals  (they might be $\emptyset$, though), so that this part of the tree corresponds to a dead-end, while $\sigma _3,\sigma _4$ might be comprising a .

In the resulting (contracted) tree on the right-hand side, we distinguish three cases:

1. Neither of $\sigma _3$ and $\sigma _4$ are single leaves. In this case we have eliminated two dead-ends, while not affecting the number of cherries and tridents.
2. Only one of the $\sigma _3$ and $\sigma _4$ is a . In this case, we have eliminated a dead-end in the left part of the tree, while we have also created a new dead-end in the right part, corresponding to either $\sigma _3$ or $\sigma _4$, whichever happens to be the . In this case, we have also reduced by one the number of cherries (by eliminating the cherry that was present in the right part of the sub-tree) but, nevertheless, we have not reduced the number of tridents.
3. Both $\sigma _3$ and $\sigma _4$ are . In this case, in the right part of the tree we had, before the elimination, a trident. Thus, after the elimination we reduced both the number of dead-ends and tridents by one, while the number of cherries actually increased by one.

As we will prove in Lemma A.4 below, the total number of tridents in $[\tau _1,\tau _2]$ is strictly larger than the number of dead-ends. In all three cases above, the elimination procedure preserves this inequality. Indeed, in Case 1. the number of dead-ends is reduced by 2 while the number of tridents remains the same, in Case 2. both the number of tridents and dead-ends remain the same (the number of cherries is reduced by one but this has no effect) and in Case 3. both the number of tridents and dead-ends is reduced by 1 (the number of cherries increases by 1). Thus, continuing to eliminate dead-ends, we necessarily end up with a sub-tree of $[\tau _1,\tau _2]_\gamma$, which will only contain cherries and tridents. We can now return to the beginning of the proof and the situation of a (sub-ternary) tree that consists of only tridents and cherries, which necessarily contains a $\text {v}$-cycle. $\square$

#### Lemma A.4

Let , then .

#### Proof

Clearly the statement is true for . Any larger tree in $\mathcal {T}_3$ can be constructed from  by successively gluing tridents  onto leaves. Now, there are three possibilities for a trident to be glued onto an existing tree in $\tau \in \mathcal {T}_{3}$, as described in the table below.

**Table:**

Pre-gluing	Post-gluing		
		$+0$	$+0$
		$+1$	$+1$
		$-1$	$+1$

Here,  are placeholders for corresponding sub-trees. In all three cases the claimed inequality remains true as we can only create a new dead-end by creating a trident at the same time. $\square$

### Extracting the tree simplex

In the proof of Lemma 4.14, we eventually integrate time over indicator functions which we collected from the estimate (4.16). The following lemma states that the restrictions imposed by these indicator functions agree with the corresponding tree-simplex (4.24). In particular, the cycle removal estimate in Lemma 4.14 is independent of the chosen pairing.

#### Lemma A.5

Let $$ t>0$$, $$ \tau \in \mathcal {T}_{3}$$ and write $$ \mathcal {I}:= \mathcal {I}( [ \tau , \tau ]) \setminus \mathfrak {o}$$. Then for every pairing $$ \gamma \in \mathcal {Y}( \tau , \tau )$$, we have

A.3 $$\begin{aligned} \begin{aligned} {[}0,t]^{\mathcal {I}}\cap \bigcap _{i=1}^{K( \tau , \gamma )} \bigcap _{v \in \mathcal {I}_{\mathcal {C}_{i}}} \{ s_{\mathfrak {d}_{ \sigma _{i}, \gamma } (v)} \leqslant s_v \leqslant s_{ \mathfrak {p}_{ \sigma _{i}}(v)} \} = \mathfrak {D}_{ [ \tau , \tau ]}(t) \,, \end{aligned} \end{aligned}$$

where $( \mathcal {C}_{k} )_{k =1}^{K (\tau , \gamma )}$ denotes the sequence of $\text {v}$-cycles and $(\sigma _{i})_{i=1}^{K ( \tau , \gamma )}$ the sequence of reduced trees, constructed from $[\tau ,\tau ]_\gamma$ via the cycle extraction map (Definition 4.12). The set $\mathfrak {D}_{[ \tau , \tau ]}(t)$ was defined in (4.24).

#### Proof

Let $v \in \mathcal {I}$ be arbitrary. Then there exists an $i =1,\ldots , K ( \tau , \gamma )$ such that $v \in \mathcal {I}_{\mathcal {C}_{i}}$ and we define $u_{1}:= \mathfrak {d}_{ \sigma _{i}, \gamma }(v)$, $w_{1}:= \mathfrak {p}_{ \sigma _{i}}(v)$. Notably, there exists a unique path $(u_{1},\ldots , u_{m}, v, w_{m'} ,\ldots , w_{1})$ in the tree $[ \tau , \tau ]$ with $u_{j},w_{j} \in \mathcal {I}$, $j \geqslant 2$.

If $$s_{\mathcal {I}} \in \mathfrak {D}_{ [ \tau , \tau ]}(t) $$, then

$$\begin{aligned} \begin{aligned} 0 \leqslant s_{ \mathfrak {d}_{ \sigma _{i}, \gamma }(v)}=s_{u_{1}} \leqslant \cdots \leqslant s_{u_{m}} \leqslant s_{v} \leqslant s_{w_{m'}} \leqslant \cdots \leqslant s_{w_{1}}= s_{\mathfrak {p}_{ \sigma _{i}}(v)} \leqslant t \,, \end{aligned} \end{aligned}$$

which implies in particular that $s_{ \mathfrak {d}_{ \sigma _{i}, \gamma }(v)} \leqslant s_{v} \leqslant s_{\mathfrak {p}_{ \sigma _{i}}(v)}$.

On the other hand, the vertex $\mathfrak {p} (v)= \mathfrak {p}_{[ \tau , \tau ]}(v) = w_{m'}$ lies in $\mathcal {I}_{\mathcal {C}_{i'}}$ for some $i'=1,\ldots , K ( \tau , \gamma )$. Thus, if $s_{\mathcal {I}}$ lies in the left-hand side of (A.3), then

- either the parent of *v* has not been removed by the cycle extraction map in an earlier iteration, i.e. $i' \geqslant i$, in which case
  $$\begin{aligned} \begin{aligned} s_{v} \leqslant s_{\mathfrak {p}_{ \sigma _{i}} (v)}= s_{w_{m'}} = s_{\mathfrak {p} (v)} \,, \end{aligned} \end{aligned}$$
- or the parent of *v* has been removed in a previous iteration, i.e. $$ i' < i$$, then
  $$\begin{aligned} \begin{aligned} s_{v}= s_{\mathfrak {d}_{ \sigma _{i'}, \gamma }(w_{m'})} \leqslant s_{w_{m'}} = s_{\mathfrak {p} (v)}\,. \end{aligned} \end{aligned}$$

As the choice of *v* was arbitrary, this concludes the proof. $\square$

## Footnotes

1 The choice $\varepsilon < 1/2$ is arbitrary to avoid issues with the blow-up at $\varepsilon =1$ of the logarithm.

2 “marginally” refers to the fact that the nonlinearity has neither a dominant nor a negligible effect as seen via the renormalisation scaling $u^{\delta }(t, x) = \delta u ( \delta ^{2} t, \delta x)$ and the invariance in (1.2).

3 We note that this estimate, via triangle inequality and dropping part of the exponential term is not expected to be optimal and is the place where we lose. This leads subsequently to the need of a growing $N_\varepsilon$ and the restrictions to the time horizon.

4 In the $\emptyset$-contracted tree $\tau _\emptyset$, the iterated stochastic integral will correspond to a *homogeneous chaos*, and the purpose of the $\emptyset$ subscript in its graphical depiction is to distinguish it from the graph representation (4.2), which corresponds to an element of the *inhomogeneous chaos*. On the other hand, there is no such danger of confusion in the rest of the graphical depictions of $\tau _\kappa$ with $\kappa \ne \emptyset$ and so we do not use any similar subscript in order not to overload notation.

5 Note that we will always find vertices $$ w_{0}, w_{2}$$ (which might agree with $$ v_{0}, v_{2} $$), since going down we will always hit the root last (which is not part of $$\mathcal {C}$$), and going up we can always proceed because the sub-trees are ternary.

6 Because on the right-hand side we sum over all non-contributing pairings, which may consist of one contributing contraction and one contraction that doesn’t contribute, we overestimate the left-hand side, which is the reason for the inequality.
